# Ants (Insecta, Hymenoptera, Formicidae) from the Floresta da Tijuca sector, Parque Nacional da Tijuca, Rio de Janeiro, Brazil

**DOI:** 10.3897/BDJ.12.e132248

**Published:** 2025-03-13

**Authors:** Márcio Morais Silva, Antônio J. Mayhé Nunes, Felipe F. F. Moreira

**Affiliations:** 1 Fundação Oswaldo Cruz, Programa de Pós-graduação em Biodiversidade e Saúde, Rio de Janeiro, Brazil Fundação Oswaldo Cruz, Programa de Pós-graduação em Biodiversidade e Saúde Rio de Janeiro Brazil; 2 Fundação Oswaldo Cruz, Instituto Oswaldo Cruz, Laboratório de Entomologia, Rio de Janeiro, Brazil Fundação Oswaldo Cruz, Instituto Oswaldo Cruz, Laboratório de Entomologia Rio de Janeiro Brazil; 3 Universidade Federal Rural do Rio de Janeiro, Seropédica, Brazil Universidade Federal Rural do Rio de Janeiro Seropédica Brazil

## Abstract

**Background:**

Ants (Insecta, Hymenoptera, Formicidae) are social insects and one of the most abundant groups of animals. Their colonies are established in a wide range of habitats, such as in cavities in the ground, litter on the forest floor or in rocky cracks. Currently, there are approximately 15,000 valid species of ants, of which about 6,000 are recorded from the Neotropical Region. The Atlantic Forest is one of the biomes with the greatest biodiversity, showing a high degree of endemism. However, it has suffered severe impacts and is now reduced to a fraction of the original area. One of the most significant remnants of the Atlantic Forest is the Parque Nacional da Tijuca (PNT), a protected area located in the state of Rio de Janeiro, Brazil. There is limited knowledge about the myrmecofauna of the PNT, with most research efforts focused on the Serra da Carioca sector.

**New information:**

In the present study, we performed the first inventory of the mirmecofauna of the Floresta da Tijuca sector of the PNT, using pitfall traps and Winkler extractors in areas with three different degrees of preservation. In total, 80 species are recorded from Floresta da Tijuca, of which 18 are reported for the first time from this sector, 37 from the PNT, 10 from the State of Rio de Janeiro and one from the Atlantic Forest biome. These results increase the known diversity of ants of the PNT from 149 to 200 species. Furthermore, the known geographic distribution of *Hypoponeraviri* (Santschi, 1923) is extended northwards, those of *Holcoponeramina* Brown, 1956 and *Neocerapachysneotropicus* Weber, 1939, southwards and *Brachymyrmexbruchi* Forel, 1912 and *Hypoponeraparva* (Forel, 1909), eastwards. The most abundant species in our samples was *Holcoponeramoelleri* (Forel, 1912), followed by *Pachycondylastriata* F. Smith, 1858, *Strumigenysdenticulata* Mayr, 1887, *Hypoponeratrigona* (Mayr, 1887), *Megalomyrmexgoeldii* Forel, 1912 and *Hylomyrmareitteri* (Mayr, 1887). The primary forest area was the richest, followed by the disturbed and advanced restoration areas. The species richness was higher in the primary forest and advanced restoration areas during the dry season, while it was higher in the disturbed area during the rainy season.

## Introduction

Ants (Insecta, Hymenoptera, Formicidae) are social insects and one of the most abundant and widely distributed groups of animals, being absent only from polar regions (Hölldobler and Wilson 1990). Ant colonies are established in a wide range of habitats, such as cavities in the ground, layers of branches and litter on the forest floor or in rocky cracks. In the canopy, they usually establish mutualistic associations with plants, offering protection against herbivory in exchange for shelter and/or food (Janicki et al. 2016, Guénard 2017). They represent a large portion of the terrestrial organism abundance in forests, where several environmental services are associated with their biology (Hölldobler and Wilson 1990, Elizalde et al. 2020).

Currently, there are approximately 15,000 valid species of ants, of which about 6000 have been recorded from the Neotropical Region (Kass et al. 2022, Bolton 2024). Brazil is especially rich in ants due to its continental dimensions and great diversity of biomes and ecosystems, which provide many different niches that are occupied by these insects (Silva et al. 2022). However, despite being in one of the most studied bioregions in the world, there are still many regions of the country that are poorly explored or even completely unexplored (Feitosa et al. 2022).

Located mainly within Brazil, the Atlantic Forest is considered a biodiversity hotspot, with high degrees of species richness and endemism (Wilson et al. 2021). Since the 16^th^ century, this biome has been suffering impacts caused by the exploration of natural resources, forest fragmentation, land use for monocultures and the founding and expansion of cities (Rezende et al. 2018). The myrmecofauna of the Atlantic Forest has been studied from different perspectives, such as in the use as a bioindicator (Ribas et al. 2012), population dynamics (Bihn et al. 2008) and provision of ecological services (Garcia et al. 2020), amongst others. Nonetheless, the local species richness might still be underestimated, mainly because many areas within this biome are difficult to access or have not been sufficiently sampled (Carlucci et al. 2021). Here, we contribute to reduce such knowledge gap by carrying out the first survey of the myrmecofauna of the Floresta da Tijuca sector, Parque Nacional da Tijuca, Rio de Janeiro, Brazil.

## Materials and methods

### Study area

The Parque Nacional da Tijuca (PNT) (22°55'–23°00' S, 043°11'–043°19' W) is an environmental protection area located in the Atlantic Forest biome and divided into four geographic sectors, namely Serra da Carioca, Pedra da Gávea and Pedra Bonita, Pretos Forros and Covanca and Floresta da Tijuca (Ministério do Meio Ambiente 2008). The PNT is one of the largest urban parks in the world, occupying 3953 hectares or 3.5% of the area of the Municipality of Rio de Janeiro, State of Rio de Janeiro, Brazil. The average annual temperature is 22°C and the average annual rainfall is 2250 mm (Miranda and Avelar 2021). The predominant vegetation is a typical tropical rainforest, characterised by large trees, palms, ferns, epiphytes and lianas (Freitas et al. 2006, Varassin et al. 2021). Since the colonisation by the Portuguese, the region suffered severe impacts due to logging and farming, leading to the removal of a large portion of the natural vegetation and the loss of biodiversity. Then, during the second half of the 19^th^ century, a reforestation process started, aiming to preserve the water supply for the City of Rio de Janeiro (Araujo and Avelar 2018). The establishment and regulation of the area as a national park happened in 1961 and, since then, it serves as a refuge and for the re-introduction of native plant and animal species (Ministério do Meio Ambiente 2008, Silva and Brandão 2020, Mittelman et al. 2021).

### Sampling and analysis

**Figure 1.**
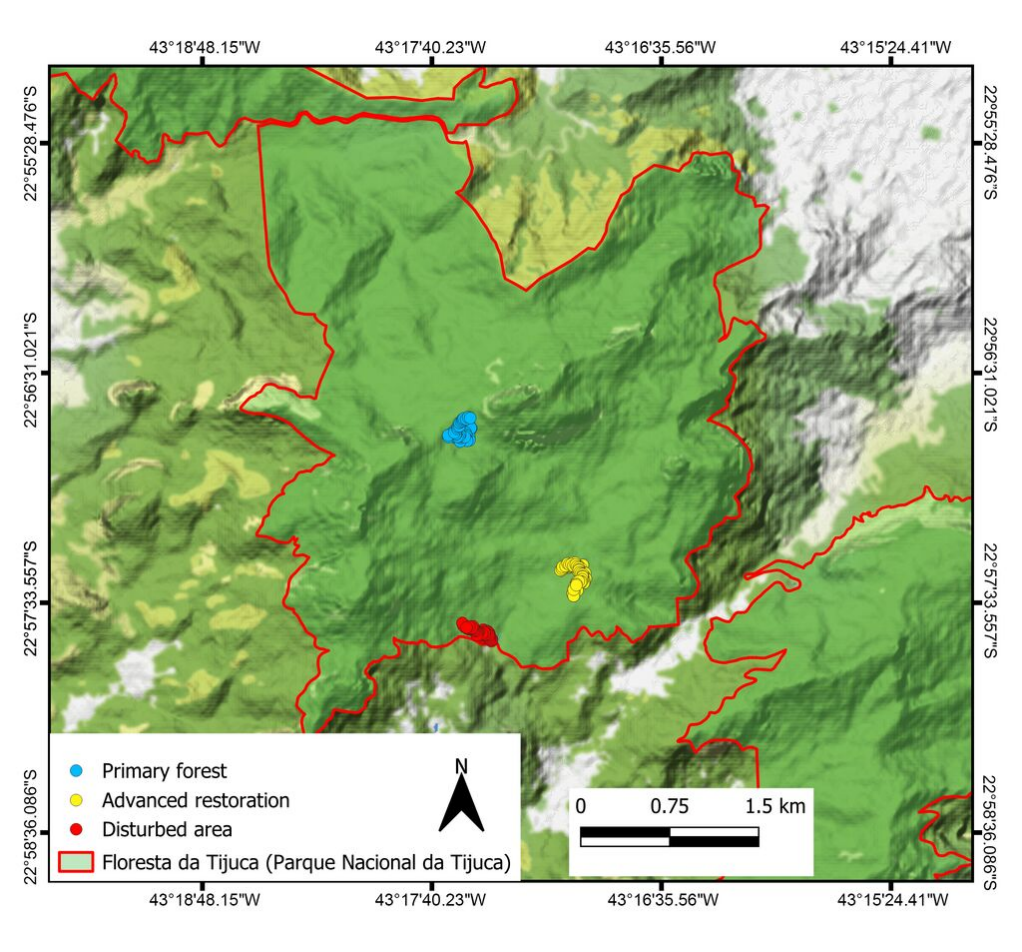
Map of the Floresta da Tijuca sector, Parque Nacional da Tijuca, showing the sampling localities.

Three regions in the Floresta da Tijuca sector of the PNT were selected (Fig. 1). According to the PNT management plan (Ministério do Meio Ambiente 2008), those regions are currently in three different levels of preservation, being classified as primary forest (PF), advanced restoration (ARS) and disturbed (DTBD). In each of them, two 180 m transects were established, with a distance of 50 m between them and an interval of 20 m between individual sampling points, totalling 240 samples. Collection was performed under SISBIO permit 78112-1, during the dry season (July 2021) and the rainy season (January 2022), using two techniques in each transect — 20 pitfall traps placed for 48 hours in the field and 20 samples of 1 m² of litter sorted using a Winkler extractor for 48 hours. These techniques complement each other, as they can sample the diversity of soil ants more representatively when combined (Agosti et al. 2000, Lopes et al. 2010, Orsolon-Souza et al. 2011 and Baccaro et al. 2015). The specimens collected were preserved in 70% ethanol until they were dry mounted, identified and deposited in the Coleção Entomológica Instituto Oswaldo Cruz, Fundação Oswaldo Cruz, Rio de Janeiro, Brazil.

Specimen sorting and identification were performed under a Zeiss Stemi SV6 stereomicroscope by following Kempf (1962), Kempf (1966), Brandão (1990), Lattke (1997), LaPolla (2004), Longino and Fernández (2007), Wild (2007), Galvis and Fenández (2009), Mackay and Mackay (2010), Sosa-Calvo et al. (2010), Longino (2013), Baccaro et al. (2015), Borowiec (2016), Ješovnik and Schultz (2017), Feitosa and Prada-Achiardi (2019), Fernández et al. (2019), Ortiz-Sepulveda et al. (2019), Camacho et al. (2020), Forti et al. (2022) and Probst and Brandão (2022) The geographic distribution listed for each represented species is according to the Global Ant Biodiversity Informatics (GABI) (Janicki et al. 2016 and Guénard 2017) and the ATLANTIC ANTS (Silva et al. 2022) datasets

## Checklists

### Ants from the Floresta da Tijuca sector, Parque Nacional da Tijuca

#### Formicidae

Latreille, 1809

1EB7B890-2E98-5A38-809D-66CDE36AA6B4

#### Amblyoponinae

Forel, 1893

C074A7F6-3659-5D55-8144-E0DB808FFB81

#### Fulakora elongata

(Santschi, 1912)

18114802-4018-5242-A73C-1FE6DD93747D

##### Materials

- **Type status:** Other material. **Occurrence:** recordNumber: WCC117; recordedBy: M. M. Silva, A. J. Mayhé Nunes, J. C. Santos, I. R. S. Cordeiro; individualCount: 1; sex: female; lifeStage: adult; occurrenceID: 002D4458-4529-5D74-B603-8A8951099BA2; **Taxon:** genus: Fulakora; specificEpithet: elongata; taxonRank: species; scientificNameAuthorship: (Santschi, 1912); **Location:** continent: South America; country: Brazil; countryCode: BR; stateProvince: Rio de Janeiro; municipality: Rio de Janeiro; locality: Floresta da Tijuca, Parque Nacional da Tijuca, Trilha de Moutain bike (Açude da solidão).; verbatimCoordinates: 22 57 40.0S 43 17 28.1 W; verbatimLatitude: 22 57 40.0S; verbatimLongitude: 43 17 28.1 W; verbatimCoordinateSystem: degrees minutes seconds; verbatimSRS: WGS84; **Identification:** identificationID: Fulakoraelongata; identifiedBy: M. M. Silva; **Event:** samplingProtocol: Winckler; year: 2022; month: 1; day: 21-23; verbatimEventDate: Summer 2022; habitat: Atlantic Forest; **Record Level:** type: PhysicalObject; institutionCode: CEIOC; basisOfRecord: PreservedSpecimen

##### Distribution

Argentina, Brazil, Paraguay, Uruguay.

##### Notes

This is the first record for Floresta da Tijuca sector. Previously recorded from the Serra da Carioca sector by Veiga-Ferreira et al. (2010).

#### Dolichoderinae

Forel, 1878

E7425C41-2959-5F9C-9C72-0496E3BCDB11

#### Linepithema angulatum

(Emery, 1894)

F7B509FC-E7EE-5B30-8804-5FB48EEC468F

##### Materials

- **Type status:** Other material. **Occurrence:** recordNumber: WBS115; recordedBy: M. M. Silva, A. J. Mayhé Nunes, J. C. Santos, I. R. S. Cordeiro; individualCount: 1; sex: female; lifeStage: adult; occurrenceID: D8D83C19-C430-52CB-BE21-8C1C34548598; **Taxon:** genus: Linepithema; specificEpithet: angulatum; taxonRank: species; scientificNameAuthorship: (Emery, 1894); **Location:** continent: South America; country: Brazil; countryCode: BR; stateProvince: Rio de Janeiro; municipality: Rio de Janeiro; locality: Floresta da Tijuca, Parque Nacional da Tijuca, trilha (caminho dos picos/ camminho da solidão); verbatimCoordinates: 22 57 29.3S 43 16 58.1W; verbatimLatitude: 22 57 29.3S; verbatimLongitude: 43 16 58.1W; verbatimCoordinateSystem: degrees minutes seconds; verbatimSRS: WGS84; **Identification:** identificationID: Linepithemaangulatum; identifiedBy: M. M. Silva; **Event:** samplingProtocol: Winckler; year: 2021; month: 7; day: 21-23; verbatimEventDate: Winter 2021; habitat: Atlantic Forest; **Record Level:** type: PhysicalObject; institutionCode: CEIOC; basisOfRecord: PreservedSpecimen

##### Distribution

Argentina, Bolivia, Brazil, Colombia, Costa Rica, Ecuador, Panama, Peru, Venezuela. Introduced to northern Europe.

##### Notes

This is the first record for PNT. Previously recorded from Atlantic Forest fragments in the State of Rio de Janeiro by Gomes et al. (2013).

#### Linepithema micans

(Forel, 1908)

7D78A315-7650-5F1B-B7A4-1EB496E6807D

##### Materials

- **Type status:** Other material. **Occurrence:** recordNumber: PAC110; recordedBy: M. M. Silva, A. J. Mayhé Nunes, J. C. Santos, I. R. S. Cordeiro; individualCount: 1; sex: female; lifeStage: adult; occurrenceID: F2D4D5FB-A5AE-5493-84B3-C5BC62275091; **Taxon:** genus: Linepithema; specificEpithet: micans; taxonRank: species; scientificNameAuthorship: (Forel, 1908); **Location:** continent: South America; country: Brazil; countryCode: BR; stateProvince: Rio de Janeiro; municipality: Rio de Janeiro; locality: Floresta da Tijuca, Parque Nacional da Tijuca, Bom Retiro (trilha para o pico da Tijuca); verbatimCoordinates: 22 56 48.3S 43 17 30.5W; verbatimLatitude: 22 56 48.3S; verbatimLongitude: 43 17 30.5W; verbatimCoordinateSystem: degrees minutes seconds; verbatimSRS: WGS84; **Identification:** identificationID: Linepithemamicans; identifiedBy: M. M. Silva; **Event:** samplingProtocol: Pitfall; year: 2022; month: 1; day: 17-19; verbatimEventDate: Summer 2022; habitat: Atlantic Forest; **Record Level:** type: PhysicalObject; institutionCode: CEIOC; basisOfRecord: PreservedSpecimen
- **Type status:** Other material. **Occurrence:** recordNumber: PAS119; recordedBy: M. M. Silva, A. J. Mayhé Nunes, J. C. Santos, I. R. S. Cordeiro; individualCount: 1; sex: female; lifeStage: adult; occurrenceID: 405FD860-4F2B-5CC2-B14F-F091E933E378; **Taxon:** genus: Linepithema; specificEpithet: micans; taxonRank: species; scientificNameAuthorship: (Forel, 1908); **Location:** continent: South America; country: Brazil; countryCode: BR; stateProvince: Rio de Janeiro; municipality: Rio de Janeiro; locality: Floresta da Tijuca, Parque Nacional da Tijuca, Bom Retiro (trilha para o pico da Tijuca); verbatimCoordinates: 22 56 04.1S 43 21 12.6W; verbatimLatitude: 22 56 04.1S; verbatimLongitude: 43 21 12.6W; verbatimCoordinateSystem: degrees minutes seconds; verbatimSRS: WGS84; **Identification:** identificationID: Linepithemamicans; identifiedBy: M. M. Silva; **Event:** samplingProtocol: Pitfall; year: 2021; month: 7; day: 19-21; verbatimEventDate: Winter 2021; habitat: Atlantic Forest; **Record Level:** type: PhysicalObject; institutionCode: CEIOC; basisOfRecord: PreservedSpecimen
- **Type status:** Other material. **Occurrence:** recordNumber: PBS112; recordedBy: M. M. Silva, A. J. Mayhé Nunes, J. C. Santos, I. R. S. Cordeiro; individualCount: 1; sex: female; lifeStage: adult; occurrenceID: FAD89592-F13D-5B6A-8157-0E3775F4B55B; **Taxon:** genus: Linepithema; specificEpithet: micans; taxonRank: species; scientificNameAuthorship: (Forel, 1908); **Location:** continent: South America; country: Brazil; countryCode: BR; stateProvince: Rio de Janeiro; municipality: Rio de Janeiro; locality: Floresta da Tijuca, Parque Nacional da Tijuca, trilha (caminho dos picos/ camminho da solidão); verbatimCoordinates: 22 57 26.2S 43 16 55.2W; verbatimLatitude: 22 57 26.2S; verbatimLongitude: 43 16 55.2W; verbatimCoordinateSystem: degrees minutes seconds; verbatimSRS: WGS84; **Identification:** identificationID: Linepithemamicans; identifiedBy: M. M. Silva; **Event:** samplingProtocol: Pitfall; year: 2021; month: 7; day: 21-23; verbatimEventDate: Winter 2021; habitat: Atlantic Forest; **Record Level:** type: PhysicalObject; institutionCode: CEIOC; basisOfRecord: PreservedSpecimen

##### Distribution

Argentina, Brazil, Costa Rica, Paraguay, Trinidad and Tobago, Uruguay. Exotic in Mexico.

##### Notes

Recorded from the PNT without details by Wild (2007).

#### Dorylinae

Leach, 1815

B59CC27A-542A-513D-AC1E-09A8032C3A45

#### Labidus coecus

(Latreille, 1802)

5332E803-7669-5BF8-921C-E970DA2A0B73

##### Materials

- **Type status:** Other material. **Occurrence:** recordNumber: PAC111; recordedBy: M. M. Silva, A. J. Mayhé Nunes, J. C. Santos, I. R. S. Cordeiro; individualCount: 1; sex: female; lifeStage: adult; occurrenceID: A43BB38C-8B4F-5C96-B09E-C5AA83C471AE; **Taxon:** genus: Labidus; specificEpithet: coecus; taxonRank: species; scientificNameAuthorship: (Latreille, 1802); **Location:** continent: South America; country: Brazil; countryCode: BR; stateProvince: Rio de Janeiro; municipality: Rio de Janeiro; locality: Floresta da Tijuca, Parque Nacional da Tijuca, Bom Retiro (trilha para o pico da Tijuca); verbatimCoordinates: 22 56 48.2S 43 17 30.0W; verbatimLatitude: 22 56 48.2S; verbatimLongitude: 43 17 30.0W; verbatimCoordinateSystem: degrees minutes seconds; verbatimSRS: WGS84; **Identification:** identificationID: Labiduscoecus; identifiedBy: M. M. Silva; **Event:** samplingProtocol: Pitfall; year: 2022; month: 1; day: 17-19; verbatimEventDate: Summer 2022; habitat: Atlantic Forest; **Record Level:** type: PhysicalObject; institutionCode: CEIOC; basisOfRecord: PreservedSpecimen
- **Type status:** Other material. **Occurrence:** recordNumber: PAC115; recordedBy: M. M. Silva, A. J. Mayhé Nunes, J. C. Santos, I. R. S. Cordeiro; individualCount: 1; sex: female; lifeStage: adult; occurrenceID: 463F7413-CB51-5793-BC9C-F3B726ECF0C4; **Taxon:** genus: Labidus; specificEpithet: coecus; taxonRank: species; scientificNameAuthorship: (Latreille, 1802); **Location:** continent: South America; country: Brazil; countryCode: BR; stateProvince: Rio de Janeiro; municipality: Rio de Janeiro; locality: Floresta da Tijuca, Parque Nacional da Tijuca, Bom Retiro (trilha para o pico da Tijuca); verbatimCoordinates: 22 56 49.5S 43 17 29.8W; verbatimLatitude: 23 56 49.5S; verbatimLongitude: 43 17 29.8W; verbatimCoordinateSystem: degrees minutes seconds; verbatimSRS: WGS84; **Identification:** identificationID: Labiduscoecus; identifiedBy: M. M. Silva; **Event:** samplingProtocol: Pitfall; year: 2022; month: 1; day: 17-19; verbatimEventDate: Summer 2022; habitat: Atlantic Forest; **Record Level:** type: PhysicalObject; institutionCode: CEIOC; basisOfRecord: PreservedSpecimen

##### Distribution

Argentina, Belize, Bolivia, Brazil, Colombia, Costa Rica, Ecuador, El Salvador, French Guiana, Guatemala, Guyana, Honduras, Lesser Antilles, Mexico, Nicaragua, Panama, Paraguay, Peru, Suriname, Trinidad and Tobago, United States, Uruguay, Venezuela.

##### Notes

This is the first record for the PNT. Recorded from the State of Rio de Janeiro without details by Kempf (1972) and Watkins (1976).

#### Labidus praedator

(Smith, F., 1858)

7634AA0B-1E1C-5B2C-8307-C6A00A6047F1

##### Materials

- **Type status:** Other material. **Occurrence:** recordNumber: PCC104; recordedBy: M. M. Silva, A. J. Mayhé Nunes, J. C. Santos, I. R. S. Cordeiro; individualCount: 3; sex: female; lifeStage: adult; occurrenceID: CDEBD54B-78EE-5102-8E74-FBCA309109E4; **Taxon:** genus: Labidus; specificEpithet: praedator; taxonRank: species; scientificNameAuthorship: (Smith, F., 1858); **Location:** continent: South America; country: Brazil; countryCode: BR; stateProvince: Rio de Janeiro; municipality: Rio de Janeiro; locality: Floresta da Tijuca, Parque Nacional da Tijuca, Trilha de Moutain bike (Açude da solidão).; verbatimCoordinates: 22 57 42.2S 43 17 24.3W; verbatimLatitude: 22 57 42.2S; verbatimLongitude: 43 17 24.3W; verbatimCoordinateSystem: degrees minutes seconds; verbatimSRS: WGS84; **Identification:** identificationID: Labiduspraedator; identifiedBy: M. M. Silva; **Event:** samplingProtocol: Pitfall; year: 2022; month: 1; day: 21-23; verbatimEventDate: Summer 2022; habitat: Atlantic Forest; **Record Level:** type: PhysicalObject; institutionCode: CEIOC; basisOfRecord: PreservedSpecimen
- **Type status:** Other material. **Occurrence:** recordNumber: PCC116; recordedBy: M. M. Silva, A. J. Mayhé Nunes, J. C. Santos, I. R. S. Cordeiro; individualCount: 3; sex: female; lifeStage: adult; occurrenceID: E206997E-9144-5CDF-A61F-F4B4BA6F1D53; **Taxon:** genus: Labidus; specificEpithet: praedator; taxonRank: species; scientificNameAuthorship: (Smith, F., 1858); **Location:** continent: South America; country: Brazil; countryCode: BR; stateProvince: Rio de Janeiro; municipality: Rio de Janeiro; locality: Floresta da Tijuca, Parque Nacional da Tijuca, Trilha de Moutain bike (Açude da solidão).; verbatimCoordinates: 22 57 41.8S 43 17 25.1W; verbatimLatitude: 22 57 41.8S; verbatimLongitude: 43 17 25.1W; verbatimCoordinateSystem: degrees minutes seconds; verbatimSRS: WGS84; **Identification:** identificationID: Labiduspraedator; identifiedBy: M. M. Silva; **Event:** samplingProtocol: Pitfall; year: 2022; month: 1; day: 21-23; verbatimEventDate: Summer 2022; habitat: Atlantic Forest; **Record Level:** type: PhysicalObject; institutionCode: CEIOC; basisOfRecord: PreservedSpecimen

##### Distribution

Argentina, Belize, Bolivia, Brazil, Colombia, Costa Rica, Ecuador, El Salvador, French Guiana, Guatemala, Guyana, Honduras, Mexico, Nicaragua, Panama, Paraguay, Peru, Suriname, United States, Venezuela.

##### Notes

This is the first record for PNT. Previously recorded from the State of Rio de Janeiro without details by Kempf (1972) and Watkins (1976).

#### Neocerapachys neotropicus

Weber, 1939

66BEDB36-A951-5E46-AFEA-8D5FF9FB8C15

##### Materials

- **Type status:** Other material. **Occurrence:** recordNumber: WCC107; recordedBy: M. M. Silva, A. J. Mayhé Nunes, J. C. Santos, I. R. S. Cordeiro; individualCount: 1; sex: female; lifeStage: adult; occurrenceID: A5E23B86-F721-5122-84EA-57D855A67249; **Taxon:** genus: Neocerapachys; specificEpithet: neotropicus; taxonRank: species; scientificNameAuthorship: Weber, 1939; **Location:** continent: South America; country: Brazil; countryCode: BR; stateProvince: Rio de Janeiro; municipality: Rio de Janeiro; locality: Floresta da Tijuca, Parque Nacional da Tijuca, Trilha de Moutain bike (Açude da solidão).; verbatimCoordinates: 22 57 42.2S 43 17 24.5W; verbatimLatitude: 22 57 42.2S; verbatimLongitude: 43 17 24.5W; verbatimCoordinateSystem: degrees minutes seconds; verbatimSRS: WGS84; **Identification:** identificationID: Neocerapachysneotropicus; identifiedBy: M. M. Silva; **Event:** samplingProtocol: Winckler; year: 2022; month: 1; day: 21-23; verbatimEventDate: Summer 2022; habitat: Atlantic Forest; **Record Level:** type: PhysicalObject; institutionCode: CEIOC; basisOfRecord: PreservedSpecimen

##### Distribution

Brazil, Colombia, Costa Rica, Ecuador, Lesser Antilles, French Guiana, Peru, Trinidad and Tobago, Venezuela.

##### Notes

This is the southernmost record of this species and also the first from the Atlantic Forest biome.

#### Ectatomminae

Emery, 1895

48021372-025A-51DB-AE36-863615C0B518

#### Ectatomma edentatum

Roger, 1863

91D9E7C7-D8E5-5F8E-A744-9BFEAA5A9D15

##### Materials

- **Type status:** Other material. **Occurrence:** recordNumber: PBC102; recordedBy: M. M. Silva, A. J. Mayhé Nunes, J. C. Santos, I. R. S. Cordeiro; individualCount: 1; sex: female; lifeStage: adult; occurrenceID: 70D094B0-827C-5C40-931F-4AE2B1209DAF; **Taxon:** genus: Ectatomma; specificEpithet: edentatum; taxonRank: species; scientificNameAuthorship: Roger, 1863; **Location:** continent: South America; country: Brazil; countryCode: BR; stateProvince: Rio de Janeiro; municipality: Rio de Janeiro; locality: Floresta da Tijuca, Parque Nacional da Tijuca, trilha (caminho dos picos/ camminho da solidão); verbatimCoordinates: 22 57 22.6S 43 16 58.5W; verbatimLatitude: 22 57 22.6S; verbatimLongitude: 43 16 58.5W; verbatimCoordinateSystem: degrees minutes seconds; verbatimSRS: WGS84; **Identification:** identificationID: Ectatommaedentatum; identifiedBy: M. M. Silva; **Event:** samplingProtocol: Pitfall; year: 2022; month: 1; day: 17-21; verbatimEventDate: Summer 2022; habitat: Atlantic Forest; **Record Level:** type: PhysicalObject; institutionCode: CEIOC; basisOfRecord: PreservedSpecimen
- **Type status:** Other material. **Occurrence:** recordNumber: PBC119; recordedBy: M. M. Silva, A. J. Mayhé Nunes, J. C. Santos, I. R. S. Cordeiro; individualCount: 1; sex: female; lifeStage: adult; occurrenceID: 3F72E7B1-81A9-5AA9-B3B7-E116D269893A; **Taxon:** genus: Ectatomma; specificEpithet: edentatum; taxonRank: species; scientificNameAuthorship: Roger, 1863; **Location:** continent: South America; country: Brazil; countryCode: BR; stateProvince: Rio de Janeiro; municipality: Rio de Janeiro; locality: Floresta da Tijuca, Parque Nacional da Tijuca, trilha (caminho dos picos/ camminho da solidão); verbatimCoordinates: 22 57 23.8S 43 17 01.6W; verbatimLatitude: 22 57 23.8S; verbatimLongitude: 43 17 01.6W; verbatimCoordinateSystem: degrees minutes seconds; verbatimSRS: WGS84; **Identification:** identificationID: Ectatommaedentatum; identifiedBy: M. M. Silva; **Event:** samplingProtocol: Pitfall; year: 2022; month: 1; day: 17-21; verbatimEventDate: Summer 2022; habitat: Atlantic Forest; **Record Level:** type: PhysicalObject; institutionCode: CEIOC; basisOfRecord: PreservedSpecimen
- **Type status:** Other material. **Occurrence:** recordNumber: PBC111; recordedBy: M. M. Silva, A. J. Mayhé Nunes, J. C. Santos, I. R. S. Cordeiro; individualCount: 1; sex: female; lifeStage: adult; occurrenceID: D9F3E944-159C-5296-AE61-85E5EB10DF53; **Taxon:** genus: Ectatomma; specificEpithet: edentatum; taxonRank: species; scientificNameAuthorship: Roger, 1863; **Location:** continent: South America; country: Brazil; countryCode: BR; stateProvince: Rio de Janeiro; municipality: Rio de Janeiro; locality: Floresta da Tijuca, Parque Nacional da Tijuca, trilha (caminho dos picos/ camminho da solidão); verbatimCoordinates: 22 57 23.5S 43 17 00.9W; verbatimLatitude: 22 57 23.5S; verbatimLongitude: 43 17 00.9W; verbatimCoordinateSystem: degrees minutes seconds; verbatimSRS: WGS84; **Identification:** identificationID: Ectatommaedentatum; identifiedBy: M. M. Silva; **Event:** samplingProtocol: Pitfall; year: 2022; month: 1; day: 17-21; verbatimEventDate: Summer 2022; habitat: Atlantic Forest; **Record Level:** type: PhysicalObject; institutionCode: CEIOC; basisOfRecord: PreservedSpecimen
- **Type status:** Other material. **Occurrence:** recordNumber: PBC110; recordedBy: M. M. Silva, A. J. Mayhé Nunes, J. C. Santos, I. R. S. Cordeiro; individualCount: 1; sex: female; lifeStage: adult; occurrenceID: D573356F-77D3-5BDD-B437-D4CBDC247E8F; **Taxon:** genus: Ectatomma; specificEpithet: edentatum; taxonRank: species; scientificNameAuthorship: Roger, 1863; **Location:** continent: South America; country: Brazil; countryCode: BR; stateProvince: Rio de Janeiro; municipality: Rio de Janeiro; locality: Floresta da Tijuca, Parque Nacional da Tijuca, trilha (caminho dos picos/ camminho da solidão); verbatimCoordinates: 22 57 23.8S 43 17 01.5W; verbatimLatitude: 22 57 23.8S; verbatimLongitude: 43 17 01.5W; verbatimCoordinateSystem: degrees minutes seconds; verbatimSRS: WGS84; **Identification:** identificationID: Ectatommaedentatum; identifiedBy: M. M. Silva; **Event:** samplingProtocol: Pitfall; year: 2022; month: 1; day: 17-21; verbatimEventDate: Summer 2022; habitat: Atlantic Forest; **Record Level:** type: PhysicalObject; institutionCode: CEIOC; basisOfRecord: PreservedSpecimen

##### Distribution

Argentina, Bolivia, Brazil, Colombia, Costa Rica, Ecuador, French Guiana, Guyana, Panama, Paraguay, Peru, Suriname, Trinidad and Tobago, Uruguay, Venezuela.

##### Notes

This is the first record for Floresta da Tijuca sector. Previously recorded from the Serra da Carioca sector by Vargas et al. (2013) and Santos et al. (2019).

#### Ectatomma muticum

Mayr, 1870

0E20BE5B-98E3-5328-83EB-F06FFE953A0B

##### Materials

- **Type status:** Other material. **Occurrence:** recordNumber: PBC120; recordedBy: M. M. Silva, A. J. Mayhé Nunes, J. C. Santos, I. R. S. Cordeiro; individualCount: 2; sex: female; lifeStage: adult; occurrenceID: 527E3A9A-2810-57C2-911A-18AF312CB192; **Taxon:** genus: Ectatomma; specificEpithet: muticum; taxonRank: species; scientificNameAuthorship: Mayr, 1870; **Location:** continent: South America; country: Brazil; countryCode: BR; stateProvince: Rio de Janeiro; municipality: Rio de Janeiro; locality: Floresta da Tijuca, Parque Nacional da Tijuca, trilha (caminho dos picos/ camminho da solidão); verbatimCoordinates: 22 57 23.6S 43 17 00.7W; verbatimLatitude: 22 57 23.6S; verbatimLongitude: 43 17 00.7W; verbatimCoordinateSystem: degrees minutes seconds; verbatimSRS: WGS84; **Identification:** identificationID: Ectatommamuticum; identifiedBy: M. M. Silva; **Event:** samplingProtocol: Pitfall; year: 2022; month: 1; day: 17-21; verbatimEventDate: Summer 2022; habitat: Atlantic Forest; **Record Level:** type: PhysicalObject; institutionCode: CEIOC; basisOfRecord: PreservedSpecimen
- **Type status:** Other material. **Occurrence:** recordNumber: WBC108; recordedBy: M. M. Silva, A. J. Mayhé Nunes, J. C. Santos, I. R. S. Cordeiro; individualCount: 1; sex: female; lifeStage: adult; occurrenceID: B81EBEED-468E-5CA6-9824-AAC60813DEAC; **Taxon:** genus: Ectatomma; specificEpithet: muticum; taxonRank: species; scientificNameAuthorship: Mayr, 1870; **Location:** continent: South America; country: Brazil; countryCode: BR; stateProvince: Rio de Janeiro; municipality: Rio de Janeiro; locality: Floresta da Tijuca, Parque Nacional da Tijuca, trilha (caminho dos picos/ camminho da solidão); verbatimCoordinates: 22 57 24.5S 43 16 56.7W; verbatimLatitude: 22 57 24.5S; verbatimLongitude: 43 16 56.7W; verbatimCoordinateSystem: degrees minutes seconds; verbatimSRS: WGS84; **Identification:** identificationID: Ectatommamuticum; identifiedBy: M. M. Silva; **Event:** samplingProtocol: Winckler; year: 2022; month: 1; day: 17-21; verbatimEventDate: Summer 2022; habitat: Atlantic Forest; **Record Level:** type: PhysicalObject; institutionCode: CEIOC; basisOfRecord: PreservedSpecimen

##### Distribution

Brazil, Colombia, Guyana, Paraguay. The record from Mexico is dubious.

##### Notes

This is the first record for State of Rio de Janeiro.

#### Ectatomma permagnum

Forel, 1908

C21C2A2E-2791-525B-8197-6DD68ADBAD09

##### Materials

- **Type status:** Other material. **Occurrence:** recordNumber: PBS103; recordedBy: M. M. Silva, A. J. Mayhé Nunes, J. C. Santos, I. R. S. Cordeiro; individualCount: 1; sex: female; lifeStage: adult; occurrenceID: 4CBD3F9B-4C1B-58A3-857B-8A8039FBFE20; **Taxon:** genus: Ectatomma; specificEpithet: permagnum; taxonRank: species; scientificNameAuthorship: Forel, 1908; **Location:** continent: South America; country: Brazil; countryCode: BR; stateProvince: Rio de Janeiro; municipality: Rio de Janeiro; locality: Floresta da Tijuca, Parque Nacional da Tijuca, trilha (caminho dos picos/ camminho da solidão); verbatimCoordinates: 22 57 23.0S 43 16 58.5W; verbatimLatitude: 22 57 23.0S; verbatimLongitude: 43 16 58.5W; verbatimCoordinateSystem: degrees minutes seconds; verbatimSRS: WGS84; **Identification:** identificationID: Ectatommapermagnum; identifiedBy: M. M. Silva; **Event:** samplingProtocol: Pitfall; year: 2021; month: 7; day: 21-23; verbatimEventDate: Winter 2021; habitat: Atlantic Forest; **Record Level:** type: PhysicalObject; institutionCode: CEIOC; basisOfRecord: PreservedSpecimen

##### Distribution

Argentina, Bolivia, Brazil, Colombia, Paraguay. The record from Guyana is dubious.

##### Notes

This is the first record for the PNT. Kempf (1972) recorded this species from the City of Rio de Janeiro without details.

#### Gnamptogenys continua

(Mayr, 1887)

3BDBB752-D74F-597D-8C1B-008B2A5F165D

##### Materials

- **Type status:** Other material. **Occurrence:** recordNumber: WAC110; recordedBy: M. M. Silva, A. J. Mayhé Nunes, J. C. Santos, I. R. S. Cordeiro; individualCount: 3; sex: female; lifeStage: adult; occurrenceID: AED2CC12-CC4C-5FFA-9306-17F1818A3086; **Taxon:** genus: Gnamptogenys; specificEpithet: continua; taxonRank: species; scientificNameAuthorship: (Mayr, 1887); **Location:** continent: South America; country: Brazil; countryCode: BR; stateProvince: Rio de Janeiro; municipality: Rio de Janeiro; locality: Floresta da Tijuca, Parque Nacional da Tijuca, Bom Retiro (trilha para o pico da Tijuca); verbatimCoordinates: 22 56 46.2S 43 17 32.7W; verbatimLatitude: 22 56 46.2S; verbatimLongitude: 43 17 32.7W; verbatimCoordinateSystem: degrees minutes seconds; verbatimSRS: WGS84; **Identification:** identificationID: Gnamptogenyscontinua; identifiedBy: M. M. Silva; **Event:** samplingProtocol: Winckler; year: 2022; month: 1; day: 17-19; verbatimEventDate: Summer 2022; habitat: Atlantic Forest; **Record Level:** type: PhysicalObject; institutionCode: CEIOC; basisOfRecord: PreservedSpecimen
- **Type status:** Other material. **Occurrence:** recordNumber: WBC115; recordedBy: M. M. Silva, A. J. Mayhé Nunes, J. C. Santos, I. R. S. Cordeiro; individualCount: 2; sex: female; lifeStage: adult; occurrenceID: 3DF7483E-CAED-551F-BB76-9D03D1B22B89; **Taxon:** genus: Gnamptogenys; specificEpithet: continua; taxonRank: species; scientificNameAuthorship: (Mayr, 1887); **Location:** continent: South America; country: Brazil; countryCode: BR; stateProvince: Rio de Janeiro; municipality: Rio de Janeiro; locality: Floresta da Tijuca, Parque Nacional da Tijuca, Bom Retiro (trilha para o pico da Tijuca); verbatimCoordinates: 22 57 27.2S 43 16 55.8W; verbatimLatitude: 22 57 27.2S; verbatimLongitude: 43 16 55.8W; verbatimCoordinateSystem: degrees minutes seconds; verbatimSRS: WGS84; **Identification:** identificationID: Gnamptogenyscontinua; identifiedBy: M. M. Silva; **Event:** samplingProtocol: Winckler; year: 2022; month: 1; day: 19-21; verbatimEventDate: Summer 2022; habitat: Atlantic Forest; **Record Level:** type: PhysicalObject; institutionCode: CEIOC; basisOfRecord: PreservedSpecimen
- **Type status:** Other material. **Occurrence:** recordNumber: WCC112; recordedBy: M. M. Silva, A. J. Mayhé Nunes, J. C. Santos, I. R. S. Cordeiro; individualCount: 2; sex: female; lifeStage: adult; occurrenceID: A647FEEB-3DDA-5A3F-82B8-CD90766798F6; **Taxon:** genus: Gnamptogenys; specificEpithet: continua; taxonRank: species; scientificNameAuthorship: (Mayr, 1887); **Location:** continent: South America; country: Brazil; countryCode: BR; stateProvince: Rio de Janeiro; municipality: Rio de Janeiro; locality: Floresta da Tijuca, Parque Nacional da Tijuca, Trilha de Moutain bike (Açude da solidão).; verbatimCoordinates: 22 57 42.4S 43 17 26.9W; verbatimLatitude: 22 57 42.4S; verbatimLongitude: 43 17 26.9W; verbatimCoordinateSystem: degrees minutes seconds; verbatimSRS: WGS84; **Identification:** identificationID: Gnamptogenyscontinua; identifiedBy: M. M. Silva; **Event:** samplingProtocol: Winckler; year: 2022; month: 1; day: 21-23; verbatimEventDate: Summer 2022; habitat: Atlantic Forest; **Record Level:** type: PhysicalObject; institutionCode: CEIOC; basisOfRecord: PreservedSpecimen

##### Distribution

Belize, Bolivia, Brazil, Colombia, Costa Rica, Ecuador, French Guiana, Guyana, Honduras, Jamaica, Mexico, Panama, Paraguay, Peru, Suriname, Venezuela.

##### Notes

This is the first record for the PNT. Silva and Brandão (2014) recorded this species from other protected areas in the State of Rio de Janeiro.

#### Gnamptogenys horni

(Santschi, 1929)

4DE445F6-E142-5929-9217-12137C9E81BC

##### Materials

- **Type status:** Other material. **Occurrence:** recordNumber: WAC104; recordedBy: M. M. Silva, A. J. Mayhé Nunes, J. C. Santos, I. R. S. Cordeiro; individualCount: 1; sex: female; lifeStage: adult; occurrenceID: 52805C1E-270C-5B30-A77D-2DE474F105A5; **Taxon:** genus: Gnamptogenys; specificEpithet: horni; taxonRank: species; scientificNameAuthorship: (Santschi, 1929); **Location:** continent: South America; country: Brazil; countryCode: BR; stateProvince: Rio de Janeiro; municipality: Rio de Janeiro; locality: Floresta da Tijuca, Parque Nacional da Tijuca, Bom Retiro (trilha para o pico da Tijuca); verbatimCoordinates: 22 56 48.4S 43 17 32.7W; verbatimLatitude: 22 56 48.4S; verbatimLongitude: 43 17 32.7W; verbatimCoordinateSystem: degrees minutes seconds; verbatimSRS: WGS84; **Identification:** identificationID: Gnamptogenyshorni; identifiedBy: M. M. Silva; **Event:** samplingProtocol: Winckler; year: 2022; month: 1; day: 17-19; verbatimEventDate: Summer 2022; habitat: Atlantic Forest; **Record Level:** type: PhysicalObject; institutionCode: CEIOC; basisOfRecord: PreservedSpecimen
- **Type status:** Other material. **Occurrence:** recordNumber: WCC102; recordedBy: M. M. Silva, A. J. Mayhé Nunes, J. C. Santos, I. R. S. Cordeiro; individualCount: 1; sex: female; lifeStage: adult; occurrenceID: 4667ED7B-E343-50A2-844D-2EE3C07559B9; **Taxon:** genus: Gnamptogenys; specificEpithet: horni; taxonRank: species; scientificNameAuthorship: (Santschi, 1929); **Location:** continent: South America; country: Brazil; countryCode: BR; stateProvince: Rio de Janeiro; municipality: Rio de Janeiro; locality: Floresta da Tijuca, Parque Nacional da Tijuca, Trilha de Moutain bike (Açude da solidão).; verbatimCoordinates: 22 57 43.6S 43 17 23.0W; verbatimLatitude: 22 57 43.6S; verbatimLongitude: 43 17 23.0W; verbatimCoordinateSystem: degrees minutes seconds; verbatimSRS: WGS84; **Identification:** identificationID: Gnamptogenyshorni; identifiedBy: M. M. Silva; **Event:** samplingProtocol: Winckler; year: 2022; month: 1; day: 21-23; verbatimEventDate: Summer 2022; habitat: Atlantic Forest; **Record Level:** type: PhysicalObject; institutionCode: CEIOC; basisOfRecord: PreservedSpecimen

##### Distribution

Argentina, Bolivia, Brazil, Colombia, Costa Rica, Ecuador, French Guiana, Guyana, Lesser Antilles, Panama, Peru, Suriname, Trinidad and Tobago, Venezuela.

##### Notes

This is the first record for the PNT. Previously recorded by Orsolon-Souza et al. (2011) and Silva and Brandão (2014) from other protected areas in the State of Rio de Janeiro.

#### Heteroponera dentinodis

(Mayr, 1887)

103E9774-2074-5C03-8EBD-F8F3A0459B09

##### Materials

- **Type status:** Other material. **Occurrence:** recordNumber: WAC110; recordedBy: M. M. Silva, A. J. Mayhé Nunes, J. C. Santos, I. R. S. Cordeiro; individualCount: 1; sex: female; lifeStage: adult; occurrenceID: 6C46D4DC-8D9C-5991-BBF3-3AAC5F3A301C; **Taxon:** genus: Heteroponera; specificEpithet: dentinodis; taxonRank: species; scientificNameAuthorship: (Mayr, 1887); **Location:** continent: South America; country: Brazil; countryCode: BR; stateProvince: Rio de Janeiro; municipality: Rio de Janeiro; locality: Floresta da Tijuca, Parque Nacional da Tijuca, Bom Retiro (trilha para o pico da Tijuca); verbatimCoordinates: 22 56 46.2S 43 17 32.7W; verbatimLatitude: 22 56 46.2S; verbatimLongitude: 43 17 32.7W; verbatimCoordinateSystem: degrees minutes seconds; verbatimSRS: WGS84; **Identification:** identificationID: Heteroponeradentinodis; identifiedBy: M. M. Silva; **Event:** samplingProtocol: Winckler; year: 2022; month: 1; day: 17-19; verbatimEventDate: Summer 2022; habitat: Atlantic Forest; **Record Level:** type: PhysicalObject; institutionCode: CEIOC; basisOfRecord: PreservedSpecimen

##### Distribution

Bolivia, French Guiana, Brazil. The record from Costa Rica needs verification.

##### Notes

Previously recorded from "Floresta da Tijuca" by Feitosa (2011).

#### Heteroponera dolo

(Roger, 1860)

E2146B88-2471-50B7-9802-3D9DD23795A1

##### Materials

- **Type status:** Other material. **Occurrence:** recordNumber: WAC119; recordedBy: M. M. Silva, A. J. Mayhé Nunes, J. C. Santos, I. R. S. Cordeiro; individualCount: 8; sex: female; lifeStage: adult; occurrenceID: 42EBFD7A-8334-5056-8A96-9E033651BB73; **Taxon:** genus: Heteroponera; specificEpithet: dolo; taxonRank: species; scientificNameAuthorship: (Roger, 1860); **Location:** continent: South America; country: Brazil; countryCode: BR; stateProvince: Rio de Janeiro; municipality: Rio de Janeiro; locality: Floresta da Tijuca, Parque Nacional da Tijuca, Bom Retiro (trilha para o pico da Tijuca); verbatimCoordinates: 22 56 43.1S 43 17 29.5W; verbatimLatitude: 22 56 43.1S; verbatimLongitude: 43 17 29.5W; verbatimCoordinateSystem: degrees minutes seconds; verbatimSRS: WGS84; **Identification:** identificationID: Heteroponeradolo; identifiedBy: M. M. Silva; **Event:** samplingProtocol: Winckler; year: 2022; month: 1; day: 17-19; verbatimEventDate: Summer 2022; habitat: Atlantic Forest; **Record Level:** type: PhysicalObject; institutionCode: CEIOC; basisOfRecord: PreservedSpecimen
- **Type status:** Other material. **Occurrence:** recordNumber: WCS119; recordedBy: M. M. Silva, A. J. Mayhé Nunes, J. C. Santos, I. R. S. Cordeiro; individualCount: 1; sex: female; lifeStage: adult; occurrenceID: 4E819A45-2214-5577-8AE4-9BEAC7C2D9A8; **Taxon:** genus: Heteroponera; specificEpithet: dolo; taxonRank: species; scientificNameAuthorship: (Roger, 1860); **Location:** continent: South America; country: Brazil; countryCode: BR; stateProvince: Rio de Janeiro; municipality: Rio de Janeiro; locality: Floresta da Tijuca, Parque Nacional da Tijuca, Trilha de Moutain bike (Açude da solidão).; verbatimCoordinates: 22 57 39.8S 43 17 30.9W; verbatimLatitude: 22 57 39.8S; verbatimLongitude: 43 17 30.9W; verbatimCoordinateSystem: degrees minutes seconds; verbatimSRS: WGS84; **Identification:** identificationID: Heteroponeradolo; identifiedBy: M. M. Silva; **Event:** samplingProtocol: Winckler; year: 2021; month: 7; day: 23-25; verbatimEventDate: Winter 2021; habitat: Atlantic Forest; **Record Level:** type: PhysicalObject; institutionCode: CEIOC; basisOfRecord: PreservedSpecimen

##### Distribution

Argentina, Brazil, Colombia, Paraguay, Uruguay.

##### Notes

This is the first record for the PNT. Kempf (1972) recorded this species from the City of Rio de Janeiro without details.

#### Heteroponera mayri

Kempf, 1962

4A3F7CB3-2382-5E52-A99B-45BE8CAE0E7D

##### Materials

- **Type status:** Other material. **Occurrence:** recordNumber: WAS102; recordedBy: M. M. Silva, A. J. Mayhé Nunes, J. C. Santos, I. R. S. Cordeiro; individualCount: 2; sex: female; lifeStage: adult; occurrenceID: 58D6CF36-4FEF-5083-A197-7AAC007D72E1; **Taxon:** genus: Heteroponera; specificEpithet: mayri; taxonRank: species; scientificNameAuthorship: Kempf, 1962; **Location:** continent: South America; country: Brazil; countryCode: BR; stateProvince: Rio de Janeiro; municipality: Rio de Janeiro; locality: Floresta da Tijuca, Parque Nacional da Tijuca, Bom Retiro (trilha para o pico da Tijuca); verbatimCoordinates: 22 56 47.5S 43 17 30.6W; verbatimLatitude: 22 56 47.5S; verbatimLongitude: 43 17 30.6W; verbatimCoordinateSystem: degrees minutes seconds; verbatimSRS: WGS84; **Identification:** identificationID: Heteroponeramayri; identifiedBy: M. M. Silva; **Event:** samplingProtocol: Winckler; year: 2021; month: 7; day: 19-21; verbatimEventDate: Winter 2021; habitat: Atlantic Forest; **Record Level:** type: PhysicalObject; institutionCode: CEIOC; basisOfRecord: PreservedSpecimen
- **Type status:** Other material. **Occurrence:** recordNumber: WBS105; recordedBy: M. M. Silva, A. J. Mayhé Nunes, J. C. Santos, I. R. S. Cordeiro; individualCount: 1; sex: female; lifeStage: adult; occurrenceID: 32C3A15C-1CE7-5A82-9714-987E97CBDBE9; **Taxon:** genus: Heteroponera; specificEpithet: mayri; taxonRank: species; scientificNameAuthorship: Kempf, 1962; **Location:** continent: South America; country: Brazil; countryCode: BR; stateProvince: Rio de Janeiro; municipality: Rio de Janeiro; locality: Floresta da Tijuca, Parque Nacional da Tijuca, trilha (caminho dos picos/ camminho da solidão); verbatimCoordinates: 22 57 23.8S 43 16 57.7W; verbatimLatitude: 22 57 23.8S; verbatimLongitude: 43 16 57.7W; verbatimCoordinateSystem: degrees minutes seconds; verbatimSRS: WGS84; **Identification:** identificationID: Heteroponeramayri; identifiedBy: M. M. Silva; **Event:** samplingProtocol: Winckler; year: 2021; month: 7; day: 21-23; verbatimEventDate: Winter 2021; habitat: Atlantic Forest; **Record Level:** type: PhysicalObject; institutionCode: CEIOC; basisOfRecord: PreservedSpecimen

##### Distribution

Argentina, Brazil, Paraguay, Puerto Rico. Records from Colombia and Mexico need verification.

##### Notes

This is the first record for the PNT. Silva and Brandão (2014) recorded this species from other protected areas in the State of Rio de Janeiro.

#### Holcoponera mina

Brown, 1956

B8261D20-A34C-58D1-86DA-FC5F16E58758

##### Materials

- **Type status:** Other material. **Occurrence:** recordNumber: WAS118; recordedBy: M. M. Silva, A. J. Mayhé Nunes, J. C. Santos, I. R. S. Cordeiro; individualCount: 2; sex: female; lifeStage: adult; occurrenceID: 72E1DF36-C242-529F-8C2C-B488922A965A; **Taxon:** genus: Holcoponera; specificEpithet: mina; taxonRank: species; scientificNameAuthorship: Brown, 1956; **Location:** continent: South America; country: Brazil; countryCode: BR; stateProvince: Rio de Janeiro; municipality: Rio de Janeiro; locality: Floresta da Tijuca, Parque Nacional da Tijuca, Bom Retiro (trilha para o pico da Tijuca); verbatimCoordinates: 22 56 45.6S 43 17 32.5W; verbatimLatitude: 22 56 45.6S; verbatimLongitude: 43 17 32.5W; verbatimCoordinateSystem: degrees minutes seconds; verbatimSRS: WGS84; **Identification:** identificationID: Holcoponeramina; identifiedBy: M. M. Silva; **Event:** samplingProtocol: Winckler; year: 2021; month: 7; day: 19-21; verbatimEventDate: Winter 2021; habitat: Atlantic Forest; **Record Level:** type: PhysicalObject; institutionCode: CEIOC; basisOfRecord: PreservedSpecimen

##### Distribution

Bolivia, Brazil, Colombia, Ecuador, French Guiana, Guyana, Peru, Venezuela. The record from Mexico needs verification.

##### Notes

This is the first record for the State of Rio de Janeiro, which expands the known range southwards.

#### Holcoponera moelleri

Forel, 1912

04A31853-E728-5506-A07A-81498F5EDAE1

##### Materials

- **Type status:** Other material. **Occurrence:** recordNumber: PAS105; recordedBy: M. M. Silva, A. J. Mayhé Nunes, J. C. Santos, I. R. S. Cordeiro; individualCount: 8; sex: female; lifeStage: adult; occurrenceID: 674B4090-C7F0-5506-AF90-BE16201B4760; **Taxon:** genus: Holcoponera; specificEpithet: moelleri; taxonRank: species; scientificNameAuthorship: Forel, 1912; **Location:** continent: South America; country: Brazil; countryCode: BR; stateProvince: Rio de Janeiro; municipality: Rio de Janeiro; locality: Floresta da Tijuca, Parque Nacional da Tijuca, Bom Retiro (trilha para o pico da Tijuca); verbatimCoordinates: 22 56 46.0S 43 17 29.1W; verbatimLatitude: 22 56 46.0S; verbatimLongitude: 43 17 29.1W; verbatimCoordinateSystem: degrees minutes seconds; verbatimSRS: WGS84; **Identification:** identificationID: Holcoponeramoelleri; identifiedBy: M. M. Silva; **Event:** samplingProtocol: Pitfall; year: 2021; month: 7; day: 19-21; verbatimEventDate: Winter 2021; habitat: Atlantic Forest; **Record Level:** type: PhysicalObject; institutionCode: CEIOC; basisOfRecord: PreservedSpecimen
- **Type status:** Other material. **Occurrence:** recordNumber: WAS108; recordedBy: M. M. Silva, A. J. Mayhé Nunes, J. C. Santos, I. R. S. Cordeiro; individualCount: 6; sex: female; lifeStage: adult; occurrenceID: 943CC490-7448-54C1-B5A4-3FA422DC3DBC; **Taxon:** genus: Holcoponera; specificEpithet: moelleri; taxonRank: species; scientificNameAuthorship: Forel, 1912; **Location:** continent: South America; country: Brazil; countryCode: BR; stateProvince: Rio de Janeiro; municipality: Rio de Janeiro; locality: Floresta da Tijuca, Parque Nacional da Tijuca, Bom Retiro (trilha para o pico da Tijuca); verbatimCoordinates: 22 56 47.0S 43 17 31.3W; verbatimLatitude: 22 56 47.0S; verbatimLongitude: 43 17 31.3W; verbatimCoordinateSystem: degrees minutes seconds; verbatimSRS: WGS84; **Identification:** identificationID: Holcoponeramoelleri; identifiedBy: M. M. Silva; **Event:** samplingProtocol: Winckler; year: 2021; month: 7; day: 19-21; verbatimEventDate: Winter 2021; habitat: Atlantic Forest; **Record Level:** type: PhysicalObject; institutionCode: CEIOC; basisOfRecord: PreservedSpecimen
- **Type status:** Other material. **Occurrence:** recordNumber: PBC104; recordedBy: M. M. Silva, A. J. Mayhé Nunes, J. C. Santos, I. R. S. Cordeiro; individualCount: 11; sex: female; lifeStage: adult; occurrenceID: 29D1E83A-EDBE-5619-A401-92C3A95EEF90; **Taxon:** genus: Holcoponera; specificEpithet: moelleri; taxonRank: species; scientificNameAuthorship: Forel, 1912; **Location:** continent: South America; country: Brazil; countryCode: BR; stateProvince: Rio de Janeiro; municipality: Rio de Janeiro; locality: Floresta da Tijuca, Parque Nacional da Tijuca, trilha (caminho dos picos/ camminho da solidão); verbatimCoordinates: 22 57 22.7S 43 16 57.9W; verbatimLatitude: 22 57 22.7S; verbatimLongitude: 43 16 57.9W; verbatimCoordinateSystem: degrees minutes seconds; verbatimSRS: WGS84; **Identification:** identificationID: Holcoponeramoelleri; identifiedBy: M. M. Silva; **Event:** samplingProtocol: Pitfall; year: 2022; month: 1; day: 17-21; verbatimEventDate: Summer 2022; habitat: Atlantic Forest; **Record Level:** type: PhysicalObject; institutionCode: CEIOC; basisOfRecord: PreservedSpecimen
- **Type status:** Other material. **Occurrence:** recordNumber: WBC115; recordedBy: M. M. Silva, A. J. Mayhé Nunes, J. C. Santos, I. R. S. Cordeiro; individualCount: 15; sex: female; lifeStage: adult; occurrenceID: E99CF9C3-7662-5C28-B017-16CEAB827093; **Taxon:** genus: Holcoponera; specificEpithet: moelleri; taxonRank: species; scientificNameAuthorship: Forel, 1912; **Location:** continent: South America; country: Brazil; countryCode: BR; stateProvince: Rio de Janeiro; municipality: Rio de Janeiro; locality: Floresta da Tijuca, Parque Nacional da Tijuca, trilha (caminho dos picos/ camminho da solidão); verbatimCoordinates: 22 57 27.2S 43 16 55.8W; verbatimLatitude: 22 57 27.2S; verbatimLongitude: 43 16 55.8W; verbatimCoordinateSystem: degrees minutes seconds; verbatimSRS: WGS84; **Identification:** identificationID: Holcoponeramoelleri; identifiedBy: M. M. Silva; **Event:** samplingProtocol: Winckler; year: 2022; month: 1; day: 17-22; verbatimEventDate: Summer 2022; habitat: Atlantic Forest; **Record Level:** type: PhysicalObject; institutionCode: CEIOC; basisOfRecord: PreservedSpecimen
- **Type status:** Other material. **Occurrence:** recordNumber: PCC111; recordedBy: M. M. Silva, A. J. Mayhé Nunes, J. C. Santos, I. R. S. Cordeiro; individualCount: 9; sex: female; lifeStage: adult; occurrenceID: 29BD3AC6-FA84-5D6E-BF45-139102FD746A; **Taxon:** genus: Holcoponera; specificEpithet: moelleri; taxonRank: species; scientificNameAuthorship: Forel, 1912; **Location:** continent: South America; country: Brazil; countryCode: BR; stateProvince: Rio de Janeiro; municipality: Rio de Janeiro; locality: Floresta da Tijuca, Parque Nacional da Tijuca, Trilha de Moutain bike (Açude da solidão).; verbatimCoordinates: 22 57 42.8S 43 17 23.5W; verbatimLatitude: 22 57 42.8S; verbatimLongitude: 43 17 23.5W; verbatimCoordinateSystem: degrees minutes seconds; verbatimSRS: WGS84; **Identification:** identificationID: Holcoponeramoelleri; identifiedBy: M. M. Silva; **Event:** samplingProtocol: Pitfall; year: 2022; month: 1; day: 21-23; verbatimEventDate: Summer 2022; habitat: Atlantic Forest; **Record Level:** type: PhysicalObject; institutionCode: CEIOC; basisOfRecord: PreservedSpecimen
- **Type status:** Other material. **Occurrence:** recordNumber: WCC106; recordedBy: M. M. Silva, A. J. Mayhé Nunes, J. C. Santos, I. R. S. Cordeiro; individualCount: 16; sex: female; lifeStage: adult; occurrenceID: 0199D8B1-3579-5F74-B688-F99E33C97DB9; **Taxon:** genus: Holcoponera; specificEpithet: moelleri; taxonRank: species; scientificNameAuthorship: Forel, 1912; **Location:** continent: South America; country: Brazil; countryCode: BR; stateProvince: Rio de Janeiro; municipality: Rio de Janeiro; locality: Floresta da Tijuca, Parque Nacional da Tijuca, Trilha de Moutain bike (Açude da solidão).; verbatimCoordinates: 22 57 42.2S 43 17 23.9W; verbatimLatitude: 22 57 42.2S; verbatimLongitude: 43 17 23.9W; verbatimCoordinateSystem: degrees minutes seconds; verbatimSRS: WGS84; **Identification:** identificationID: Holcoponeramoelleri; identifiedBy: M. M. Silva; **Event:** samplingProtocol: Winckler; year: 2022; month: 1; day: 21-23; verbatimEventDate: Summer 2022; habitat: Atlantic Forest; **Record Level:** type: PhysicalObject; institutionCode: CEIOC; basisOfRecord: PreservedSpecimen

##### Distribution

Argentina, Bolivia, Brazil, Colombia, Ecuador, French Guiana, Guyana, Paraguay, Peru, Suriname, Venezuela

##### Notes

This is the first record for the PNT. Teixeira et al. (2019) recorded this species from another protected area in the State of Rio de Janeiro.

#### Typhlomyrmex lavra

(Lattke, 2002)

69D03BC1-3B86-5F2C-9D09-807BAC7362AD

##### Materials

- **Type status:** Other material. **Occurrence:** recordNumber: WAS116; recordedBy: M. M. Silva, A. J. Mayhé Nunes, J. C. Santos, I. R. S. Cordeiro; individualCount: 1; sex: female; lifeStage: adult; occurrenceID: F19903AE-AF25-5BAA-95A0-942DC533B0BD; **Taxon:** genus: Typhlomyrmex; specificEpithet: lavra; taxonRank: species; scientificNameAuthorship: (Lattke, 2002); **Location:** continent: South America; country: Brazil; countryCode: BR; stateProvince: Rio de Janeiro; municipality: Rio de Janeiro; locality: Floresta da Tijuca, Parque Nacional da Tijuca, Bom Retiro (trilha para o pico da Tijuca); verbatimCoordinates: 22 56 46.8S 43 17 33.6W; verbatimLatitude: 22 56 46.8S; verbatimLongitude: 43 17 33.6W; verbatimCoordinateSystem: degrees minutes seconds; verbatimSRS: WGS84; **Identification:** identificationID: Typhlomyrmexlavra; identifiedBy: M. M. Silva; **Event:** samplingProtocol: Winckler; year: 2021; month: 7; day: 21-23; verbatimEventDate: Winter 2021; habitat: Atlantic Forest; **Record Level:** type: PhysicalObject; institutionCode: CEIOC; basisOfRecord: PreservedSpecimen
- **Type status:** Other material. **Occurrence:** recordNumber: WBS112; recordedBy: M. M. Silva, A. J. Mayhé Nunes, J. C. Santos, I. R. S. Cordeiro; individualCount: 2; sex: female; lifeStage: adult; occurrenceID: C3C1CB57-46B2-5341-9CA0-FCF0D60B421B; **Taxon:** genus: Typhlomyrmex; specificEpithet: lavra; taxonRank: species; scientificNameAuthorship: (Lattke, 2002); **Location:** continent: South America; country: Brazil; countryCode: BR; stateProvince: Rio de Janeiro; municipality: Rio de Janeiro; locality: Floresta da Tijuca, Parque Nacional da Tijuca, trilha (caminho dos picos/ camminho da solidão); verbatimCoordinates: 22 57 27.0S 43 16 55.4W; verbatimLatitude: 22 57 27.0S; verbatimLongitude: 43 16 55.4W; verbatimCoordinateSystem: degrees minutes seconds; verbatimSRS: WGS84; **Identification:** identificationID: Typhlomyrmexlavra; identifiedBy: M. M. Silva; **Event:** samplingProtocol: Winckler; year: 2021; month: 7; day: 19-21; verbatimEventDate: Winter 2021; habitat: Atlantic Forest; **Record Level:** type: PhysicalObject; institutionCode: CEIOC; basisOfRecord: PreservedSpecimen
- **Type status:** Other material. **Occurrence:** recordNumber: WCC118; recordedBy: M. M. Silva, A. J. Mayhé Nunes, J. C. Santos, I. R. S. Cordeiro; individualCount: 1; sex: female; lifeStage: adult; occurrenceID: 673414FC-4FB4-52F6-9C43-AFC00F2FF57A; **Taxon:** genus: Typhlomyrmex; specificEpithet: lavra; taxonRank: species; scientificNameAuthorship: (Lattke, 2002); **Location:** continent: South America; country: Brazil; countryCode: BR; stateProvince: Rio de Janeiro; municipality: Rio de Janeiro; locality: Floresta da Tijuca, Parque Nacional da Tijuca, Trilha de Moutain bike (Açude da solidão).; verbatimCoordinates: 22 57 40.0S 43 17 28.5W; verbatimLatitude: 22 57 40.0S; verbatimLongitude: 43 17 28.5W; verbatimCoordinateSystem: degrees minutes seconds; verbatimSRS: WGS84; **Identification:** identificationID: Typhlomyrmexlavra; identifiedBy: M. M. Silva; **Event:** samplingProtocol: Winckler; year: 2022; month: 1; day: 21-23; verbatimEventDate: Summer 2022; habitat: Atlantic Forest; **Record Level:** type: PhysicalObject; institutionCode: CEIOC; basisOfRecord: PreservedSpecimen

##### Distribution

Endemic from Brazil.

##### Notes

This is the first record for the State of Rio de Janeiro.

#### Formicinae

Latreille, 1809

41A036C7-3738-5309-92F8-604604A3C2EB

#### Acropyga decedens

(Mayr, 1887)

0A7B0A59-AB99-5F9C-A97C-60729202F74D

##### Materials

- **Type status:** Other material. **Occurrence:** recordNumber: WAS118; recordedBy: M. M. Silva, A. J. Mayhé Nunes, J. C. Santos, I. R. S. Cordeiro; individualCount: 1; sex: female; lifeStage: adult; occurrenceID: 577459FA-EC42-50E8-BC81-C0B99F36B2B0; **Taxon:** genus: Acropyga; specificEpithet: decedens; taxonRank: species; scientificNameAuthorship: (Mayr, 1887); **Location:** continent: South America; country: Brazil; countryCode: BR; stateProvince: Rio de Janeiro; municipality: Rio de Janeiro; locality: Floresta da Tijuca, Parque Nacional da Tijuca, Bom Retiro (trilha para o pico da Tijuca); verbatimCoordinates: 22 56 45.6S 43 17 32.5W; verbatimLatitude: 22 56 45.6S; verbatimLongitude: 43 17 32.5W; verbatimCoordinateSystem: degrees minutes seconds; verbatimSRS: WGS84; **Identification:** identificationID: Acropygadecedens; identifiedBy: M. M. Silva; **Event:** samplingProtocol: Winckler; year: 2021; month: 7; day: 19-21; verbatimEventDate: Winter 2021; habitat: Atlantic Forest; **Record Level:** type: PhysicalObject; institutionCode: CEIOC; basisOfRecord: PreservedSpecimen

##### Distribution

Brazil, Ecuador, French Guiana.

##### Notes

This is the first record for the PNT. Previously recorded from the State of Rio de Janeiro without details by Weber (1944).

#### Brachymyrmex admotus

Mayr, 1887

4599A040-1E97-5A5B-AD51-1718A596AB49

##### Materials

- **Type status:** Other material. **Occurrence:** recordNumber: WAS103; recordedBy: M. M. Silva, A. J. Mayhé Nunes, J. C. Santos, I. R. S. Cordeiro; individualCount: 7; sex: female; lifeStage: adult; occurrenceID: 9590DE81-383E-5788-BEFE-14D210C8C72F; **Taxon:** genus: Brachymyrmex; specificEpithet: admotus; taxonRank: species; scientificNameAuthorship: Mayr, 1887; **Location:** continent: South America; country: Brazil; countryCode: BR; stateProvince: Rio de Janeiro; municipality: Rio de Janeiro; locality: Floresta da Tijuca, Parque Nacional da Tijuca, Bom Retiro (trilha para o pico da Tijuca); verbatimCoordinates: 22 56 47.8S 43 17 30.4W; verbatimLatitude: 22 56 47.8S; verbatimLongitude: 43 17 30.4W; verbatimCoordinateSystem: degrees minutes seconds; verbatimSRS: WGS84; **Identification:** identificationID: Brachymyrmexadmotus; identifiedBy: M. M. Silva; **Event:** samplingProtocol: Winckler; year: 2021; month: 7; day: 19-21; verbatimEventDate: Winter 2021; habitat: Atlantic Forest; **Record Level:** type: PhysicalObject; institutionCode: CEIOC; basisOfRecord: PreservedSpecimen
- **Type status:** Other material. **Occurrence:** recordNumber: PBS104; recordedBy: M. M. Silva, A. J. Mayhé Nunes, J. C. Santos, I. R. S. Cordeiro; individualCount: 5; sex: female; lifeStage: adult; occurrenceID: 2755422E-B6DF-5F79-A3D9-342DDEBB7DB3; **Taxon:** genus: Brachymyrmex; specificEpithet: admotus; taxonRank: species; scientificNameAuthorship: Mayr, 1887; **Location:** continent: South America; country: Brazil; countryCode: BR; stateProvince: Rio de Janeiro; municipality: Rio de Janeiro; locality: Floresta da Tijuca, Parque Nacional da Tijuca, trilha (caminho dos picos/ camminho da solidão); verbatimCoordinates: 22 57 23.1S 43 16 57.9W; verbatimLatitude: 22 57 23.1S; verbatimLongitude: 43 16 57.9W; verbatimCoordinateSystem: degrees minutes seconds; verbatimSRS: WGS84; **Identification:** identificationID: Brachymyrmexadmotus; identifiedBy: M. M. Silva; **Event:** samplingProtocol: Winckler; year: 2021; month: 7; day: 21-23; verbatimEventDate: Winter 2021; habitat: Atlantic Forest; **Record Level:** type: PhysicalObject; institutionCode: CEIOC; basisOfRecord: PreservedSpecimen
- **Type status:** Other material. **Occurrence:** recordNumber: WCC119; recordedBy: M. M. Silva, A. J. Mayhé Nunes, J. C. Santos, I. R. S. Cordeiro; individualCount: 1; sex: female; lifeStage: adult; occurrenceID: 9BF5B189-1E38-5E4F-AF8A-4D209260EF54; **Taxon:** genus: Brachymyrmex; specificEpithet: admotus; taxonRank: species; scientificNameAuthorship: Mayr, 1887; **Location:** continent: South America; country: Brazil; countryCode: BR; stateProvince: Rio de Janeiro; municipality: Rio de Janeiro; locality: Floresta da Tijuca, Parque Nacional da Tijuca, Trilha de Moutain bike (Açude da solidão).; verbatimCoordinates: 22 57 40.0S 43 17 29.2W; verbatimLatitude: 22 57 40.0S; verbatimLongitude: 43 17 29.2W; verbatimCoordinateSystem: degrees minutes seconds; verbatimSRS: WGS84; **Identification:** identificationID: Brachymyrmexadmotus; identifiedBy: M. M. Silva; **Event:** samplingProtocol: Winckler; year: 2022; month: 1; day: 21-23; verbatimEventDate: Summer 2022; habitat: Atlantic Forest; **Record Level:** type: PhysicalObject; institutionCode: CEIOC; basisOfRecord: PreservedSpecimen

##### Distribution

Argentina, Brazil, French Guiana, Mexico, Panama, Paraguay, Uruguay, Venezuela.

##### Notes

This is the first record for the Floresta da Tijuca sector. Recorded from the Serra da Carioca sector by Santos et al. (2019).

#### Brachymyrmex australis

Forel, 1901

C889EDF5-7507-582F-9D2E-6A39B1B55B2D

##### Materials

- **Type status:** Other material. **Occurrence:** recordNumber: WAC103; recordedBy: M. M. Silva, A. J. Mayhé Nunes, J. C. Santos, I. R. S. Cordeiro; individualCount: 5; sex: female; lifeStage: adult; occurrenceID: C59CC7D8-25BC-5212-81A7-97DF6B7EF23F; **Taxon:** genus: Brachymyrmex; specificEpithet: australis; taxonRank: species; scientificNameAuthorship: Forel, 1901; **Location:** continent: South America; country: Brazil; countryCode: BR; stateProvince: Rio de Janeiro; municipality: Rio de Janeiro; locality: Floresta da Tijuca, Parque Nacional da Tijuca, Bom Retiro (trilha para o pico da Tijuca); verbatimCoordinates: 22 56 48.3S 43 17 30.2W; verbatimLatitude: 22 56 48.3S; verbatimLongitude: 43 17 30.2W; verbatimCoordinateSystem: degrees minutes seconds; verbatimSRS: WGS84; **Identification:** identificationID: Brachymyrmexaustralis; identifiedBy: M. M. Silva; **Event:** samplingProtocol: Winckler; year: 2022; month: 1; day: 17-19; verbatimEventDate: Summer 2022; habitat: Atlantic Forest; **Record Level:** type: PhysicalObject; institutionCode: CEIOC; basisOfRecord: PreservedSpecimen
- **Type status:** Other material. **Occurrence:** recordNumber: WBS113; recordedBy: M. M. Silva, A. J. Mayhé Nunes, J. C. Santos, I. R. S. Cordeiro; individualCount: 4; sex: female; lifeStage: adult; occurrenceID: 7FF2A111-704D-52B6-AC1D-C697F0EE5823; **Taxon:** genus: Brachymyrmex; specificEpithet: australis; taxonRank: species; scientificNameAuthorship: Forel, 1901; **Location:** continent: South America; country: Brazil; countryCode: BR; stateProvince: Rio de Janeiro; municipality: Rio de Janeiro; locality: Floresta da Tijuca, Parque Nacional da Tijuca, trilha (caminho dos picos/ camminho da solidão); verbatimCoordinates: 22 57 27.8S 43 16 57.3W; verbatimLatitude: 22 57 27.8S; verbatimLongitude: 43 16 57.3W; verbatimCoordinateSystem: degrees minutes seconds; verbatimSRS: WGS84; **Identification:** identificationID: Brachymyrmexaustralis; identifiedBy: M. M. Silva; **Event:** samplingProtocol: Winckler; year: 2021; month: 7; day: 21-23; verbatimEventDate: Winter 2021; habitat: Atlantic Forest; **Record Level:** type: PhysicalObject; institutionCode: CEIOC; basisOfRecord: PreservedSpecimen
- **Type status:** Other material. **Occurrence:** recordNumber: WCS119; recordedBy: M. M. Silva, A. J. Mayhé Nunes, J. C. Santos, I. R. S. Cordeiro; individualCount: 1; sex: female; lifeStage: adult; occurrenceID: BA46C87A-96B4-55BB-B2E2-B5FFA77FAE41; **Taxon:** genus: Brachymyrmex; specificEpithet: australis; taxonRank: species; scientificNameAuthorship: Forel, 1901; **Location:** continent: South America; country: Brazil; countryCode: BR; stateProvince: Rio de Janeiro; municipality: Rio de Janeiro; locality: Floresta da Tijuca, Parque Nacional da Tijuca, Trilha de Moutain bike (Açude da solidão).; verbatimCoordinates: 22 57 39.8S 43 17 30.9W; verbatimLatitude: 22 57 39.8S; verbatimLongitude: 43 17 30.9W; verbatimCoordinateSystem: degrees minutes seconds; verbatimSRS: WGS84; **Identification:** identificationID: Brachymyrmexaustralis; identifiedBy: M. M. Silva; **Event:** samplingProtocol: Winckler; year: 2021; month: 7; day: 23-25; verbatimEventDate: Winter 2021; habitat: Atlantic Forest; **Record Level:** type: PhysicalObject; institutionCode: CEIOC; basisOfRecord: PreservedSpecimen

##### Distribution

Argentina, Bahamas, Brazil, Colombia, Costa Rica, Cuba, Ecuador, French Guiana, Guatemala, Guyana, Hispaniola, Mexico, Panama, Paraguay, Peru, Uruguay. Exotic in the Mascarene Islands. Introduced to Germany.

##### Notes

This is the first record for the PNT. Ortiz-Sepulveda et al. (2019) recorded this species from another protected area in the State of Rio de Janeiro state.

#### Brachymyrmex bruchi

Forel, 1912

A7626279-6FAE-518C-AD4E-A7F770C43622

##### Materials

- **Type status:** Other material. **Occurrence:** recordNumber: WAS103; recordedBy: M. M. Silva, A. J. Mayhé Nunes, J. C. Santos, I. R. S. Cordeiro; individualCount: 1; sex: female; lifeStage: adult; occurrenceID: 9E5DA292-D124-5267-9031-50FB3EB98270; **Taxon:** genus: Brachymyrmex; specificEpithet: bruchi; taxonRank: species; scientificNameAuthorship: Forel, 1912; **Location:** continent: South America; country: Brazil; countryCode: BR; stateProvince: Rio de Janeiro; municipality: Rio de Janeiro; locality: Floresta da Tijuca, Parque Nacional da Tijuca, Bom Retiro (trilha para o pico da Tijuca); verbatimCoordinates: 22 56 47.8S 43 17 30.4W; verbatimLatitude: 22 56 47.8S; verbatimLongitude: 43 17 30.4W; verbatimCoordinateSystem: degrees minutes seconds; verbatimSRS: WGS84; **Identification:** identificationID: Brachymyrmexbruchi; identifiedBy: M. M. Silva; **Event:** samplingProtocol: Winckler; year: 2021; month: 7; day: 19-21; verbatimEventDate: Winter 2021; habitat: Atlantic Forest; **Record Level:** type: PhysicalObject; institutionCode: CEIOC; basisOfRecord: PreservedSpecimen

##### Distribution

Argentina, Bolivia, Brazil, Chile, Colombia, Ecuador, Guatemala, Hispaniola, Paraguay, United States, Uruguay.

##### Notes

This is the first record for State of Rio de Janeiro state, which extends the known range eastwards.

#### Brachymyrmex degener

Emery, 1906

ED687629-CC6D-569B-8432-CFD599F42203

##### Materials

- **Type status:** Other material. **Occurrence:** recordNumber: WAS102; recordedBy: M. M. Silva, A. J. Mayhé Nunes, J. C. Santos, I. R. S. Cordeiro; individualCount: 1; sex: female; lifeStage: adult; occurrenceID: 2B620AF0-0C3F-508A-A08D-5FF59E5D8A6A; **Taxon:** genus: Brachymyrmex; specificEpithet: degener; taxonRank: species; scientificNameAuthorship: Emery, 1906; **Location:** continent: South America; country: Brazil; countryCode: BR; stateProvince: Rio de Janeiro; municipality: Rio de Janeiro; locality: Floresta da Tijuca, Parque Nacional da Tijuca, Bom Retiro (trilha para o pico da Tijuca); verbatimCoordinates: 22 56 47.5S 43 17 30.6W; verbatimLatitude: 22 56 47.5S; verbatimLongitude: 43 17 30.6W; verbatimCoordinateSystem: degrees minutes seconds; verbatimSRS: WGS84; **Identification:** identificationID: Brachymyrmexdegener; identifiedBy: M. M. Silva; **Event:** samplingProtocol: Winckler; year: 2021; month: 7; day: 19-21; verbatimEventDate: Winter 2021; habitat: Atlantic Forest; **Record Level:** type: PhysicalObject; institutionCode: CEIOC; basisOfRecord: PreservedSpecimen

##### Distribution

Argentina, Brazil, Colombia, French Guiana, Guatemala, Guyana, Mexico, Panama, Paraguay, Trinidad and Tobago.

##### Notes

This is the first record for the State of Rio de Janeiro, which contributes to fill a gap between the States of Bahia to the north and São Paulo to the south.

#### Camponotus atriceps

(Smith, F., 1858)

3AC69C0F-E8EE-5D2F-BAE4-6872B7BE67E9

##### Materials

- **Type status:** Other material. **Occurrence:** recordNumber: PAC115; recordedBy: M. M. Silva, A. J. Mayhé Nunes, J. C. Santos, I. R. S. Cordeiro; individualCount: 1; sex: female; lifeStage: adult; occurrenceID: 4EEE1996-CF14-5860-BCBB-E9209390FDC4; **Taxon:** genus: Camponotus; specificEpithet: atriceps; taxonRank: species; scientificNameAuthorship: (Smith, F., 1858); **Location:** continent: South America; country: Brazil; countryCode: BR; stateProvince: Rio de Janeiro; municipality: Rio de Janeiro; locality: Floresta da Tijuca, Parque Nacional da Tijuca, Bom Retiro (trilha para o pico da Tijuca); verbatimCoordinates: 22 56 49.5S 43 17 29.8W; verbatimLatitude: 22 56 49.5S; verbatimLongitude: 43 17 29.8W; verbatimCoordinateSystem: degrees minutes seconds; verbatimSRS: WGS84; **Identification:** identificationID: Camponotusatriceps; identifiedBy: M. M. Silva; **Event:** samplingProtocol: Pitfall; year: 2022; month: 1; day: 17-19; verbatimEventDate: Summer 2022; habitat: Atlantic Forest; **Record Level:** type: PhysicalObject; institutionCode: CEIOC; basisOfRecord: PreservedSpecimen
- **Type status:** Other material. **Occurrence:** recordNumber: PBC116; recordedBy: M. M. Silva, A. J. Mayhé Nunes, J. C. Santos, I. R. S. Cordeiro; individualCount: 1; sex: female; lifeStage: adult; occurrenceID: E0B31290-09D7-5CEB-861B-4C32578B1B80; **Taxon:** genus: Camponotus; specificEpithet: atriceps; taxonRank: species; scientificNameAuthorship: (Smith, F., 1858); **Location:** continent: South America; country: Brazil; countryCode: BR; stateProvince: Rio de Janeiro; municipality: Rio de Janeiro; locality: Floresta da Tijuca, Parque Nacional da Tijuca, trilha (caminho dos picos/ camminho da solidão); verbatimCoordinates: 22 57 24.6S 43 17 02.0W; verbatimLatitude: 22 57 24.6S; verbatimLongitude: 43 17 02.0W; verbatimCoordinateSystem: degrees minutes seconds; verbatimSRS: WGS84; **Identification:** identificationID: Camponotusatriceps; identifiedBy: M. M. Silva; **Event:** samplingProtocol: Pitfall; year: 2021; month: 1; day: 17-21; verbatimEventDate: Summer 2021; habitat: Atlantic Forest; **Record Level:** type: PhysicalObject; institutionCode: CEIOC; basisOfRecord: PreservedSpecimen
- **Type status:** Other material. **Occurrence:** recordNumber: PCS102; recordedBy: M. M. Silva, A. J. Mayhé Nunes, J. C. Santos, I. R. S. Cordeiro; individualCount: 1; sex: female; lifeStage: adult; occurrenceID: 2A87E737-0C87-5F75-AF9A-301C4CEB1927; **Taxon:** genus: Camponotus; specificEpithet: atriceps; taxonRank: species; scientificNameAuthorship: (Smith, F., 1858); **Location:** continent: South America; country: Brazil; countryCode: BR; stateProvince: Rio de Janeiro; municipality: Rio de Janeiro; locality: Floresta da Tijuca, Parque Nacional da Tijuca, Trilha de Moutain bike (Açude da solidão).; verbatimCoordinates: 22 57 42.5S 43 17 23.8W; verbatimLatitude: 22 57 42.5S; verbatimLongitude: 43 17 23.8W; verbatimCoordinateSystem: degrees minutes seconds; verbatimSRS: WGS84; **Identification:** identificationID: Camponotusatriceps; identifiedBy: M. M. Silva; **Event:** samplingProtocol: Pitfall; year: 2021; month: 7; day: 23-25; verbatimEventDate: Winter 2021; habitat: Atlantic Forest; **Record Level:** type: PhysicalObject; institutionCode: CEIOC; basisOfRecord: PreservedSpecimen

##### Distribution

Argentina, Belize, Bolivia, Brazil, Colombia, Costa Rica, Ecuador, El Salvador, French Guiana, Guatemala, Guyana, Honduras, Lesser Antilles, Mexico, Nicaragua, Panama, Paraguay, Peru, Suriname, Trinidad and Tobago, United States, Uruguay, Venezuela. Introduced to Hawaii, the Netherlands, United Kingdom and Italy. The record from Cuba needs verification, whereas that from Hispaniola is dubious.

##### Notes

This is the first record for the Floresta da Tijuca sector. This species was recorded by Santos et al. (2019) from the Rio de Janeiro Botanical Garden and the Parque Lage (a urban park), both in the Serra da Carioca sector.

#### Myrmelachista bettinae

Forel, 1903

3F0682BA-D199-5E3E-AFA4-B9E5DC6757B6

##### Materials

- **Type status:** Other material. **Occurrence:** recordNumber: PBC106; recordedBy: M. M. Silva, A. J. Mayhé Nunes, J. C. Santos, I. R. S. Cordeiro; individualCount: 1; sex: female; lifeStage: adult; occurrenceID: 3BC1F4A8-5B56-5078-B8EB-802F6004780A; **Taxon:** genus: Myrmelachista; specificEpithet: bettinae; taxonRank: species; scientificNameAuthorship: Forel, 1903; **Location:** continent: South America; country: Brazil; countryCode: BR; stateProvince: Rio de Janeiro; municipality: Rio de Janeiro; locality: Floresta da Tijuca, Parque Nacional da Tijuca, trilha (caminho dos picos/ camminho da solidão); verbatimCoordinates: 22 57 23.3S 43 16 59.4W; verbatimLatitude: 22 57 23.3S; verbatimLongitude: 43 16 59.4W; verbatimCoordinateSystem: degrees minutes seconds; verbatimSRS: WGS84; **Identification:** identificationID: Myrmelachistabettinae; identifiedBy: M. M. Silva; **Event:** samplingProtocol: Pitfall; year: 2022; month: 1; day: 17-21; verbatimEventDate: Summer 2022; habitat: Atlantic Forest; **Record Level:** type: PhysicalObject; institutionCode: CEIOC; basisOfRecord: PreservedSpecimen

##### Distribution

Endemic from the State of Rio de Janeiro.

##### Notes

This is the first record for the Floresta da Tijuca sector. Kempf (1972) recorded it from the Serra da Carioca sector.

#### Nylanderia docilis

(Forel, 1908)

84F8A2F9-03E8-599D-8133-927AC0D8098D

##### Materials

- **Type status:** Other material. **Occurrence:** recordNumber: WBC103; recordedBy: M. M. Silva, A. J. Mayhé Nunes, J. C. Santos, I. R. S. Cordeiro; individualCount: 12; sex: female; lifeStage: adult; occurrenceID: C2421B45-534A-5390-8E4E-75EF9FC8F661; **Taxon:** genus: Nylanderia; specificEpithet: docilis; taxonRank: species; scientificNameAuthorship: (Forel, 1908); **Location:** continent: South America; country: Brazil; countryCode: BR; stateProvince: Rio de Janeiro; municipality: Rio de Janeiro; locality: Floresta da Tijuca, Parque Nacional da Tijuca, trilha (caminho dos picos/ camminho da solidão); verbatimCoordinates: 22 57 23.1S 43 16 59.0W; verbatimLatitude: 22 57 23.1S; verbatimLongitude: 43 16 59.0W; verbatimCoordinateSystem: degrees minutes seconds; verbatimSRS: WGS84; **Identification:** identificationID: Nylanderiadocilis; identifiedBy: M. M. Silva; **Event:** samplingProtocol: Winckler; year: 2022; month: 1; day: 17-21; verbatimEventDate: Summer 2022; habitat: Atlantic Forest; **Record Level:** type: PhysicalObject; institutionCode: CEIOC; basisOfRecord: PreservedSpecimen
- **Type status:** Other material. **Occurrence:** recordNumber: WCC106; recordedBy: M. M. Silva, A. J. Mayhé Nunes, J. C. Santos, I. R. S. Cordeiro; individualCount: 6; sex: female; lifeStage: adult; occurrenceID: 3B3DB01F-2C70-562F-9C43-5B309426CB13; **Taxon:** genus: Nylanderia; specificEpithet: docilis; taxonRank: species; scientificNameAuthorship: (Forel, 1908); **Location:** continent: South America; country: Brazil; countryCode: BR; stateProvince: Rio de Janeiro; municipality: Rio de Janeiro; locality: Floresta da Tijuca, Parque Nacional da Tijuca, Trilha de Moutain bike (Açude da solidão).; verbatimCoordinates: 22 57 42.2S 43 17 23.9W; verbatimLatitude: 22 57 42.2S; verbatimLongitude: 43 17 23.9W; verbatimCoordinateSystem: degrees minutes seconds; verbatimSRS: WGS84; **Identification:** identificationID: Nylanderiadocilis; identifiedBy: M. M. Silva; **Event:** samplingProtocol: Winckler; year: 2022; month: 1; day: 22-23; verbatimEventDate: Summer 2022; habitat: Atlantic Forest; **Record Level:** type: PhysicalObject; institutionCode: CEIOC; basisOfRecord: PreservedSpecimen

##### Distribution

Argentina, Brazil, Paraguay.

##### Notes

This is the first record for the PNT, which extends the known distribution eastwards.

#### Nylanderia fulva

(Mayr, 1862)

A24B92DE-4280-5AB2-B1B2-26E25FBA8FCA

##### Materials

- **Type status:** Other material. **Occurrence:** recordNumber: WBS101; recordedBy: M. M. Silva, A. J. Mayhé Nunes, J. C. Santos, I. R. S. Cordeiro; individualCount: 1; sex: female; lifeStage: adult; occurrenceID: 2A94E967-60C7-54CD-8508-3BC7E7654C0A; **Taxon:** genus: Nylanderia; specificEpithet: fulva; taxonRank: species; scientificNameAuthorship: (Mayr, 1862); **Location:** continent: South America; country: Brazil; countryCode: BR; stateProvince: Rio de Janeiro; municipality: Rio de Janeiro; locality: Floresta da Tijuca, Parque Nacional da Tijuca, Bom Retiro (trilha para o pico da Tijuca); verbatimCoordinates: 22 56 43.1S 43 17 29.5W; verbatimLatitude: 22 56 43.1S; verbatimLongitude: 43 17 29.5W; verbatimCoordinateSystem: degrees minutes seconds; verbatimSRS: WGS84; **Identification:** identificationID: Nylanderiafulva; identifiedBy: M. M. Silva; **Event:** samplingProtocol: Winckler; year: 2022; month: 1; day: 17-19; verbatimEventDate: Summer 2022; habitat: Atlantic Forest; **Record Level:** type: PhysicalObject; institutionCode: CEIOC; basisOfRecord: PreservedSpecimen
- **Type status:** Other material. **Occurrence:** recordNumber: WAC119; recordedBy: M. M. Silva, A. J. Mayhé Nunes, J. C. Santos, I. R. S. Cordeiro; individualCount: 1; sex: female; lifeStage: adult; occurrenceID: 51021DAA-0C74-55BB-9298-91D2FA81D215; **Taxon:** genus: Nylanderia; specificEpithet: fulva; taxonRank: species; scientificNameAuthorship: (Mayr, 1862); **Location:** continent: South America; country: Brazil; countryCode: BR; stateProvince: Rio de Janeiro; municipality: Rio de Janeiro; locality: Floresta da Tijuca, Parque Nacional da Tijuca, trilha (caminho dos picos/ camminho da solidão); verbatimCoordinates: 22 57 23.1S 43 16 59.0W; verbatimLatitude: 22 57 23.1S; verbatimLongitude: 43 16 59.0W; verbatimCoordinateSystem: degrees minutes seconds; verbatimSRS: WGS84; **Identification:** identificationID: Nylanderiafulva; identifiedBy: M. M. Silva; **Event:** samplingProtocol: Winckler; year: 2021; month: 7; day: 21-23; verbatimEventDate: Winter 2021; habitat: Atlantic Forest; **Record Level:** type: PhysicalObject; institutionCode: CEIOC; basisOfRecord: PreservedSpecimen

##### Distribution

Argentina, Brazil, Bolivia, Paraguay, Uruguay. Exotic in northern Brazil, the Canary Islands, Colombia, Costa Rica, Cuba, Ecuador, French Guiana, Galápagos Islands, Guyana, Hispaniola, Jamaica, Lesser Antilles, Mexico, Puerto Rico, Suriname, Trinidad and Tobago, United States and Venezuela. Introduced in Canada and France.

##### Notes

This is the first record for the PNT. Kempf (1972) and Brandão (1991) recorded this species from the State of Rio de Janeiro without details.

#### Nylanderia goeldii

(Forel, 1912)

0DBC1D24-0DEC-58A4-B487-6FF5BEC6A138

##### Materials

- **Type status:** Other material. **Occurrence:** recordNumber: WAS104; recordedBy: M. M. Silva, A. J. Mayhé Nunes, J. C. Santos, I. R. S. Cordeiro; individualCount: 1; sex: female; lifeStage: adult; occurrenceID: 4DCCFCBE-1CB0-50FE-89DC-EEEA717ADEED; **Taxon:** genus: Nylanderia; specificEpithet: goeldii; taxonRank: species; scientificNameAuthorship: (Forel, 1912); **Location:** continent: South America; country: Brazil; countryCode: BR; stateProvince: Rio de Janeiro; municipality: Rio de Janeiro; locality: Floresta da Tijuca, Parque Nacional da Tijuca, Bom Retiro (trilha para o pico da Tijuca); verbatimCoordinates: 22 56 48.2S 43 17 29.8W; verbatimLatitude: 22 56 48.2S; verbatimLongitude: 43 17 29.8W; verbatimCoordinateSystem: degrees minutes seconds; verbatimSRS: WGS84; **Identification:** identificationID: Nylanderiagoeldii; identifiedBy: M. M. Silva; **Event:** samplingProtocol: Winckler; year: 2021; month: 7; day: 19-21; verbatimEventDate: Winter 2021; habitat: Atlantic Forest; **Record Level:** type: PhysicalObject; institutionCode: CEIOC; basisOfRecord: PreservedSpecimen
- **Type status:** Other material. **Occurrence:** recordNumber: WBS105; recordedBy: M. M. Silva, A. J. Mayhé Nunes, J. C. Santos, I. R. S. Cordeiro; individualCount: 2; sex: female; lifeStage: adult; occurrenceID: 70AECECB-40A6-57A3-BA9A-198E10FF1B0F; **Taxon:** genus: Nylanderia; specificEpithet: goeldii; taxonRank: species; scientificNameAuthorship: (Forel, 1912); **Location:** continent: South America; country: Brazil; countryCode: BR; stateProvince: Rio de Janeiro; municipality: Rio de Janeiro; locality: Floresta da Tijuca, Parque Nacional da Tijuca, trilha (caminho dos picos/ camminho da solidão); verbatimCoordinates: 22 57 23.8S 43 16 57.7W; verbatimLatitude: 22 57 23.8S; verbatimLongitude: 43 16 57.7W; verbatimCoordinateSystem: degrees minutes seconds; verbatimSRS: WGS84; **Identification:** identificationID: Nylanderiagoeldii; identifiedBy: M. M. Silva; **Event:** samplingProtocol: Winckler; year: 2021; month: 7; day: 21-23; verbatimEventDate: Winter 2021; habitat: Atlantic Forest; **Record Level:** type: PhysicalObject; institutionCode: CEIOC; basisOfRecord: PreservedSpecimen

##### Distribution

Endemic from south-eastern Brazil.

##### Notes

This is the first record for the PNT. Previously recorded by Kempf (1972) from the State of Rio de Janeiro without details.

#### Nylanderia guatemalensis

(Forel, 1885)

686E513D-4A85-5987-B00E-9CA3028BB550

##### Materials

- **Type status:** Other material. **Occurrence:** recordNumber: WAC105; recordedBy: M. M. Silva, A. J. Mayhé Nunes, J. C. Santos, I. R. S. Cordeiro; individualCount: 2; sex: female; lifeStage: adult; occurrenceID: 97098107-A66D-5809-8BDB-7B87B957FC2B; **Taxon:** genus: Nylanderia; specificEpithet: guatemalensis; taxonRank: species; scientificNameAuthorship: (Forel, 1885); **Location:** continent: South America; country: Brazil; countryCode: BR; stateProvince: Rio de Janeiro; municipality: Rio de Janeiro; locality: Floresta da Tijuca, Parque Nacional da Tijuca, Bom Retiro (trilha para o pico da Tijuca); verbatimCoordinates: 22 56 47.7S 43 17 33.1W; verbatimLatitude: 22 56 47.7S; verbatimLongitude: 43 17 33.1W; verbatimCoordinateSystem: degrees minutes seconds; verbatimSRS: WGS84; **Identification:** identificationID: Nylanderiaguatemalensis; identifiedBy: M. M. Silva; **Event:** samplingProtocol: Winckler; year: 2022; month: 1; day: 17-21; verbatimEventDate: Summer 2022; habitat: Atlantic Forest; **Record Level:** type: PhysicalObject; institutionCode: CEIOC; basisOfRecord: PreservedSpecimen
- **Type status:** Other material. **Occurrence:** recordNumber: PBC106; recordedBy: M. M. Silva, A. J. Mayhé Nunes, J. C. Santos, I. R. S. Cordeiro; individualCount: 4; sex: female; lifeStage: adult; occurrenceID: 747E7ACE-8B08-5E98-9623-D68E2DB83B11; **Taxon:** genus: Nylanderia; specificEpithet: guatemalensis; taxonRank: species; scientificNameAuthorship: (Forel, 1885); **Location:** continent: South America; country: Brazil; countryCode: BR; stateProvince: Rio de Janeiro; municipality: Rio de Janeiro; locality: Floresta da Tijuca, Parque Nacional da Tijuca, trilha (caminho dos picos/ camminho da solidão); verbatimCoordinates: 22 57 23.7S 43 16 59.7W; verbatimLatitude: 22 57 23.7S; verbatimLongitude: 43 16 59.7W; verbatimCoordinateSystem: degrees minutes seconds; verbatimSRS: WGS84; **Identification:** identificationID: Nylanderiaguatemalensis; identifiedBy: M. M. Silva; **Event:** samplingProtocol: Pitfall; year: 2022; month: 1; day: 17-21; verbatimEventDate: Summer 2022; habitat: Atlantic Forest; **Record Level:** type: PhysicalObject; institutionCode: CEIOC; basisOfRecord: PreservedSpecimen

##### Distribution

Belize, Brazil, Colombia, Costa Rica, Ecuador, French Guiana, Guatemala, Honduras, Mexico, Nicaragua, Panama, Venezuela. Exotic in northern Mexico, south-eastern United States, Bahamas, Cocos Island, Cuba, Galápagos Islands, Hispaniola, Jamaica, Lesser Antilles and Trinidad and Tobago. Introduced to mid-western United States, Germany, Netherlands and the United Kingdom. Records from south-western United States and the State of Sonora (Mexico) need verification.

##### Notes

This is the first record for the Floresta da Tijuca sector. This species was recorded by Santos et al. (2019) from the Reserva Florestal da Vista Chinesa, in the Serra da Carioca sector.

#### Nylanderia steinheili

(Forel, 1893)

C02CD371-0430-53BE-9831-E57F452C1377

##### Materials

- **Type status:** Other material. **Occurrence:** recordNumber: WAC120; recordedBy: M. M. Silva, A. J. Mayhé Nunes, J. C. Santos, I. R. S. Cordeiro; individualCount: 1; sex: female; lifeStage: adult; occurrenceID: 775FE0BD-78E6-52AC-A43E-69AC2A6A2CF7; **Taxon:** genus: Nylanderia; specificEpithet: steinheili; taxonRank: species; scientificNameAuthorship: (Forel, 1893); **Location:** continent: South America; country: Brazil; countryCode: BR; stateProvince: Rio de Janeiro; municipality: Rio de Janeiro; locality: Floresta da Tijuca, Parque Nacional da Tijuca, Bom Retiro (trilha para o pico da Tijuca); verbatimCoordinates: 22 56 43.3S 43 17 28.9W; verbatimLatitude: 22 56 43.3S; verbatimLongitude: 43 17 28.9W; verbatimCoordinateSystem: degrees minutes seconds; verbatimSRS: WGS84; **Identification:** identificationID: Nylanderiasteinheili; identifiedBy: M. M. Silva; **Event:** samplingProtocol: Winckler; year: 2022; month: 1; day: 17-19; verbatimEventDate: Summer 2022; habitat: Atlantic Forest; **Record Level:** type: PhysicalObject; institutionCode: CEIOC; basisOfRecord: PreservedSpecimen
- **Type status:** Other material. **Occurrence:** recordNumber: WBS107; recordedBy: M. M. Silva, A. J. Mayhé Nunes, J. C. Santos, I. R. S. Cordeiro; individualCount: 5; sex: female; lifeStage: adult; occurrenceID: F3A73076-3206-5B39-9611-D1FE155A3D81; **Taxon:** genus: Nylanderia; specificEpithet: steinheili; taxonRank: species; scientificNameAuthorship: (Forel, 1893); **Location:** continent: South America; country: Brazil; countryCode: BR; stateProvince: Rio de Janeiro; municipality: Rio de Janeiro; locality: Floresta da Tijuca, Parque Nacional da Tijuca, trilha (caminho dos picos/ camminho da solidão); verbatimCoordinates: 22 57 24.4S 43 16 56.7W; verbatimLatitude: 22 57 24.4S; verbatimLongitude: 43 16 56.7W; verbatimCoordinateSystem: degrees minutes seconds; verbatimSRS: WGS84; **Identification:** identificationID: Nylanderiasteinheili; identifiedBy: M. M. Silva; **Event:** samplingProtocol: Winckler; year: 2021; month: 7; day: 21-23; verbatimEventDate: Winter 2021; habitat: Atlantic Forest; **Record Level:** type: PhysicalObject; institutionCode: CEIOC; basisOfRecord: PreservedSpecimen
- **Type status:** Other material. **Occurrence:** recordNumber: WCC106; recordedBy: M. M. Silva, A. J. Mayhé Nunes, J. C. Santos, I. R. S. Cordeiro; individualCount: 1; sex: female; lifeStage: adult; occurrenceID: 941A9A40-276A-52DD-8B79-B8F3AAF3DFC9; **Taxon:** genus: Nylanderia; specificEpithet: steinheili; taxonRank: species; scientificNameAuthorship: (Forel, 1893); **Location:** continent: South America; country: Brazil; countryCode: BR; stateProvince: Rio de Janeiro; municipality: Rio de Janeiro; locality: Floresta da Tijuca, Parque Nacional da Tijuca, Trilha de Moutain bike (Açude da solidão).; verbatimCoordinates: 22 57 42.2S 43 17 23.9W; verbatimLatitude: 22 57 42.2S; verbatimLongitude: 43 17 23.9W; verbatimCoordinateSystem: degrees minutes seconds; verbatimSRS: WGS84; **Identification:** identificationID: Nylanderiasteinheili; identifiedBy: M. M. Silva; **Event:** samplingProtocol: Winckler; year: 2022; month: 1; day: 21-23; verbatimEventDate: Summer 2022; habitat: Atlantic Forest; **Record Level:** type: PhysicalObject; institutionCode: CEIOC; basisOfRecord: PreservedSpecimen

##### Distribution

Brazil, Colombia, Costa Rica, French Guiana, Guatemala, Honduras, Mexico, Nicaragua, Panama, Venezuela. Exotic in Argentina, Bahamas, Cuba, Galápagos Islands, Hispianiola, Jamaica, Lesser Antilles, Seychelles and the United States. Introduced to Norway, United Kingdom and the Netherlands.

##### Notes

This is the first record for the PNT. Previously recorded by Kempf (1972) and Brandão (1991) from the State of Rio de Janeiro without details.

#### Myrmicinae

Lepeletier de Saint-Fargeau, 1835

1658A8E3-D69A-509A-B4D3-FD6A941738EC

#### Acanthognathus brevicornis

Smith, 1944

132FDB8E-5BCA-5D1E-B0F1-A0D3B4F3D96A

##### Materials

- **Type status:** Other material. **Occurrence:** recordNumber: WAC120; recordedBy: M. M. Silva, A. J. Mayhé Nunes, J. C. Santos, I. R. S. Cordeiro; individualCount: 4; sex: female; lifeStage: adult; occurrenceID: 36B393FE-6EFB-50A4-9432-C574598F842B; **Taxon:** genus: Acanthognathus; specificEpithet: brevicornis; taxonRank: species; scientificNameAuthorship: Smith, 1944; **Location:** continent: South America; country: Brazil; countryCode: BR; stateProvince: Rio de Janeiro; municipality: Rio de Janeiro; locality: Floresta da Tijuca, Parque Nacional da Tijuca, Bom Retiro (trilha para o pico da Tijuca); verbatimCoordinates: 22 56 43.3S 43 17 28.9W; verbatimLatitude: 22 56 43.3S; verbatimLongitude: 43 17 28.9W; verbatimCoordinateSystem: degrees minutes seconds; verbatimSRS: WGS84; **Identification:** identificationID: Acanthognathusbrevicornis; identifiedBy: M. M. Silva; **Event:** samplingProtocol: Winckler; year: 2022; month: 1; day: 17-19; verbatimEventDate: Summer 2022; habitat: Atlantic Forest; **Record Level:** type: PhysicalObject; institutionCode: CEIOC; basisOfRecord: PreservedSpecimen
- **Type status:** Other material. **Occurrence:** recordNumber: WCS102; recordedBy: M. M. Silva, A. J. Mayhé Nunes, J. C. Santos, I. R. S. Cordeiro; individualCount: 9; sex: female; lifeStage: adult; occurrenceID: 53C8A2B1-AC95-525E-B9D2-A1B49EBC4FDE; **Taxon:** genus: Acanthognathus; specificEpithet: brevicornis; taxonRank: species; scientificNameAuthorship: Smith, 1944; **Location:** continent: South America; country: Brazil; countryCode: BR; stateProvince: Rio de Janeiro; municipality: Rio de Janeiro; locality: Floresta da Tijuca, Parque Nacional da Tijuca, Trilha de Moutain bike (Açude da solidão).; verbatimCoordinates: 22 57 42.0S 43 17 23.9W; verbatimLatitude: 22 57 42.0S; verbatimLongitude: 43 17 23.9W; verbatimCoordinateSystem: degrees minutes seconds; verbatimSRS: WGS84; **Identification:** identificationID: Acanthognathusbrevicornis; identifiedBy: M. M. Silva; **Event:** samplingProtocol: Winckler; year: 2021; month: 7; day: 23-25; verbatimEventDate: Winter 2021; habitat: Atlantic Forest; **Record Level:** type: PhysicalObject; institutionCode: CEIOC; basisOfRecord: PreservedSpecimen

##### Distribution

Brazil, Colombia, Ecuador, French Guiana, Guyana, Panama, Peru.

##### Notes

This is the first record for the PNT. Silva and Brandão (2014) and Montine et al. (2014) recorded this species from other protected areas in the State of Rio de Janeiro.

#### Acromyrmex brunneus

(Forel, 1912)

494876B0-A0F5-588E-9B07-95DE8C0CD8A2

##### Materials

- **Type status:** Other material. **Occurrence:** recordNumber: WBS101; recordedBy: M. M. Silva, A. J. Mayhé Nunes, J. C. Santos, I. R. S. Cordeiro; individualCount: 6; sex: female; lifeStage: adult; occurrenceID: D44986DB-D220-57C8-83B3-4C687DE8D4E8; **Taxon:** genus: Acromyrmex; specificEpithet: brunneus; taxonRank: species; scientificNameAuthorship: (Forel, 1912); **Location:** continent: South America; country: Brazil; countryCode: BR; stateProvince: Rio de Janeiro; municipality: Rio de Janeiro; locality: Floresta da Tijuca, Parque Nacional da Tijuca, trilha (caminho dos picos/ camminho da solidão); verbatimCoordinates: 22 57 23.5S 43 17 00.1W; verbatimLatitude: 22 57 23.5S; verbatimLongitude: 43 17 00.1W; verbatimCoordinateSystem: degrees minutes seconds; verbatimSRS: WGS84; **Identification:** identificationID: Acromyrmexbrunneus; identifiedBy: M. M. Silva; **Event:** samplingProtocol: Winckler; year: 2021; month: 7; day: 21-23; verbatimEventDate: Winter 2021; habitat: Atlantic Forest; **Record Level:** type: PhysicalObject; institutionCode: CEIOC; basisOfRecord: PreservedSpecimen
- **Type status:** Other material. **Occurrence:** recordNumber: WCS104; recordedBy: M. M. Silva, A. J. Mayhé Nunes, J. C. Santos, I. R. S. Cordeiro; individualCount: 2; sex: female; lifeStage: adult; occurrenceID: 70B1A979-4199-59E1-89A7-CB778484EA9C; **Taxon:** genus: Acromyrmex; specificEpithet: brunneus; taxonRank: species; scientificNameAuthorship: (Forel, 1912); **Location:** continent: South America; country: Brazil; countryCode: BR; stateProvince: Rio de Janeiro; municipality: Rio de Janeiro; locality: Floresta da Tijuca, Parque Nacional da Tijuca, Trilha de Moutain bike (Açude da solidão).; verbatimCoordinates: 22 57 41.8S 43 17 24.8W; verbatimLatitude: 22 57 41.8S; verbatimLongitude: 43 17 24.8W; verbatimCoordinateSystem: degrees minutes seconds; verbatimSRS: WGS84; **Identification:** identificationID: Acromyrmexbrunneus; identifiedBy: M. M. Silva; **Event:** samplingProtocol: Winckler; year: 2021; month: 7; day: 23-25; verbatimEventDate: Winter 2021; habitat: Atlantic Forest; **Record Level:** type: PhysicalObject; institutionCode: CEIOC; basisOfRecord: PreservedSpecimen

##### Distribution

Brazil, Paraguay.

##### Notes

Previously recorded from "Floresta da Tijuca" by Gonçalves (1961) and from the Serra da Carioca sector by Santos et al. (2019).

#### Acromyrmex molestans

Santschi, 1925

2D66F55A-AFDF-5AC0-AF1B-5A2B42F4D628

##### Materials

- **Type status:** Other material. **Occurrence:** recordNumber: WAC118; recordedBy: M. M. Silva, A. J. Mayhé Nunes, J. C. Santos, I. R. S. Cordeiro; individualCount: 2; sex: female; lifeStage: adult; occurrenceID: CE69AE08-CF7C-5F01-A4F7-E04E85DC7DD7; **Taxon:** genus: Acromyrmex; specificEpithet: molestans; taxonRank: species; scientificNameAuthorship: Santschi, 1925; **Location:** continent: South America; country: Brazil; countryCode: BR; stateProvince: Rio de Janeiro; municipality: Rio de Janeiro; locality: Floresta da Tijuca, Parque Nacional da Tijuca, Bom Retiro (trilha para o pico da Tijuca); verbatimCoordinates: 22 56 43.6S 43 17 30.1W; verbatimLatitude: 22 56 43.6S; verbatimLongitude: 43 17 30.1W; verbatimCoordinateSystem: degrees minutes seconds; verbatimSRS: WGS84; **Identification:** identificationID: Acromyrmexmolestans; identifiedBy: M. M. Silva; **Event:** samplingProtocol: Winckler; year: 2022; month: 1; day: 17-19; verbatimEventDate: Summer 2022; habitat: Atlantic Forest; **Record Level:** type: PhysicalObject; institutionCode: CEIOC; basisOfRecord: PreservedSpecimen
- **Type status:** Other material. **Occurrence:** recordNumber: WBC108; recordedBy: M. M. Silva, A. J. Mayhé Nunes, J. C. Santos, I. R. S. Cordeiro; individualCount: 1; sex: female; lifeStage: adult; occurrenceID: 445672E5-A794-5595-A3A5-2B9AD755AEF1; **Taxon:** genus: Acromyrmex; specificEpithet: molestans; taxonRank: species; scientificNameAuthorship: Santschi, 1925; **Location:** continent: South America; country: Brazil; countryCode: BR; stateProvince: Rio de Janeiro; municipality: Rio de Janeiro; locality: Floresta da Tijuca, Parque Nacional da Tijuca, trilha (caminho dos picos/ camminho da solidão); verbatimCoordinates: 22 57 24.5S 43 16 56.7W; verbatimLatitude: 22 57 24.5S; verbatimLongitude: 43 16 56.7W; verbatimCoordinateSystem: degrees minutes seconds; verbatimSRS: WGS84; **Identification:** identificationID: Acromyrmexmolestans; identifiedBy: M. M. Silva; **Event:** samplingProtocol: Winckler; year: 2022; month: 1; day: 19-21; verbatimEventDate: Summer 2022; habitat: Atlantic Forest; **Record Level:** type: PhysicalObject; institutionCode: CEIOC; basisOfRecord: PreservedSpecimen
- **Type status:** Other material. **Occurrence:** recordNumber: PCC114; recordedBy: M. M. Silva, A. J. Mayhé Nunes, J. C. Santos, I. R. S. Cordeiro; individualCount: 2; sex: female; lifeStage: adult; occurrenceID: 6D42D8FA-6069-5F4C-9E18-5299BA8A2B72; **Taxon:** genus: Acromyrmex; specificEpithet: molestans; taxonRank: species; scientificNameAuthorship: Santschi, 1925; **Location:** continent: South America; country: Brazil; countryCode: BR; stateProvince: Rio de Janeiro; municipality: Rio de Janeiro; locality: Floresta da Tijuca, Parque Nacional da Tijuca, Trilha de Moutain bike (Açude da solidão).; verbatimCoordinates: 22 57 41.9S 43 17 24.0W; verbatimLatitude: 22 57 41.9S; verbatimLongitude: 43 17 24.0W; verbatimCoordinateSystem: degrees minutes seconds; verbatimSRS: WGS84; **Identification:** identificationID: Acromyrmexmolestans; identifiedBy: M. M. Silva; **Event:** samplingProtocol: Pitfall; year: 2022; month: 1; day: 21-23; verbatimEventDate: Summer 2022; habitat: Atlantic Forest; **Record Level:** type: PhysicalObject; institutionCode: CEIOC; basisOfRecord: PreservedSpecimen

##### Distribution

Endemic from Brazil.

##### Notes

This is the first record for the PNT. First reported from the State of Rio de Janeiro by Santschi (1937).

#### Acromyrmex subterraneus

(Forel, 1912)

4FC67FA7-9CAE-5932-A6BB-2CB43E465FD7

##### Materials

- **Type status:** Other material. **Occurrence:** recordNumber: WAC110; recordedBy: M. M. Silva, A. J. Mayhé Nunes, J. C. Santos, I. R. S. Cordeiro; individualCount: 2; sex: female; lifeStage: adult; occurrenceID: 25A7C37F-0039-5457-9379-C09329109724; **Taxon:** genus: Acromyrmex; specificEpithet: subterraneus; taxonRank: species; scientificNameAuthorship: (Forel, 1912); **Location:** continent: South America; country: Brazil; countryCode: BR; stateProvince: Rio de Janeiro; municipality: Rio de Janeiro; locality: Floresta da Tijuca, Parque Nacional da Tijuca, Bom Retiro (trilha para o pico da Tijuca); verbatimCoordinates: 22 56 46.2S 43 17 32.7W; verbatimLatitude: 22 56 46.2S; verbatimLongitude: 43 17 32.7W; verbatimCoordinateSystem: degrees minutes seconds; verbatimSRS: WGS84; **Identification:** identificationID: Acromyrmexsubterraneus; identifiedBy: M. M. Silva; **Event:** samplingProtocol: Winckler; year: 2022; month: 1; day: 17-19; verbatimEventDate: Summer 2022; habitat: Atlantic Forest; **Record Level:** type: PhysicalObject; institutionCode: CEIOC; basisOfRecord: PreservedSpecimen
- **Type status:** Other material. **Occurrence:** recordNumber: PBC110; recordedBy: M. M. Silva, A. J. Mayhé Nunes, J. C. Santos, I. R. S. Cordeiro; individualCount: 2; sex: female; lifeStage: adult; occurrenceID: F434B81B-43DA-54D4-8DA1-FA710299B16E; **Taxon:** genus: Acromyrmex; specificEpithet: subterraneus; taxonRank: species; scientificNameAuthorship: (Forel, 1912); **Location:** continent: South America; country: Brazil; countryCode: BR; stateProvince: Rio de Janeiro; municipality: Rio de Janeiro; locality: Floresta da Tijuca, Parque Nacional da Tijuca, trilha (caminho dos picos/ camminho da solidão); verbatimCoordinates: 22 57 23.8S 43 17 00.5W; verbatimLatitude: 22 57 23.8S; verbatimLongitude: 43 17 00.5W; verbatimCoordinateSystem: degrees minutes seconds; verbatimSRS: WGS84; **Identification:** identificationID: Acromyrmexsubterraneus; identifiedBy: M. M. Silva; **Event:** samplingProtocol: Winckler; year: 2022; month: 1; day: 17-21; verbatimEventDate: Summer 2022; habitat: Atlantic Forest; **Record Level:** type: PhysicalObject; institutionCode: CEIOC; basisOfRecord: PreservedSpecimen
- **Type status:** Other material. **Occurrence:** recordNumber: PCC110; recordedBy: M. M. Silva, A. J. Mayhé Nunes, J. C. Santos, I. R. S. Cordeiro; individualCount: 4; sex: female; lifeStage: adult; occurrenceID: 8E1DB942-30BD-5E2E-8F1D-861B5F8C665B; **Taxon:** genus: Acromyrmex; specificEpithet: subterraneus; taxonRank: species; scientificNameAuthorship: (Forel, 1912); **Location:** continent: South America; country: Brazil; countryCode: BR; stateProvince: Rio de Janeiro; municipality: Rio de Janeiro; locality: Floresta da Tijuca, Parque Nacional da Tijuca, Trilha de Moutain bike (Açude da solidão).; verbatimCoordinates: 22 57 41.2S 43 17 24.3W; verbatimLatitude: 22 57 41.2S; verbatimLongitude: 43 17 24.3W; verbatimCoordinateSystem: degrees minutes seconds; verbatimSRS: WGS84; **Identification:** identificationID: Acromyrmexsubterraneus; identifiedBy: M. M. Silva; **Event:** samplingProtocol: Pitfall; year: 2022; month: 1; day: 21-23; verbatimEventDate: Summer 2022; habitat: Atlantic Forest; **Record Level:** type: PhysicalObject; institutionCode: CEIOC; basisOfRecord: PreservedSpecimen

##### Distribution

Argentina, Bolivia, Brazil, Colombia, French Guiana, Paraguay, Peru, Venezuela.

##### Notes

This is the first record for the PNT. First record from the State of Rio de Janeiro by Gonçalves (1961).

#### Apterostigma ierense

Weber, 1937

01AFD404-E9E9-5508-A972-B13CEFFF418F

##### Materials

- **Type status:** Other material. **Occurrence:** recordNumber: WCC115; recordedBy: M. M. Silva, A. J. Mayhé Nunes, J. C. Santos, I. R. S. Cordeiro; individualCount: 1; sex: female; lifeStage: adult; occurrenceID: 05BD488F-BDAB-5C4A-B099-2B1F18CF71B5; **Taxon:** genus: Apterostigma; specificEpithet: ierense; taxonRank: species; scientificNameAuthorship: Weber, 1937; **Location:** continent: South America; country: Brazil; countryCode: BR; stateProvince: Rio de Janeiro; municipality: Rio de Janeiro; locality: Floresta da Tijuca, Parque Nacional da Tijuca, Bom Retiro (trilha para o pico da Tijuca); verbatimCoordinates: 22 56 46.2S 43 17 32.7W; verbatimLatitude: 22 56 46.2S; verbatimLongitude: 43 17 32.7W; verbatimCoordinateSystem: degrees minutes seconds; verbatimSRS: WGS84; **Identification:** identificationID: Apterostigmaierense; identifiedBy: M. M. Silva; **Event:** samplingProtocol: Winckler; year: 2022; month: 1; day: 21-23; verbatimEventDate: Summer 2022; habitat: Atlantic Forest; **Record Level:** type: PhysicalObject; institutionCode: CEIOC; basisOfRecord: PreservedSpecimen

##### Distribution

Bolivia, Brazil, Colombia, Ecuador, French Guiana, Panama, Peru, Trinidad and Tobago, Venezuela.

##### Notes

This is the first record for the State of Rio de Janeiro, filling a gap between Bahia to the north and São Paulo to the south.

#### Apterostigma pilosum

Mayr, 1865

56179297-B029-51BE-9F14-67791691E3CC

##### Materials

- **Type status:** Other material. **Occurrence:** recordNumber: WAS104; recordedBy: M. M. Silva, A. J. Mayhé Nunes, J. C. Santos, I. R. S. Cordeiro; individualCount: 7; sex: female; lifeStage: adult; occurrenceID: CFBA32E8-2BE8-584D-BF37-37AF4F9079B2; **Taxon:** genus: Apterostigma; specificEpithet: pilosum; taxonRank: species; scientificNameAuthorship: Mayr, 1865; **Location:** continent: South America; country: Brazil; countryCode: BR; stateProvince: Rio de Janeiro; municipality: Rio de Janeiro; locality: Floresta da Tijuca, Parque Nacional da Tijuca, Bom Retiro (trilha para o pico da Tijuca); verbatimCoordinates: 22 56 48.2S 43 17 29.8W; verbatimLatitude: 22 56 48.2S; verbatimLongitude: 43 17 29.8W; verbatimCoordinateSystem: degrees minutes seconds; verbatimSRS: WGS84; **Identification:** identificationID: Apterostigmapilosum; identifiedBy: M. M. Silva; **Event:** samplingProtocol: Winckler; year: 2021; month: 7; day: 19-21; verbatimEventDate: Winter 2021; habitat: Atlantic Forest; **Record Level:** type: PhysicalObject; institutionCode: CEIOC; basisOfRecord: PreservedSpecimen
- **Type status:** Other material. **Occurrence:** recordNumber: PBS105; recordedBy: M. M. Silva, A. J. Mayhé Nunes, J. C. Santos, I. R. S. Cordeiro; individualCount: 7; sex: female; lifeStage: adult; occurrenceID: C4C00D05-4518-5EFC-BEEE-9B89B26C7926; **Taxon:** genus: Apterostigma; specificEpithet: pilosum; taxonRank: species; scientificNameAuthorship: Mayr, 1865; **Location:** continent: South America; country: Brazil; countryCode: BR; stateProvince: Rio de Janeiro; municipality: Rio de Janeiro; locality: Floresta da Tijuca, Parque Nacional da Tijuca, trilha (caminho dos picos/ camminho da solidão); verbatimCoordinates: 22 57 23.2S 43 16 57.7W; verbatimLatitude: 22 57 23.2S; verbatimLongitude: 43 16 57.7W; verbatimCoordinateSystem: degrees minutes seconds; verbatimSRS: WGS84; **Identification:** identificationID: Apterostigmapilosum; identifiedBy: M. M. Silva; **Event:** samplingProtocol: Pitfall; year: 2021; month: 7; day: 21-23; verbatimEventDate: Winter 2021; habitat: Atlantic Forest; **Record Level:** type: PhysicalObject; institutionCode: CEIOC; basisOfRecord: PreservedSpecimen
- **Type status:** Other material. **Occurrence:** recordNumber: WCS119; recordedBy: M. M. Silva, A. J. Mayhé Nunes, J. C. Santos, I. R. S. Cordeiro; individualCount: 6; sex: female; lifeStage: adult; occurrenceID: 77B1442F-3414-5A42-95D4-74743F5A8921; **Taxon:** genus: Apterostigma; specificEpithet: pilosum; taxonRank: species; scientificNameAuthorship: Mayr, 1865; **Location:** continent: South America; country: Brazil; countryCode: BR; stateProvince: Rio de Janeiro; municipality: Rio de Janeiro; locality: Floresta da Tijuca, Parque Nacional da Tijuca, Trilha de Moutain bike (Açude da solidão).; verbatimCoordinates: 22 57 39.8S 43 17 30.9W; verbatimLatitude: 22 57 39.8S; verbatimLongitude: 43 17 30.9W; verbatimCoordinateSystem: degrees minutes seconds; verbatimSRS: WGS84; **Identification:** identificationID: Apterostigmapilosum; identifiedBy: M. M. Silva; **Event:** samplingProtocol: Winckler; year: 2021; month: 7; day: 23-25; verbatimEventDate: Winter 2021; habitat: Atlantic Forest; **Record Level:** type: PhysicalObject; institutionCode: CEIOC; basisOfRecord: PreservedSpecimen

##### Distribution

Argentina, Belize, Brazil, Colombia, Costa Rica, French Guiana, Guatemala, Guyana, Honduras, Mexico, Nicaragua, Panama, Paraguay, Peru, Trinidad and Tobago, Venezuela.

##### Notes

This is the first record for PNT. Previously recorded by Kempf (1972) and Brandão (1991) from the State of Rio de Janeiro without details.

#### Basiceros disciger

(Mayr, 1887)

7D646B52-4409-582D-A209-91D12FD5341C

##### Materials

- **Type status:** Other material. **Occurrence:** recordNumber: WAS113; recordedBy: M. M. Silva, A. J. Mayhé Nunes, J. C. Santos, I. R. S. Cordeiro; individualCount: 2; sex: female; lifeStage: adult; occurrenceID: B44371AF-FBC2-59F7-81F2-66048013B057; **Taxon:** genus: Basiceros; specificEpithet: disciger; taxonRank: species; scientificNameAuthorship: (Mayr, 1887); **Location:** continent: South America; country: Brazil; countryCode: BR; stateProvince: Rio de Janeiro; municipality: Rio de Janeiro; locality: Floresta da Tijuca, Parque Nacional da Tijuca, Bom Retiro (trilha para o pico da Tijuca); verbatimCoordinates: 22 56 48.5S 43 17 35.3W; verbatimLatitude: 22 56 48.5S; verbatimLongitude: 43 17 35.3W; verbatimCoordinateSystem: degrees minutes seconds; verbatimSRS: WGS84; **Identification:** identificationID: Basicerosdisciger; identifiedBy: M. M. Silva; **Event:** samplingProtocol: Winckler; year: 2021; month: 7; day: 19-21; verbatimEventDate: Winter 2021; habitat: Atlantic Forest; **Record Level:** type: PhysicalObject; institutionCode: CEIOC; basisOfRecord: PreservedSpecimen

##### Distribution

Argentina, Bolivia, Brazil, Colombia, Ecuador, Paraguay, Peru, Venezuela. The record from Costa Rica needs verification.

##### Notes

This is the first record for the Floresta da Tijuca sector. Previously recorded by Vargas et al. (2013) from the Reserva Florestal da Vista Chinesa, within the Serra da Carioca sector.

#### Cardiocondyla minutior

Forel, 1899

C67DF67C-FD99-5085-A1E5-F6557200E323

##### Materials

- **Type status:** Other material. **Occurrence:** recordNumber: WAC103; recordedBy: M. M. Silva, A. J. Mayhé Nunes, J. C. Santos, I. R. S. Cordeiro; individualCount: 2; sex: female; lifeStage: adult; occurrenceID: C4D3254B-1CF7-5634-8B6E-786C6715E91E; **Taxon:** genus: Cardiocondyla; specificEpithet: minutior; taxonRank: species; scientificNameAuthorship: Forel, 1899; **Location:** continent: South America; country: Brazil; countryCode: BR; stateProvince: Rio de Janeiro; municipality: Rio de Janeiro; locality: Floresta da Tijuca, Parque Nacional da Tijuca, Bom Retiro (trilha para o pico da Tijuca); verbatimCoordinates: 22 56 48.3S 43 17 32.2W; verbatimLatitude: 22 56 48.3S; verbatimLongitude: 43 17 32.2W; verbatimCoordinateSystem: degrees minutes seconds; verbatimSRS: WGS84; **Identification:** identificationID: Cardiocondylaminutior; identifiedBy: M. M. Silva; **Event:** samplingProtocol: Winckler; year: 2021; month: 1; day: 17-19; verbatimEventDate: Summer 2022; habitat: Atlantic Forest; **Record Level:** type: PhysicalObject; institutionCode: CEIOC; basisOfRecord: PreservedSpecimen

##### Native status

Exotic (Brazil)

##### Distribution

Native in the Oriental Region (Andaman and Nicobar Islands, continental India, Nepal, Sri Lanka, Vietnam). Exotic in the Australian, Madagascan, Nearctic, Neotropical, Oceanian, Panamanian, Saharo-Arabian and Sino-Japanese Regions, including Brazil.

##### Notes

This is the first record for the State of Rio de Janeiro, filling a gap between Minas Gerais and Espírito Santo to the north and Santa Catarina to the south.

#### Carebara brevipilosa

Fernández, 2004

8405C805-582B-564D-BDB5-32FEB63B1129

##### Materials

- **Type status:** Other material. **Occurrence:** recordNumber: WBS109; recordedBy: M. M. Silva, A. J. Mayhé Nunes, J. C. Santos, I. R. S. Cordeiro; individualCount: 4; sex: female; lifeStage: adult; occurrenceID: F7C529EC-C5E0-5799-BB5C-7A26F3F04E02; **Taxon:** genus: Carebara; specificEpithet: brevipilosa; taxonRank: species; scientificNameAuthorship: Fernández, 2004; **Location:** continent: South America; country: Brazil; countryCode: BR; stateProvince: Rio de Janeiro; municipality: Rio de Janeiro; locality: Floresta da Tijuca, Parque Nacional da Tijuca, trilha (caminho dos picos/ camminho da solidão); verbatimCoordinates: 22 57 25.3S 43 16 56.2W; verbatimLatitude: 22 57 25.3S; verbatimLongitude: 43 16 56.2W; verbatimCoordinateSystem: degrees minutes seconds; verbatimSRS: WGS84; **Identification:** identificationID: Carebarabrevipilosa; identifiedBy: M. M. Silva; **Event:** samplingProtocol: Winckler; year: 2021; month: 7; day: 21-23; verbatimEventDate: Winter 2021; habitat: Atlantic Forest; **Record Level:** type: PhysicalObject; institutionCode: CEIOC; basisOfRecord: PreservedSpecimen

##### Distribution

Argentina, Brazil, Colombia, Costa Rica, Ecuador, El Salvador, French Guiana, Guyana, Honduras, Mexico, Nicaragua, Panama, Suriname, Venezuela.

##### Notes

This is the first record for PNT. This species was recorded by de Oliveira et al. (2018) from another protected area in the State of Rio de Janeiro.

#### Crematogaster montana

Borgmeier, 1939

7D2F0389-6B63-56ED-81E5-6FC21D0C0D60

##### Materials

- **Type status:** Other material. **Occurrence:** recordNumber: PAC119; recordedBy: M. M. Silva, A. J. Mayhé Nunes, J. C. Santos, I. R. S. Cordeiro; individualCount: 1; sex: female; lifeStage: adult; occurrenceID: EDD0B456-3F7A-56C6-AE01-93F7C2CEB6F1; **Taxon:** genus: Crematogaster; specificEpithet: montana; taxonRank: species; scientificNameAuthorship: Borgmeier, 1939; **Location:** continent: South America; country: Brazil; countryCode: BR; stateProvince: Rio de Janeiro; municipality: Rio de Janeiro; locality: Floresta da Tijuca, Parque Nacional da Tijuca, Bom Retiro (trilha para o pico da Tijuca); verbatimCoordinates: 22 56 48.1S 43 17 31.7W; verbatimLatitude: 22 56 48.1S; verbatimLongitude: 43 17 31.7W; verbatimCoordinateSystem: degrees minutes seconds; verbatimSRS: WGS84; **Identification:** identificationID: Crematogastermontana; identifiedBy: M. M. Silva; **Event:** samplingProtocol: Pitfall; year: 2022; month: 1; day: 17-19; verbatimEventDate: Summer 2022; habitat: Atlantic Forest; **Record Level:** type: PhysicalObject; institutionCode: CEIOC; basisOfRecord: PreservedSpecimen

##### Distribution

Endemic from the State of Rio de Janeiro.

##### Notes

This is the first record for PNT. Previously known only from the Municipality of Petrópolis, located approximately 70 km north of Rio de Janeiro.

#### Crematogaster nigropilosa

Mayr, 1870

0D473719-8F1D-5FB8-99B1-61E0AFC6F359

##### Materials

- **Type status:** Other material. **Occurrence:** recordNumber: WCS115; recordedBy: M. M. Silva, A. J. Mayhé Nunes, J. C. Santos, I. R. S. Cordeiro; individualCount: 1; sex: female; lifeStage: adult; occurrenceID: 1544B139-8F9C-5DC5-94C6-3DF538CB2725; **Taxon:** genus: Crematogaster; specificEpithet: nigropilosa; taxonRank: species; scientificNameAuthorship: Mayr, 1870; **Location:** continent: South America; country: Brazil; countryCode: BR; stateProvince: Rio de Janeiro; municipality: Rio de Janeiro; locality: Floresta da Tijuca, Parque Nacional da Tijuca, Trilha de Moutain bike (Açude da solidão).; verbatimCoordinates: 22 57 40.8S 43 17 29.2W; verbatimLatitude: 22 57 40.8S; verbatimLongitude: 43 17 29.2W; verbatimCoordinateSystem: degrees minutes seconds; verbatimSRS: WGS84; **Identification:** identificationID: Crematogasternigropilosa; identifiedBy: M. M. Silva; **Event:** samplingProtocol: Winckler; year: 2021; month: 7; day: 23-25; verbatimEventDate: Winter 2021; habitat: Atlantic Forest; **Record Level:** type: PhysicalObject; institutionCode: CEIOC; basisOfRecord: PreservedSpecimen

##### Distribution

Argentina, Bolivia, Brazil, Colombia, Costa Rica, Ecuador, French Guiana, Guatemala, Guyana, Honduras, Lesser Antilles, Mexico, Nicaragua, Panama, Paraguay, Peru, Trinidad and Tobago, Venezuela.

##### Notes

This is the first record for the Floresta da Tijuca sector. This species was recorded by Vargas et al. (2013) and Santos et al. (2019) from the Serra da Carioca sector.

#### Cyphomyrmex minutus

Mayr, 1862

235412BD-7464-5A71-946C-530A6096C689

##### Materials

- **Type status:** Other material. **Occurrence:** recordNumber: WAS101; recordedBy: M. M. Silva, A. J. Mayhé Nunes, J. C. Santos, I. R. S. Cordeiro; individualCount: 1; sex: female; lifeStage: adult; occurrenceID: 6F3E224A-F062-5823-8DD0-29B41338C71D; **Taxon:** genus: Cyphomyrmex; specificEpithet: minutus; taxonRank: species; scientificNameAuthorship: Mayr, 1862; **Location:** continent: South America; country: Brazil; countryCode: BR; stateProvince: Rio de Janeiro; municipality: Rio de Janeiro; locality: Floresta da Tijuca, Parque Nacional da Tijuca, Bom Retiro (trilha para o pico da Tijuca); verbatimCoordinates: 22 56 47.5S 43 17 31.2W; verbatimLatitude: 22 56 47.5S; verbatimLongitude: 43 17 31.2W; verbatimCoordinateSystem: degrees minutes seconds; verbatimSRS: WGS84; **Identification:** identificationID: Cyphomyrmexminutus; identifiedBy: M. M. Silva; **Event:** samplingProtocol: Winckler; year: 2021; month: 7; day: 19-21; verbatimEventDate: Winter 2021; habitat: Atlantic Forest; **Record Level:** type: PhysicalObject; institutionCode: CEIOC; basisOfRecord: PreservedSpecimen
- **Type status:** Other material. **Occurrence:** recordNumber: WBS105; recordedBy: M. M. Silva, A. J. Mayhé Nunes, J. C. Santos, I. R. S. Cordeiro; individualCount: 2; sex: female; lifeStage: adult; occurrenceID: F1F80378-A91E-5316-97B0-A88B2E1334B3; **Taxon:** genus: Cyphomyrmex; specificEpithet: minutus; taxonRank: species; scientificNameAuthorship: Mayr, 1862; **Location:** continent: South America; country: Brazil; countryCode: BR; stateProvince: Rio de Janeiro; municipality: Rio de Janeiro; locality: Floresta da Tijuca, Parque Nacional da Tijuca, trilha (caminho dos picos/ camminho da solidão); verbatimCoordinates: 22 57 23.8S 43 16 57.7W; verbatimLatitude: 22 57 23.8S; verbatimLongitude: 43 16 57.7W; verbatimCoordinateSystem: degrees minutes seconds; verbatimSRS: WGS84; **Identification:** identificationID: Cyphomyrmexminutus; identifiedBy: M. M. Silva; **Event:** samplingProtocol: Winckler; year: 2021; month: 7; day: 21-23; verbatimEventDate: Winter 2021; habitat: Atlantic Forest; **Record Level:** type: PhysicalObject; institutionCode: CEIOC; basisOfRecord: PreservedSpecimen
- **Type status:** Other material. **Occurrence:** recordNumber: WCS101; recordedBy: M. M. Silva, A. J. Mayhé Nunes, J. C. Santos, I. R. S. Cordeiro; individualCount: 4; sex: female; lifeStage: adult; occurrenceID: 748FF94B-9E05-56F7-89C0-C070E256356F; **Taxon:** genus: Cyphomyrmex; specificEpithet: minutus; taxonRank: species; scientificNameAuthorship: Mayr, 1862; **Location:** continent: South America; country: Brazil; countryCode: BR; stateProvince: Rio de Janeiro; municipality: Rio de Janeiro; locality: Floresta da Tijuca, Parque Nacional da Tijuca, Trilha de Moutain bike (Açude da solidão).; verbatimCoordinates: 22 57 42.4S 43 17 23.8W; verbatimLatitude: 22 57 42.4S; verbatimLongitude: 43 17 23.8W; verbatimCoordinateSystem: degrees minutes seconds; verbatimSRS: WGS84; **Identification:** identificationID: Cyphomyrmexminutus; identifiedBy: M. M. Silva; **Event:** samplingProtocol: Winckler; year: 2021; month: 7; day: 23-25; verbatimEventDate: Winter 2021; habitat: Atlantic Forest; **Record Level:** type: PhysicalObject; institutionCode: CEIOC; basisOfRecord: PreservedSpecimen

##### Distribution

Argentina, Bahamas, Bolivia, Brazil, Colombia, Costa Rica, Cuba, French Guiana, Guatemala, Guyana, Hispaniola, Honduras, Jamaica, Lesser Antilles, Mexico, Panama, Paraguay, Peru, Puerto Rico, Suriname, Trinidad and Tobago, United States, Venezuela.

##### Notes

This is the first record for the Floresta da Tijuca sector. Previously recorded by Vargas et al. (2013) from the Reserva Florestal da Vista Chinesa, within the Serra da Carioca sector.

#### Cyphomyrmex rimosus

(Spinola, 1851)

7800C38E-39BC-5DBD-AF74-4199E87D2997

##### Materials

- **Type status:** Other material. **Occurrence:** recordNumber: PAC101; recordedBy: M. M. Silva, A. J. Mayhé Nunes, J. C. Santos, I. R. S. Cordeiro; individualCount: 3; sex: female; lifeStage: adult; occurrenceID: 235A9FCF-95A8-53CF-A9B7-01810A750007; **Taxon:** genus: Cyphomyrmex; specificEpithet: rimosus; taxonRank: species; scientificNameAuthorship: (Spinola, 1851); **Location:** continent: South America; country: Brazil; countryCode: BR; stateProvince: Rio de Janeiro; municipality: Rio de Janeiro; locality: Floresta da Tijuca, Parque Nacional da Tijuca, Bom Retiro (trilha para o pico da Tijuca); verbatimCoordinates: 22 56 46.3S 43 17 30.3W; verbatimLatitude: 22 56 46.3S; verbatimLongitude: 43 17 30.3W; verbatimCoordinateSystem: degrees minutes seconds; verbatimSRS: WGS84; **Identification:** identificationID: Cyphomyrmexrimosus; identifiedBy: M. M. Silva; **Event:** samplingProtocol: Pitfall; year: 2021; month: 1; day: 17-19; verbatimEventDate: Summer 2021; habitat: Atlantic Forest; **Record Level:** type: PhysicalObject; institutionCode: CEIOC; basisOfRecord: PreservedSpecimen
- **Type status:** Other material. **Occurrence:** recordNumber: WBS105; recordedBy: M. M. Silva, A. J. Mayhé Nunes, J. C. Santos, I. R. S. Cordeiro; individualCount: 4; sex: female; lifeStage: adult; occurrenceID: 06DC9D8E-5887-501B-8FB7-F460C3566D27; **Taxon:** genus: Cyphomyrmex; specificEpithet: rimosus; taxonRank: species; scientificNameAuthorship: (Spinola, 1851); **Location:** continent: South America; country: Brazil; countryCode: BR; stateProvince: Rio de Janeiro; municipality: Rio de Janeiro; locality: Floresta da Tijuca, Parque Nacional da Tijuca, trilha (caminho dos picos/ camminho da solidão); verbatimCoordinates: 22 57 23.8S 43 16 57.7W; verbatimLatitude: 22 57 23.8S; verbatimLongitude: 43 16 57.7W; verbatimCoordinateSystem: degrees minutes seconds; verbatimSRS: WGS84; **Identification:** identificationID: Cyphomyrmexrimosus; identifiedBy: M. M. Silva; **Event:** samplingProtocol: Winckler; year: 2021; month: 7; day: 21-23; verbatimEventDate: Winter 2021; habitat: Atlantic Forest; **Record Level:** type: PhysicalObject; institutionCode: CEIOC; basisOfRecord: PreservedSpecimen
- **Type status:** Other material. **Occurrence:** recordNumber: WCS102; recordedBy: M. M. Silva, A. J. Mayhé Nunes, J. C. Santos, I. R. S. Cordeiro; individualCount: 11; sex: female; lifeStage: adult; occurrenceID: EACE329D-5FD3-5920-B991-82CFB8BB8C18; **Taxon:** genus: Cyphomyrmex; specificEpithet: rimosus; taxonRank: species; scientificNameAuthorship: (Spinola, 1851); **Location:** continent: South America; country: Brazil; countryCode: BR; stateProvince: Rio de Janeiro; municipality: Rio de Janeiro; locality: Floresta da Tijuca, Parque Nacional da Tijuca, Trilha de Moutain bike (Açude da solidão).; verbatimCoordinates: 22 57 42.0S 43 17 23.9W; verbatimLatitude: 22 57 42.0S; verbatimLongitude: 43 17 23.9W; verbatimCoordinateSystem: degrees minutes seconds; verbatimSRS: WGS84; **Identification:** identificationID: Cyphomyrmexrimosus; identifiedBy: M. M. Silva; **Event:** samplingProtocol: Winckler; year: 2021; month: 7; day: 23-25; verbatimEventDate: Winter 2021; habitat: Atlantic Forest; **Record Level:** type: PhysicalObject; institutionCode: CEIOC; basisOfRecord: PreservedSpecimen

##### Distribution

Argentina, Belize, Bolivia, Brazil, Colombia, Costa Rica, Cuba, Ecuador, French Guiana, Guatemala, Guyana, Hispaniola, Honduras, Lesser Antilles, Mexico, Nicaragua, Panama, Paraguay, Peru, Puerto Rico, Suriname, Trinidad and Tobago, Uruguay, Venezuela. Exotic in the Galápagos Islands and southeast and southwest United States. Introduced in Hawaii. Records from the States of Arizona, California and Michigan (United States) are dubious.

##### Notes

This is the first record for the Floresta da Tijuca sector. This species was recorded by Santos et al. (2019) from the Rio de Janeiro Botanical Garden and the Parque Lage, within the Serra da Carioca sector.

#### Hylomyrma reitteri

(Mayr, 1887)

8DF34435-3C8F-5FAD-9BAD-3A9810FB1207

##### Materials

- **Type status:** Other material. **Occurrence:** recordNumber: WAS102; recordedBy: M. M. Silva, A. J. Mayhé Nunes, J. C. Santos, I. R. S. Cordeiro; individualCount: 11; sex: female; lifeStage: adult; occurrenceID: E9AE839C-A942-55EA-91B7-88123A3E9C4C; **Taxon:** genus: Hylomyrma; specificEpithet: reitteri; taxonRank: species; scientificNameAuthorship: (Mayr, 1887); **Location:** continent: South America; country: Brazil; countryCode: BR; stateProvince: Rio de Janeiro; municipality: Rio de Janeiro; locality: Floresta da Tijuca, Parque Nacional da Tijuca, Bom Retiro (trilha para o pico da Tijuca); verbatimCoordinates: 22 56 47.5S 43 17 30.6W; verbatimLatitude: 22 56 47.5S; verbatimLongitude: 43 17 30.6W; verbatimCoordinateSystem: degrees minutes seconds; verbatimSRS: WGS84; **Identification:** identificationID: Hylomyrmareitteri; identifiedBy: M. M. Silva; **Event:** samplingProtocol: Winckler; year: 2021; month: 7; day: 19-21; verbatimEventDate: Winter 2021; habitat: Atlantic Forest; **Record Level:** type: PhysicalObject; institutionCode: CEIOC; basisOfRecord: PreservedSpecimen
- **Type status:** Other material. **Occurrence:** recordNumber: WBS105; recordedBy: M. M. Silva, A. J. Mayhé Nunes, J. C. Santos, I. R. S. Cordeiro; individualCount: 11; sex: female; lifeStage: adult; occurrenceID: 87A7A64C-7359-5B57-8C46-1AA835FA2ADC; **Taxon:** genus: Hylomyrma; specificEpithet: reitteri; taxonRank: species; scientificNameAuthorship: (Mayr, 1887); **Location:** continent: South America; country: Brazil; countryCode: BR; stateProvince: Rio de Janeiro; municipality: Rio de Janeiro; locality: Floresta da Tijuca, Parque Nacional da Tijuca, trilha (caminho dos picos/ camminho da solidão); verbatimCoordinates: 22 57 23.8S 43 16 57.7W; verbatimLatitude: 22 57 23.8S; verbatimLongitude: 43 16 57.7W; verbatimCoordinateSystem: degrees minutes seconds; verbatimSRS: WGS84; **Identification:** identificationID: Hylomyrmareitteri; identifiedBy: M. M. Silva; **Event:** samplingProtocol: Winckler; year: 2021; month: 7; day: 21-23; verbatimEventDate: Winter 2021; habitat: Atlantic Forest; **Record Level:** type: PhysicalObject; institutionCode: CEIOC; basisOfRecord: PreservedSpecimen
- **Type status:** Other material. **Occurrence:** recordNumber: WCS102; recordedBy: M. M. Silva, A. J. Mayhé Nunes, J. C. Santos, I. R. S. Cordeiro; individualCount: 10; sex: female; lifeStage: adult; occurrenceID: F8BE1B98-456B-53B6-94F6-3E3FE80207B3; **Taxon:** genus: Hylomyrma; specificEpithet: reitteri; taxonRank: species; scientificNameAuthorship: (Mayr, 1887); **Location:** continent: South America; country: Brazil; countryCode: BR; stateProvince: Rio de Janeiro; municipality: Rio de Janeiro; locality: Floresta da Tijuca, Parque Nacional da Tijuca, Trilha de Moutain bike (Açude da solidão).; verbatimCoordinates: 22 57 42.0S 43 17 23.9W; verbatimLatitude: 22 57 42.0S; verbatimLongitude: 43 17 23.9W; verbatimCoordinateSystem: degrees minutes seconds; verbatimSRS: WGS84; **Identification:** identificationID: Hylomyrmareitteri; identifiedBy: M. M. Silva; **Event:** samplingProtocol: Winckler; year: 2021; month: 7; day: 23-25; verbatimEventDate: Winter 2021; habitat: Atlantic Forest; **Record Level:** type: PhysicalObject; institutionCode: CEIOC; basisOfRecord: PreservedSpecimen

##### Distribution

Argentina, Brazil, Colombia, Paraguay, Venezuela.

##### Notes

Kempf (1960) recorded this species from "Floresta da Tijuca" without details.

#### Lachnomyrmex plaumanni

Borgmeier, 1957

1DE09B0C-503C-5F03-9C8F-C52DF10F055B

##### Materials

- **Type status:** Other material. **Occurrence:** recordNumber: WAS104; recordedBy: M. M. Silva, A. J. Mayhé Nunes, J. C. Santos, I. R. S. Cordeiro; individualCount: 1; sex: female; lifeStage: adult; occurrenceID: 0ED888EB-36F7-55D0-B472-F1D1478B2258; **Taxon:** genus: Lachnomyrmex; specificEpithet: plaumanni; taxonRank: species; scientificNameAuthorship: Borgmeier, 1957; **Location:** continent: South America; country: Brazil; countryCode: BR; stateProvince: Rio de Janeiro; municipality: Rio de Janeiro; locality: Floresta da Tijuca, Parque Nacional da Tijuca, Bom Retiro (trilha para o pico da Tijuca); verbatimCoordinates: 22 56 48.2S 43 17 29.8W; verbatimLatitude: 22 56 48.2S; verbatimLongitude: 43 17 29.8W; verbatimCoordinateSystem: degrees minutes seconds; verbatimSRS: WGS84; **Identification:** identificationID: Lachnomyrmexplaumanni; identifiedBy: M. M. Silva; **Event:** samplingProtocol: Winckler; year: 2021; month: 7; day: 19-21; verbatimEventDate: Winter 2021; habitat: Atlantic Forest; **Record Level:** type: PhysicalObject; institutionCode: CEIOC; basisOfRecord: PreservedSpecimen

##### Distribution

Argentina, Brazil. The record from the State of Pará (Brazil) is dubious, while those from Costa Rica need verification.

##### Notes

This is the first record for the PNT. This species was recorded by Feitosa and Brandão (2008) and Orsolon-Souza et al. (2011) from the Reserva Biológica do Tinguá, another protected area in the State of Rio de Janeiro.

#### Megalomyrmex goeldii

Forel, 1912

F7F2FE05-357D-5DD9-AEDB-CA49B2B5CEFE

##### Materials

- **Type status:** Other material. **Occurrence:** recordNumber: WAC115; recordedBy: M. M. Silva, A. J. Mayhé Nunes, J. C. Santos, I. R. S. Cordeiro; individualCount: 8; sex: female; lifeStage: adult; occurrenceID: 57BA11A7-E51C-5E5D-B072-C5C127E2DA08; **Taxon:** genus: Megalomyrmex; specificEpithet: goeldii; taxonRank: species; scientificNameAuthorship: Forel, 1912; **Location:** continent: South America; country: Brazil; countryCode: BR; stateProvince: Rio de Janeiro; municipality: Rio de Janeiro; locality: Floresta da Tijuca, Parque Nacional da Tijuca, Bom Retiro (trilha para o pico da Tijuca); verbatimCoordinates: 22 56 47.7S 43 17 33.1W; verbatimLatitude: 22 56 47.7S; verbatimLongitude: 43 17 33.1W; verbatimCoordinateSystem: degrees minutes seconds; verbatimSRS: WGS84; **Identification:** identificationID: Megalomyrmexgoeldii; identifiedBy: M. M. Silva; **Event:** samplingProtocol: Winckler; year: 2022; month: 1; day: 17-19; verbatimEventDate: Summer 2022; habitat: Atlantic Forest; **Record Level:** type: PhysicalObject; institutionCode: CEIOC; basisOfRecord: PreservedSpecimen
- **Type status:** Other material. **Occurrence:** recordNumber: WBS116; recordedBy: M. M. Silva, A. J. Mayhé Nunes, J. C. Santos, I. R. S. Cordeiro; individualCount: 20; sex: female; lifeStage: adult; occurrenceID: 4C1EE0B1-85CB-5EA8-911F-94898884BF38; **Taxon:** genus: Megalomyrmex; specificEpithet: goeldii; taxonRank: species; scientificNameAuthorship: Forel, 1912; **Location:** continent: South America; country: Brazil; countryCode: BR; stateProvince: Rio de Janeiro; municipality: Rio de Janeiro; locality: Floresta da Tijuca, Parque Nacional da Tijuca, trilha (caminho dos picos/ camminho da solidão); verbatimCoordinates: 22 57 30.2S 43 16 57.17W; verbatimLatitude: 22 57 30.2S; verbatimLongitude: 43 16 57.17W; verbatimCoordinateSystem: degrees minutes seconds; verbatimSRS: WGS84; **Identification:** identificationID: Megalomyrmexgoeldii; identifiedBy: M. M. Silva; **Event:** samplingProtocol: Winckler; year: 2021; month: 7; day: 21-23; verbatimEventDate: Winter 2021; habitat: Atlantic Forest; **Record Level:** type: PhysicalObject; institutionCode: CEIOC; basisOfRecord: PreservedSpecimen
- **Type status:** Other material. **Occurrence:** recordNumber: WCS102; recordedBy: M. M. Silva, A. J. Mayhé Nunes, J. C. Santos, I. R. S. Cordeiro; individualCount: 15; sex: female; lifeStage: adult; occurrenceID: 1F7C5AF3-A562-56BB-A97D-5486968838F3; **Taxon:** genus: Megalomyrmex; specificEpithet: goeldii; taxonRank: species; scientificNameAuthorship: Forel, 1912; **Location:** continent: South America; country: Brazil; countryCode: BR; stateProvince: Rio de Janeiro; municipality: Rio de Janeiro; locality: Floresta da Tijuca, Parque Nacional da Tijuca, Trilha de Moutain bike (Açude da solidão).; verbatimCoordinates: 22 57 42.0S 43 17 23.9W; verbatimLatitude: 22 57 42.0S; verbatimLongitude: 43 17 23.9W; verbatimCoordinateSystem: degrees minutes seconds; verbatimSRS: WGS84; **Identification:** identificationID: Megalomyrmexgoeldii; identifiedBy: M. M. Silva; **Event:** samplingProtocol: Winckler; year: 2021; month: 7; day: 23-25; verbatimEventDate: Winter 2021; habitat: Atlantic Forest; **Record Level:** type: PhysicalObject; institutionCode: CEIOC; basisOfRecord: PreservedSpecimen

##### Distribution

Endemic from Brazil.

##### Notes

Brandão (1990) recorded this species from "Floresta da Tijuca" without details.

#### Mycetomoellerius oetkeri

(Forel, 1908)

DBC32356-8704-509F-A372-25A77C92E0E2

##### Materials

- **Type status:** Other material. **Occurrence:** recordNumber: PBS102; recordedBy: M. M. Silva, A. J. Mayhé Nunes, J. C. Santos, I. R. S. Cordeiro; individualCount: 5; sex: female; lifeStage: adult; occurrenceID: CF335390-014A-521F-BE45-EB20FC102C21; **Taxon:** genus: Mycetomoellerius; specificEpithet: oetkeri; taxonRank: species; scientificNameAuthorship: (Forel, 1908); **Location:** continent: South America; country: Brazil; countryCode: BR; stateProvince: Rio de Janeiro; municipality: Rio de Janeiro; locality: Floresta da Tijuca, Parque Nacional da Tijuca, trilha (caminho dos picos/ camminho da solidão); verbatimCoordinates: 22 57 26.2S 43 16 55.2W; verbatimLatitude: 22 57 26.2S; verbatimLongitude: 43 16 55.2W; verbatimCoordinateSystem: degrees minutes seconds; verbatimSRS: WGS84; **Identification:** identificationID: Mycetomoelleriusoetkeri; identifiedBy: M. M. Silva; **Event:** samplingProtocol: Pitfall; year: 2021; month: 7; day: 21-23; verbatimEventDate: Winter 2021; habitat: Atlantic Forest; **Record Level:** type: PhysicalObject; institutionCode: CEIOC; basisOfRecord: PreservedSpecimen

##### Distribution

Endemic from Brazil.

##### Notes

This is the first record for the PNT. This species was recorded by Kempf (1972) from the State of Rio de Janeiro without details.

#### Octostruma iheringi

(Emery, 1888)

6ABB3DE8-D500-5BB6-BD89-BA804E652C73

##### Materials

- **Type status:** Other material. **Occurrence:** recordNumber: WCS117; recordedBy: M. M. Silva, A. J. Mayhé Nunes, J. C. Santos, I. R. S. Cordeiro; individualCount: 1; sex: female; lifeStage: adult; occurrenceID: B0C4731D-4CD6-59AD-B72C-0C1F80F22E9E; **Taxon:** genus: Octostruma; specificEpithet: iheringi; taxonRank: species; scientificNameAuthorship: (Emery, 1888); **Location:** continent: South America; country: Brazil; countryCode: BR; stateProvince: Rio de Janeiro; municipality: Rio de Janeiro; locality: Floresta da Tijuca, Parque Nacional da Tijuca, Trilha de Moutain bike (Açude da solidão).; verbatimCoordinates: 22 57 40.5S 43 17 29.9W; verbatimLatitude: 22 57 40.5S; verbatimLongitude: 43 17 29.9W; verbatimCoordinateSystem: degrees minutes seconds; verbatimSRS: WGS84; **Identification:** identificationID: Octostrumaiheringi; identifiedBy: M. M. Silva; **Event:** samplingProtocol: Winckler; year: 2021; month: 7; day: 23-25; verbatimEventDate: Winter 2021; habitat: Atlantic Forest; **Record Level:** type: PhysicalObject; institutionCode: CEIOC; basisOfRecord: PreservedSpecimen

##### Distribution

Argentina, Bolivia, Brazil, Colombia, Costa Rica, Ecuador, French Guiana, Guatemala, Guyana, Honduras, Jamaica, Lesser Antilles, Mexico, Nicaragua, Panama, Paraguay, Peru, Suriname, Trinidad and Tobago, Venezuela. Records from the United States are dubious.

##### Notes

Brown and Kempf (1960) recorded this species from the "Floresta da Tijuca" without details.

#### Octostruma rugifera

(Mayr, 1887)

7D2BAE70-9F78-5767-929B-90F01633B477

##### Materials

- **Type status:** Other material. **Occurrence:** recordNumber: WAS113; recordedBy: M. M. Silva, A. J. Mayhé Nunes, J. C. Santos, I. R. S. Cordeiro; individualCount: 4; sex: female; lifeStage: adult; occurrenceID: 98B9AAA0-2B7E-5626-BDA4-142B65DC5354; **Taxon:** genus: Octostruma; specificEpithet: rugifera; taxonRank: species; scientificNameAuthorship: (Mayr, 1887); **Location:** continent: South America; country: Brazil; countryCode: BR; stateProvince: Rio de Janeiro; municipality: Rio de Janeiro; locality: Floresta da Tijuca, Parque Nacional da Tijuca, Bom Retiro (trilha para o pico da Tijuca); verbatimCoordinates: 22 56 48.5S 43 17 35.3W; verbatimLatitude: 22 56 48.5S; verbatimLongitude: 43 17 35.3W; verbatimCoordinateSystem: degrees minutes seconds; verbatimSRS: WGS84; **Identification:** identificationID: Octostrumarugifera; identifiedBy: M. M. Silva; **Event:** samplingProtocol: Winckler; year: 2021; month: 7; day: 19-21; verbatimEventDate: Winter 2021; habitat: Atlantic Forest; **Record Level:** type: PhysicalObject; institutionCode: CEIOC; basisOfRecord: PreservedSpecimen
- **Type status:** Other material. **Occurrence:** recordNumber: WBS102; recordedBy: M. M. Silva, A. J. Mayhé Nunes, J. C. Santos, I. R. S. Cordeiro; individualCount: 8; sex: female; lifeStage: adult; occurrenceID: 3BEF370A-42F7-5089-A38E-A2CEBF60F832; **Taxon:** genus: Octostruma; specificEpithet: rugifera; taxonRank: species; scientificNameAuthorship: (Mayr, 1887); **Location:** continent: South America; country: Brazil; countryCode: BR; stateProvince: Rio de Janeiro; municipality: Rio de Janeiro; locality: Floresta da Tijuca, Parque Nacional da Tijuca, trilha (caminho dos picos/ camminho da solidão); verbatimCoordinates: 22 57 24.0S 43 16 59.9W; verbatimLatitude: 22 57 24.0S; verbatimLongitude: 43 16 59.9W; verbatimCoordinateSystem: degrees minutes seconds; verbatimSRS: WGS84; **Identification:** identificationID: Octostrumarugifera; identifiedBy: M. M. Silva; **Event:** samplingProtocol: Winckler; year: 2021; month: 7; day: 21-23; verbatimEventDate: Winter 2021; habitat: Atlantic Forest; **Record Level:** type: PhysicalObject; institutionCode: CEIOC; basisOfRecord: PreservedSpecimen
- **Type status:** Other material. **Occurrence:** recordNumber: PCS106; recordedBy: M. M. Silva, A. J. Mayhé Nunes, J. C. Santos, I. R. S. Cordeiro; individualCount: 13; sex: female; lifeStage: adult; occurrenceID: 9F148668-130E-5FBE-A669-EAA2AE1FEBB9; **Taxon:** genus: Octostruma; specificEpithet: rugifera; taxonRank: species; scientificNameAuthorship: (Mayr, 1887); **Location:** continent: South America; country: Brazil; countryCode: BR; stateProvince: Rio de Janeiro; municipality: Rio de Janeiro; locality: Floresta da Tijuca, Parque Nacional da Tijuca, Trilha de Moutain bike (Açude da solidão).; verbatimCoordinates: 22 57 42.4S 43 17 24.5W; verbatimLatitude: 22 57 42.4S; verbatimLongitude: 43 17 24.5W; verbatimCoordinateSystem: degrees minutes seconds; verbatimSRS: WGS84; **Identification:** identificationID: Octostrumarugifera; identifiedBy: M. M. Silva; **Event:** samplingProtocol: Pitfall; year: 2021; month: 7; day: 23-25; verbatimEventDate: Winter 2021; habitat: Atlantic Forest; **Record Level:** type: PhysicalObject; institutionCode: CEIOC; basisOfRecord: PreservedSpecimen

##### Distribution

Argentina, Brazil, Colombia, Panama, Paraguay, San Andrés, Providencia and Santa Catalina, Venezuela.

##### Notes

Brown and Kempf (1960) recorded this species from "Floresta da Tijuca" without details.

#### Oxyepoecus myops

Albuquerque & Brandão, 2009

0985DF7D-6FE5-50CC-9FA4-D862A47D30F3

##### Materials

- **Type status:** Other material. **Occurrence:** recordNumber: WAC103; recordedBy: M. M. Silva, A. J. Mayhé Nunes, J. C. Santos, I. R. S. Cordeiro; individualCount: 1; sex: female; lifeStage: adult; occurrenceID: 3AA3134E-C79F-5FB8-8B46-0E9F9618D102; **Taxon:** genus: Oxyepoecus; specificEpithet: myops; taxonRank: species; scientificNameAuthorship: Albuquerque & Brandão, 2009; **Location:** continent: South America; country: Brazil; countryCode: BR; stateProvince: Rio de Janeiro; municipality: Rio de Janeiro; locality: Floresta da Tijuca, Parque Nacional da Tijuca, Bom Retiro (trilha para o pico da Tijuca); verbatimCoordinates: 22 56 48.3S 43 17 32.2W; verbatimLatitude: 22 56 48.3S; verbatimLongitude: 43 17 32.2W; verbatimCoordinateSystem: degrees minutes seconds; verbatimSRS: WGS84; **Identification:** identificationID: Oxyepoecusmyops; identifiedBy: M. M. Silva; **Event:** samplingProtocol: Winckler; year: 2022; month: 1; day: 17-19; verbatimEventDate: Summer 2022; habitat: Atlantic Forest; **Record Level:** type: PhysicalObject; institutionCode: CEIOC; basisOfRecord: PreservedSpecimen

##### Distribution

Endemic from Brazil.

##### Notes

This is the first record for PNT. This species was recorded by Albuquerque and Brandão (2009) and Lasmar et al. (2020) from other protected areas in the State of Rio de Janeiro, such as the Parque Nacional da Serra dos Órgãos and the Reserva Biológica do Tinguá.

#### Pheidole tijucana

Borgmeier, 1927

D7A454B9-AEAD-5B3D-94B1-163AB2AFA8E7

##### Materials

- **Type status:** Other material. **Occurrence:** recordNumber: PAC111; recordedBy: M. M. Silva, A. J. Mayhé Nunes, J. C. Santos, I. R. S. Cordeiro; individualCount: 2; sex: female; lifeStage: adult; occurrenceID: A3A8C96B-4D1D-5016-B719-287BCE1862F4; **Taxon:** genus: Pheidole; specificEpithet: tijucana; taxonRank: species; scientificNameAuthorship: Borgmeier, 1927; **Location:** continent: South America; country: Brazil; countryCode: BR; stateProvince: Rio de Janeiro; municipality: Rio de Janeiro; locality: Floresta da Tijuca, Parque Nacional da Tijuca, Bom Retiro (trilha para o pico da Tijuca); verbatimCoordinates: 22 56 48.2S 43 17 30.0W; verbatimLatitude: 22 56 48.2S; verbatimLongitude: 43 17 30.0W; verbatimCoordinateSystem: degrees minutes seconds; verbatimSRS: WGS84; **Identification:** identificationID: Pheidoletijucana; identifiedBy: M. M. Silva; **Event:** samplingProtocol: Pitfall; year: 2022; month: 1; day: 17-19; verbatimEventDate: Summer 2022; habitat: Atlantic Forest; **Record Level:** type: PhysicalObject; institutionCode: CEIOC; basisOfRecord: PreservedSpecimen
- **Type status:** Other material. **Occurrence:** recordNumber: WBC119; recordedBy: M. M. Silva, A. J. Mayhé Nunes, J. C. Santos, I. R. S. Cordeiro; individualCount: 1; sex: female; lifeStage: adult; occurrenceID: 1B3A5A8E-8A1B-568E-8DF6-4821C346B91F; **Taxon:** genus: Pheidole; specificEpithet: tijucana; taxonRank: species; scientificNameAuthorship: Borgmeier, 1927; **Location:** continent: South America; country: Brazil; countryCode: BR; stateProvince: Rio de Janeiro; municipality: Rio de Janeiro; locality: Floresta da Tijuca, Parque Nacional da Tijuca, trilha (caminho dos picos/ camminho da solidão); verbatimCoordinates: 22 57 28.5S 43 16 57.5W; verbatimLatitude: 22 57 28.5S; verbatimLongitude: 43 16 57.5W; verbatimCoordinateSystem: degrees minutes seconds; verbatimSRS: WGS84; **Identification:** identificationID: Pheidoletijucana; identifiedBy: M. M. Silva; **Event:** samplingProtocol: Winckler; year: 2022; month: 1; day: 19-21; verbatimEventDate: Summer 2022; habitat: Atlantic Forest; **Record Level:** type: PhysicalObject; institutionCode: CEIOC; basisOfRecord: PreservedSpecimen

##### Distribution

Endemic from eastern Brazil.

##### Notes

"Floresta da Tijuca" is the type locallity provided by Borgmeier (1927).

#### Rogeria lacertosa

Kempf, 1963

BD66D63D-6BC9-5DC7-843F-FBB2D47C93E7

##### Materials

- **Type status:** Other material. **Occurrence:** recordNumber: WAS106; recordedBy: M. M. Silva, A. J. Mayhé Nunes, J. C. Santos, I. R. S. Cordeiro; individualCount: 2; sex: female; lifeStage: adult; occurrenceID: 8DC9FE27-08EE-51AB-B8D7-7835D7CAB285; **Taxon:** genus: Rogeria; specificEpithet: lacertosa; taxonRank: species; scientificNameAuthorship: Kempf, 1963; **Location:** continent: South America; country: Brazil; countryCode: BR; stateProvince: Rio de Janeiro; municipality: Rio de Janeiro; locality: Floresta da Tijuca, Parque Nacional da Tijuca, Bom Retiro (trilha para o pico da Tijuca); verbatimCoordinates: 22 56 49.3S 43 17 29.1W; verbatimLatitude: 22 56 49.3S; verbatimLongitude: 43 17 29.1W; verbatimCoordinateSystem: degrees minutes seconds; verbatimSRS: WGS84; **Identification:** identificationID: Rogerialacertosa; identifiedBy: M. M. Silva; **Event:** samplingProtocol: Winckler; year: 2022; month: 1; day: 19-21; verbatimEventDate: Summer 2022; habitat: Atlantic Forest; **Record Level:** type: PhysicalObject; institutionCode: CEIOC; basisOfRecord: PreservedSpecimen

##### Distribution

Endemic from eastern Brazil.

##### Notes

This is the first record for State of Rio de Janeiro, contributing to fill a gap between Bahia to the north and Rio Grande do Sul to the south.

#### Sericomyrmex bondari

Weber, 1938

171D34FF-D72B-5A32-B54D-34A03EB927DE

##### Materials

- **Type status:** Other material. **Occurrence:** recordNumber: PBC108; recordedBy: M. M. Silva, A. J. Mayhé Nunes, J. C. Santos, I. R. S. Cordeiro; individualCount: 3; sex: female; lifeStage: adult; occurrenceID: C2803CAA-3EE9-56C1-B171-F15C22F5CE81; **Taxon:** genus: Sericomyrmex; specificEpithet: bondari; taxonRank: species; scientificNameAuthorship: Weber, 1938; **Location:** continent: South America; country: Brazil; countryCode: BR; stateProvince: Rio de Janeiro; municipality: Rio de Janeiro; locality: Floresta da Tijuca, Parque Nacional da Tijuca, trilha (caminho dos picos/ camminho da solidão); verbatimCoordinates: 22 57 23.7S 43 16 59.4W; verbatimLatitude: 22 57 23.7S; verbatimLongitude: 43 16 59.4W; verbatimCoordinateSystem: degrees minutes seconds; verbatimSRS: WGS84; **Identification:** identificationID: Sericomyrmexbondari; identifiedBy: M. M. Silva; **Event:** samplingProtocol: Pitfall; year: 2022; month: 1; day: 17-21; verbatimEventDate: Summer 2022; habitat: Atlantic Forest; **Record Level:** type: PhysicalObject; institutionCode: CEIOC; basisOfRecord: PreservedSpecimen

##### Distribution

Bolivia, Brazil, Colombia, Ecuador, French Guiana, Guyana, Peru, Suriname, Venezuela.

##### Notes

This is the first record for PNT. Klingenberg and Brandão (2005) recorded this species from the State of Rio de Janeiro without details.

#### Sericomyrmex saussurei

Emery, 1894

FDE23BE0-FC30-503A-8E0C-1C746492AD39

##### Materials

- **Type status:** Other material. **Occurrence:** recordNumber: WAS118; recordedBy: M. M. Silva, A. J. Mayhé Nunes, J. C. Santos, I. R. S. Cordeiro; individualCount: 1; sex: female; lifeStage: adult; occurrenceID: 3DC14678-E720-50FD-B55B-1EEE65A8B7AA; **Taxon:** genus: Sericomyrmex; specificEpithet: saussurei; taxonRank: species; scientificNameAuthorship: Emery, 1894; **Location:** continent: South America; country: Brazil; countryCode: BR; stateProvince: Rio de Janeiro; municipality: Rio de Janeiro; locality: Floresta da Tijuca, Parque Nacional da Tijuca, Bom Retiro (trilha para o pico da Tijuca); verbatimCoordinates: 22 56 45.6S 43 17 32.5W; verbatimLatitude: 22 56 45.6S; verbatimLongitude: 43 17 32.5W; verbatimCoordinateSystem: degrees minutes seconds; verbatimSRS: WGS84; **Identification:** identificationID: Sericomyrmexsaussurei; identifiedBy: M. M. Silva; **Event:** samplingProtocol: Winckler; year: 2021; month: 7; day: 19-21; verbatimEventDate: Winter 2021; habitat: Atlantic Forest; **Record Level:** type: PhysicalObject; institutionCode: CEIOC; basisOfRecord: PreservedSpecimen
- **Type status:** Other material. **Occurrence:** recordNumber: WBS120; recordedBy: M. M. Silva, A. J. Mayhé Nunes, J. C. Santos, I. R. S. Cordeiro; individualCount: 15; sex: female; lifeStage: adult; occurrenceID: 6AD9B191-70BD-5D68-942C-0337128E2E1F; **Taxon:** genus: Sericomyrmex; specificEpithet: saussurei; taxonRank: species; scientificNameAuthorship: Emery, 1894; **Location:** continent: South America; country: Brazil; countryCode: BR; stateProvince: Rio de Janeiro; municipality: Rio de Janeiro; locality: Floresta da Tijuca, Parque Nacional da Tijuca, trilha (caminho dos picos/ camminho da solidão); verbatimCoordinates: 22 57 30.0S 43 16 58.3W; verbatimLatitude: 22 57 30.0S; verbatimLongitude: 43 16 58.3W; verbatimCoordinateSystem: degrees minutes seconds; verbatimSRS: WGS84; **Identification:** identificationID: Sericomyrmexsaussurei; identifiedBy: M. M. Silva; **Event:** samplingProtocol: Winckler; year: 2021; month: 7; day: 21-23; verbatimEventDate: Winter 2021; habitat: Atlantic Forest; **Record Level:** type: PhysicalObject; institutionCode: CEIOC; basisOfRecord: PreservedSpecimen
- **Type status:** Other material. **Occurrence:** recordNumber: WCS108; recordedBy: M. M. Silva, A. J. Mayhé Nunes, J. C. Santos, I. R. S. Cordeiro; individualCount: 6; sex: female; lifeStage: adult; occurrenceID: 6126D637-A6BC-5E06-A338-C7B3753008E9; **Taxon:** genus: Sericomyrmex; specificEpithet: saussurei; taxonRank: species; scientificNameAuthorship: Emery, 1894; **Location:** continent: South America; country: Brazil; countryCode: BR; stateProvince: Rio de Janeiro; municipality: Rio de Janeiro; locality: Floresta da Tijuca, Parque Nacional da Tijuca, Trilha de Moutain bike (Açude da solidão).; verbatimCoordinates: 22 57 41.6S 43 17 27.2W; verbatimLatitude: 22 57 41.6S; verbatimLongitude: 43 17 27.2W; verbatimCoordinateSystem: degrees minutes seconds; verbatimSRS: WGS84; **Identification:** identificationID: Sericomyrmexsaussurei; identifiedBy: M. M. Silva; **Event:** samplingProtocol: Winckler; year: 2021; month: 7; day: 23-25; verbatimEventDate: Winter 2021; habitat: Atlantic Forest; **Record Level:** type: PhysicalObject; institutionCode: CEIOC; basisOfRecord: PreservedSpecimen

##### Distribution

Bolivia, Brazil, Colombia, Ecuador, Guyana, Lesser Antilles, Peru, Trinidad and Tobago, Venezuela. Records from Mexico need verification.

##### Notes

This is the first record for PNT. Ješovnik and Schultz (2017) recorded this species from the Reserva Biológica do Tinguá and the Parque Nacional da Serra dos Órgãos, which are protected areas in the State of Rio de Janeiro.

#### Strumigenys cosmostela

Kempf, 1975

8D500F53-F24D-529B-8C66-E424B234C560

##### Materials

- **Type status:** Other material. **Occurrence:** recordNumber: WCS108; recordedBy: M. M. Silva, A. J. Mayhé Nunes, J. C. Santos, I. R. S. Cordeiro; individualCount: 1; sex: female; lifeStage: adult; occurrenceID: 1F70D215-63C8-5700-8656-F86F42DA7857; **Taxon:** genus: Strumigenys; specificEpithet: cosmostela; taxonRank: species; scientificNameAuthorship: Kempf, 1975; **Location:** continent: South America; country: Brazil; countryCode: BR; stateProvince: Rio de Janeiro; municipality: Rio de Janeiro; locality: Floresta da Tijuca, Parque Nacional da Tijuca, Trilha de Moutain bike (Açude da solidão).; verbatimCoordinates: 22 57 41.6S 43 17 27.2W; verbatimLatitude: 22 57 41.6S; verbatimLongitude: 43 17 27.2W; verbatimCoordinateSystem: degrees minutes seconds; verbatimSRS: WGS84; **Identification:** identificationID: Strumigenyscosmostela; identifiedBy: M. M. Silva; **Event:** samplingProtocol: Winckler; year: 2021; month: 7; day: 23-25; verbatimEventDate: Winter 2021; habitat: Atlantic Forest; **Record Level:** type: PhysicalObject; institutionCode: CEIOC; basisOfRecord: PreservedSpecimen

##### Distribution

Brazil, Colombia, Costa Rica, Ecuador, French Guiana, Guatemala, Guyana, Honduras, Mexico, Nicaragua, Peru, Suriname, Venezuela.

##### Notes

This is the first record for PNT. This species was recorded by Silva and Brandão (2014) from the Parque Estadual do Desengano, another protected area in the State of Rio de Janeiro.

#### Strumigenys crassicornis

Mayr, 1887

DA5873DA-C600-5405-A88E-6E4B8CA9B618

##### Materials

- **Type status:** Other material. **Occurrence:** recordNumber: WAS116; recordedBy: M. M. Silva, A. J. Mayhé Nunes, J. C. Santos, I. R. S. Cordeiro; individualCount: 2; sex: female; lifeStage: adult; occurrenceID: 21042D28-B588-530E-854D-9EB340221E0B; **Taxon:** genus: Strumigenys; specificEpithet: crassicornis; taxonRank: species; scientificNameAuthorship: Mayr, 1887; **Location:** continent: South America; country: Brazil; countryCode: BR; stateProvince: Rio de Janeiro; municipality: Rio de Janeiro; locality: Floresta da Tijuca, Parque Nacional da Tijuca, Bom Retiro (trilha para o pico da Tijuca); verbatimCoordinates: 22 56 46.8S 43 17 33.6W; verbatimLatitude: 22 56 46.8S; verbatimLongitude: 43 17 33.6W; verbatimCoordinateSystem: degrees minutes seconds; verbatimSRS: WGS84; **Identification:** identificationID: Strumigenyscrassicornis; identifiedBy: M. M. Silva; **Event:** samplingProtocol: Winckler; year: 2021; month: 7; day: 19-21; verbatimEventDate: Winter 2021; habitat: Atlantic Forest; **Record Level:** type: PhysicalObject; institutionCode: CEIOC; basisOfRecord: PreservedSpecimen

##### Distribution

Argentina, Brazil, Colombia, French Guiana, Guyana, Lesser Antilles, Paraguay, Suriname, Trinidad and Tobago, Venezuela.

##### Notes

Previously recorded from "Floresta da Tijuca" by Silva and Brandão (2014) without details.

#### Strumigenys denticulata

Mayr, 1887

893644D4-4257-55A7-A87D-C74568816686

##### Materials

- **Type status:** Other material. **Occurrence:** recordNumber: WAS103; recordedBy: M. M. Silva, A. J. Mayhé Nunes, J. C. Santos, I. R. S. Cordeiro; individualCount: 7; sex: female; lifeStage: adult; occurrenceID: 6B57A8F8-8D68-5F59-A686-B75A2AEB0BAE; **Taxon:** genus: Strumigenys; specificEpithet: denticulata; taxonRank: species; scientificNameAuthorship: Mayr, 1887; **Location:** continent: South America; country: Brazil; countryCode: BR; stateProvince: Rio de Janeiro; municipality: Rio de Janeiro; locality: Floresta da Tijuca, Parque Nacional da Tijuca, Bom Retiro (trilha para o pico da Tijuca); verbatimCoordinates: 22 56 47.8S 43 17 30.4W; verbatimLatitude: 22 56 47.8S; verbatimLongitude: 43 17 30.4W; verbatimCoordinateSystem: degrees minutes seconds; verbatimSRS: WGS84; **Identification:** identificationID: Strumigenysdenticulata; identifiedBy: M. M. Silva; **Event:** samplingProtocol: Winckler; year: 2021; month: 7; day: 19-21; verbatimEventDate: Winter 2021; habitat: Atlantic Forest; **Record Level:** type: PhysicalObject; institutionCode: CEIOC; basisOfRecord: PreservedSpecimen
- **Type status:** Other material. **Occurrence:** recordNumber: WBS102; recordedBy: M. M. Silva, A. J. Mayhé Nunes, J. C. Santos, I. R. S. Cordeiro; individualCount: 24; sex: female; lifeStage: adult; occurrenceID: 67AD10AD-BF5F-5352-81EC-7721A5FD19BB; **Taxon:** genus: Strumigenys; specificEpithet: denticulata; taxonRank: species; scientificNameAuthorship: Mayr, 1887; **Location:** continent: South America; country: Brazil; countryCode: BR; stateProvince: Rio de Janeiro; municipality: Rio de Janeiro; locality: Floresta da Tijuca, Parque Nacional da Tijuca, trilha (caminho dos picos/ camminho da solidão); verbatimCoordinates: 22 57 24.0S 43 16 59.9W; verbatimLatitude: 22 57 24.0S; verbatimLongitude: 43 16 59.9W; verbatimCoordinateSystem: degrees minutes seconds; verbatimSRS: WGS84; **Identification:** identificationID: Strumigenysdenticulata; identifiedBy: M. M. Silva; **Event:** samplingProtocol: Winckler; year: 2021; month: 7; day: 21-23; verbatimEventDate: Winter 2021; habitat: Atlantic Forest; **Record Level:** type: PhysicalObject; institutionCode: CEIOC; basisOfRecord: PreservedSpecimen
- **Type status:** Other material. **Occurrence:** recordNumber: WCS102; recordedBy: M. M. Silva, A. J. Mayhé Nunes, J. C. Santos, I. R. S. Cordeiro; individualCount: 14; sex: female; lifeStage: adult; occurrenceID: 7A27B2FD-EAB2-52F1-BBBF-CB00B6193411; **Taxon:** genus: Strumigenys; specificEpithet: denticulata; taxonRank: species; scientificNameAuthorship: Mayr, 1887; **Location:** continent: South America; country: Brazil; countryCode: BR; stateProvince: Rio de Janeiro; municipality: Rio de Janeiro; locality: Floresta da Tijuca, Parque Nacional da Tijuca, Trilha de Moutain bike (Açude da solidão).; verbatimCoordinates: 22 57 42.0S 43 17 23.9W; verbatimLatitude: 22 57 42.0S; verbatimLongitude: 43 17 23.9W; verbatimCoordinateSystem: degrees minutes seconds; verbatimSRS: WGS84; **Identification:** identificationID: Strumigenysdenticulata; identifiedBy: M. M. Silva; **Event:** samplingProtocol: Winckler; year: 2021; month: 7; day: 23-25; verbatimEventDate: Winter 2021; habitat: Atlantic Forest; **Record Level:** type: PhysicalObject; institutionCode: CEIOC; basisOfRecord: PreservedSpecimen

##### Distribution

Argentina, Bolivia, Brazil, Colombia, Ecuador, French Guiana, Guyana, Panama, Paraguay, Peru, Suriname, Trinidad and Tobago, Venezuela

##### Notes

Previously recorded from "Floresta da Tijuca" by Silva and Brandão (2014) without details.

#### Strumigenys elongata

Roger, 1863

F42893A9-9591-5084-8D41-487709E2FC32

##### Materials

- **Type status:** Other material. **Occurrence:** recordNumber: WBS117; recordedBy: M. M. Silva, A. J. Mayhé Nunes, J. C. Santos, I. R. S. Cordeiro; individualCount: 1; sex: female; lifeStage: adult; occurrenceID: DF96EB89-E9A9-5C22-A01D-F73AD67320AD; **Taxon:** genus: Strumigenys; specificEpithet: elongata; taxonRank: species; scientificNameAuthorship: Roger, 1863; **Location:** continent: South America; country: Brazil; countryCode: BR; stateProvince: Rio de Janeiro; municipality: Rio de Janeiro; locality: Floresta da Tijuca, Parque Nacional da Tijuca, trilha (caminho dos picos/ camminho da solidão); verbatimCoordinates: 22 57 30.6S 43 16 58.1W; verbatimLatitude: 22 57 30.6S; verbatimLongitude: 43 16 58.1W; verbatimCoordinateSystem: degrees minutes seconds; verbatimSRS: WGS84; **Identification:** identificationID: Strumigenyselongata; identifiedBy: M. M. Silva; **Event:** samplingProtocol: Winckler; year: 2021; month: 7; day: 21-23; verbatimEventDate: Winter 2021; habitat: Atlantic Forest; **Record Level:** type: PhysicalObject; institutionCode: CEIOC; basisOfRecord: PreservedSpecimen
- **Type status:** Other material. **Occurrence:** recordNumber: WCC112; recordedBy: M. M. Silva, A. J. Mayhé Nunes, J. C. Santos, I. R. S. Cordeiro; individualCount: 2; sex: female; lifeStage: adult; occurrenceID: FDC26040-6211-5D3D-A6C0-D35D61005B0A; **Taxon:** genus: Strumigenys; specificEpithet: elongata; taxonRank: species; scientificNameAuthorship: Roger, 1863; **Location:** continent: South America; country: Brazil; countryCode: BR; stateProvince: Rio de Janeiro; municipality: Rio de Janeiro; locality: Floresta da Tijuca, Parque Nacional da Tijuca, Trilha de Moutain bike (Açude da solidão).; verbatimCoordinates: 22 57 42.4S 43 17 26.9W; verbatimLatitude: 22 57 42.4S; verbatimLongitude: 43 17 26.9W; verbatimCoordinateSystem: degrees minutes seconds; verbatimSRS: WGS84; **Identification:** identificationID: Strumigenyselongata; identifiedBy: M. M. Silva; **Event:** samplingProtocol: Winckler; year: 2022; month: 1; day: 21-23; verbatimEventDate: Summer 2022; habitat: Atlantic Forest; **Record Level:** type: PhysicalObject; institutionCode: CEIOC; basisOfRecord: PreservedSpecimen

##### Distribution

Argentina, Belize, Bolivia, Brazil, Colombia, Costa Rica, Ecuador, El Salvador, French Guiana, Guatemala, Guyana, Honduras, Lesser Antilles, Mexico, Nicaragua, Panama, Paraguay, Peru, San Andrés, Providencia and Santa Catalina, Suriname, Trinidad and Tobago, Venezuela.

##### Notes

Previously recorded from "Floresta da Tijuca" by Silva and Brandão (2014) without details.

#### Strumigenys hindenburgi

Forel, 1915

4D9037AF-77A0-579B-AB6C-E48AF28C5710

##### Materials

- **Type status:** Other material. **Occurrence:** recordNumber: WAS108; recordedBy: M. M. Silva, A. J. Mayhé Nunes, J. C. Santos, I. R. S. Cordeiro; individualCount: 2; sex: female; lifeStage: adult; occurrenceID: 49DCA430-4092-5731-BE24-D1DC0DCAAE99; **Taxon:** genus: Strumigenys; specificEpithet: hindenburgi; taxonRank: species; scientificNameAuthorship: Forel, 1915; **Location:** continent: South America; country: Brazil; countryCode: BR; stateProvince: Rio de Janeiro; municipality: Rio de Janeiro; locality: Floresta da Tijuca, Parque Nacional da Tijuca, Bom Retiro (trilha para o pico da Tijuca); verbatimCoordinates: 22 56 47.9S 43 17 31.3W; verbatimLatitude: 22 56 47.9S; verbatimLongitude: 43 17 31.3W; verbatimCoordinateSystem: degrees minutes seconds; verbatimSRS: WGS84; **Identification:** identificationID: Strumigenyshindenburgi; identifiedBy: M. M. Silva; **Event:** samplingProtocol: Winckler; year: 2021; month: 7; day: 19-21; verbatimEventDate: Winter 2021; habitat: Atlantic Forest; **Record Level:** type: PhysicalObject; institutionCode: CEIOC; basisOfRecord: PreservedSpecimen
- **Type status:** Other material. **Occurrence:** recordNumber: WBS107; recordedBy: M. M. Silva, A. J. Mayhé Nunes, J. C. Santos, I. R. S. Cordeiro; individualCount: 1; sex: female; lifeStage: adult; occurrenceID: 09614EF5-50C2-5527-834B-B4E2A161078E; **Taxon:** genus: Strumigenys; specificEpithet: hindenburgi; taxonRank: species; scientificNameAuthorship: Forel, 1915; **Location:** continent: South America; country: Brazil; countryCode: BR; stateProvince: Rio de Janeiro; municipality: Rio de Janeiro; locality: Floresta da Tijuca, Parque Nacional da Tijuca, trilha (caminho dos picos/ camminho da solidão); verbatimCoordinates: 22 57 24.4S 43 16 56.7W; verbatimLatitude: 22 57 24.4S; verbatimLongitude: 43 16 56.7W; verbatimCoordinateSystem: degrees minutes seconds; verbatimSRS: WGS84; **Identification:** identificationID: Strumigenyshindenburgi; identifiedBy: M. M. Silva; **Event:** samplingProtocol: Winckler; year: 2021; month: 7; day: 21-23; verbatimEventDate: Winter 2021; habitat: Atlantic Forest; **Record Level:** type: PhysicalObject; institutionCode: CEIOC; basisOfRecord: PreservedSpecimen
- **Type status:** Other material. **Occurrence:** recordNumber: WCS104; recordedBy: M. M. Silva, A. J. Mayhé Nunes, J. C. Santos, I. R. S. Cordeiro; individualCount: 1; sex: female; lifeStage: adult; occurrenceID: 66FBE3E5-A777-508C-B11B-1BC5C079C98D; **Taxon:** genus: Strumigenys; specificEpithet: hindenburgi; taxonRank: species; scientificNameAuthorship: Forel, 1915; **Location:** continent: South America; country: Brazil; countryCode: BR; stateProvince: Rio de Janeiro; municipality: Rio de Janeiro; locality: Floresta da Tijuca, Parque Nacional da Tijuca, Trilha de Moutain bike (Açude da solidão).; verbatimCoordinates: 22 57 41.8S 43 17 24.8W; verbatimLatitude: 22 57 41.8S; verbatimLongitude: 43 17 24.8W; verbatimCoordinateSystem: degrees minutes seconds; verbatimSRS: WGS84; **Identification:** identificationID: Strumigenyshindenburgi; identifiedBy: M. M. Silva; **Event:** samplingProtocol: Winckler; year: 2021; month: 7; day: 23-25; verbatimEventDate: Winter 2021; habitat: Atlantic Forest; **Record Level:** type: PhysicalObject; institutionCode: CEIOC; basisOfRecord: PreservedSpecimen

##### Distribution

Argentina, Brazil, Colombia, Paraguay.

##### Notes

This is the first record for PNT. This species was recorded by Bolton (2000) from the State of Rio de Janeiro without details.

#### Strumigenys subedentata

Mayr, 1887

E448E2F2-464B-5EE6-933E-C667D5091929

##### Materials

- **Type status:** Other material. **Occurrence:** recordNumber: WCS106; recordedBy: M. M. Silva, A. J. Mayhé Nunes, J. C. Santos, I. R. S. Cordeiro; individualCount: 1; sex: female; lifeStage: adult; occurrenceID: 2E04D618-1F19-5CEE-AD20-3CC0BD4696E5; **Taxon:** genus: Strumigenys; specificEpithet: subedentata; taxonRank: species; scientificNameAuthorship: Mayr, 1887; **Location:** continent: South America; country: Brazil; countryCode: BR; stateProvince: Rio de Janeiro; municipality: Rio de Janeiro; locality: Floresta da Tijuca, Parque Nacional da Tijuca, Trilha de Moutain bike (Açude da solidão).; verbatimCoordinates: 22 57 41.6S 43 17 25.6W; verbatimLatitude: 22 57 41.6S; verbatimLongitude: 43 17 25.6W; verbatimCoordinateSystem: degrees minutes seconds; verbatimSRS: WGS84; **Identification:** identificationID: Strumigenyssubedentata; identifiedBy: M. M. Silva; **Event:** samplingProtocol: Winckler; year: 2021; month: 7; day: 23-25; verbatimEventDate: Winter 2021; habitat: Atlantic Forest; **Record Level:** type: PhysicalObject; institutionCode: CEIOC; basisOfRecord: PreservedSpecimen

##### Distribution

Argentina, Belize, Brazil, Colombia, Costa Rica, Ecuador, French Guiana, Guatemala, Guyana, Honduras, Lesser Antilles, Mexico, Nicaragua, Panama, Paraguay, Peru, Suriname, Trinidad and Tobago, Venezuela.

##### Notes

Previously recorded from "Floresta da Tijuca" by Silva and Brandão (2014) without details.

#### Paratrachymyrmex cornetzi

(Forel, 1912)

2A1B1617-9783-5277-B231-60FBE14ACC0D

##### Materials

- **Type status:** Other material. **Occurrence:** recordNumber: WBS112; recordedBy: M. M. Silva, A. J. Mayhé Nunes, J. C. Santos, I. R. S. Cordeiro; individualCount: 6; sex: female; lifeStage: adult; occurrenceID: FAF514D9-DAA5-5B37-814B-EBC9BA4FA833; **Taxon:** genus: Paratrachymyrmex; specificEpithet: cornetzi; taxonRank: species; scientificNameAuthorship: (Forel, 1912); **Location:** continent: South America; country: Brazil; countryCode: BR; stateProvince: Rio de Janeiro; municipality: Rio de Janeiro; locality: Floresta da Tijuca, Parque Nacional da Tijuca, trilha (caminho dos picos/ camminho da solidão); verbatimCoordinates: 22 57 37.0S 43 16 55.4W; verbatimLatitude: 22 57 37.0S; verbatimLongitude: 43 16 55.4W; verbatimCoordinateSystem: degrees minutes seconds; verbatimSRS: WGS84; **Identification:** identificationID: Paratrachymyrmexcornetzi; identifiedBy: A. J. Mayhé-Nunes; **Event:** samplingProtocol: Winckler; year: 2021; month: 7; day: 21-23; verbatimEventDate: Winter 2021; habitat: Atlantic Forest; **Record Level:** type: PhysicalObject; institutionCode: CEIOC; basisOfRecord: PreservedSpecimen

##### Distribution

Brazil, Colombia, Costa Rica, Ecuador, French Guiana, Guatemala, Guyana, Honduras, Lesser Antilles, Mexico, Nicaragua, Panama, Peru, Suriname, Trinidad and Tobago, Venezuela.

##### Notes

This is the first record for Floresta da Tijuca sector. Previously recorded by Santos et al. (2019) from the Rio de Janeiro Botanical Garden, within the Serra da Carioca sector.

#### Wasmannia affinis

Santschi, 1929

1025499E-E521-56C8-A4B9-CFA30AF92C87

##### Materials

- **Type status:** Other material. **Occurrence:** recordNumber: WAC113; recordedBy: M. M. Silva, A. J. Mayhé Nunes, J. C. Santos, I. R. S. Cordeiro; individualCount: 5; sex: female; lifeStage: adult; occurrenceID: 6DC94746-716F-5348-A943-39C1E8A590C0; **Taxon:** genus: Wasmannia; specificEpithet: affinis; taxonRank: species; scientificNameAuthorship: Santschi, 1929; **Location:** continent: South America; country: Brazil; countryCode: BR; stateProvince: Rio de Janeiro; municipality: Rio de Janeiro; locality: Floresta da Tijuca, Parque Nacional da Tijuca, Bom Retiro (trilha para o pico da Tijuca); verbatimCoordinates: 22 56 44.6S 43 17 32.1W; verbatimLatitude: 22 56 44.6S; verbatimLongitude: 43 17 32.1W; verbatimCoordinateSystem: degrees minutes seconds; verbatimSRS: WGS84; **Identification:** identificationID: Wasmanniaaffinis; identifiedBy: M. M. Silva; **Event:** samplingProtocol: Winckler; year: 2022; month: 1; day: 17-19; verbatimEventDate: Summer 2022; habitat: Atlantic Forest; **Record Level:** type: PhysicalObject; institutionCode: CEIOC; basisOfRecord: PreservedSpecimen
- **Type status:** Other material. **Occurrence:** recordNumber: WBS109; recordedBy: M. M. Silva, A. J. Mayhé Nunes, J. C. Santos, I. R. S. Cordeiro; individualCount: 14; sex: female; lifeStage: adult; occurrenceID: 03469F9D-D075-5D24-B823-C1868FADD45E; **Taxon:** genus: Wasmannia; specificEpithet: affinis; taxonRank: species; scientificNameAuthorship: Santschi, 1929; **Location:** continent: South America; country: Brazil; countryCode: BR; stateProvince: Rio de Janeiro; municipality: Rio de Janeiro; locality: Floresta da Tijuca, Parque Nacional da Tijuca, trilha (caminho dos picos/ camminho da solidão); verbatimCoordinates: 22 57 25.3S 43 16 56.2W; verbatimLatitude: 22 57 25.3S; verbatimLongitude: 43 16 56.2W; verbatimCoordinateSystem: degrees minutes seconds; verbatimSRS: WGS84; **Identification:** identificationID: Wasmanniaaffinis; identifiedBy: M. M. Silva; **Event:** samplingProtocol: Winckler; year: 2021; month: 7; day: 21-23; verbatimEventDate: Winter 2021; habitat: Atlantic Forest; **Record Level:** type: PhysicalObject; institutionCode: CEIOC; basisOfRecord: PreservedSpecimen
- **Type status:** Other material. **Occurrence:** recordNumber: WCS101; recordedBy: M. M. Silva, A. J. Mayhé Nunes, J. C. Santos, I. R. S. Cordeiro; individualCount: 2; sex: female; lifeStage: adult; occurrenceID: B6BA0D36-0F76-5117-9F8E-F14B88BC70EF; **Taxon:** genus: Wasmannia; specificEpithet: affinis; taxonRank: species; scientificNameAuthorship: Santschi, 1929; **Location:** continent: South America; country: Brazil; countryCode: BR; stateProvince: Rio de Janeiro; municipality: Rio de Janeiro; locality: Floresta da Tijuca, Parque Nacional da Tijuca, Bom Retiro (trilha para o pico da Tijuca); verbatimCoordinates: 22 57 42.4S 43 17 23.8W; verbatimLatitude: 22 57 42.4S; verbatimLongitude: 43 17 23.8W; verbatimCoordinateSystem: degrees minutes seconds; verbatimSRS: WGS84; **Identification:** identificationID: Wasmanniaaffinis; identifiedBy: M. M. Silva; **Event:** samplingProtocol: Winckler; year: 2021; month: 7; day: 23-25; verbatimEventDate: Winter 2021; habitat: Atlantic Forest; **Record Level:** type: PhysicalObject; institutionCode: CEIOC; basisOfRecord: PreservedSpecimen

##### Distribution

Endemic from eastern Brazil.

##### Notes

This is the first record for PNT. Previously recorded by Lasmar et al. (2020) from another protected area in the State of Rio de Janeiro.

#### Wasmannia auropunctata

(Roger, 1863)

FDACE415-60AA-57C1-9B37-417453C70BF8

##### Materials

- **Type status:** Other material. **Occurrence:** recordNumber: PCS115; recordedBy: M. M. Silva, A. J. Mayhé Nunes, J. C. Santos, I. R. S. Cordeiro; individualCount: 15; sex: female; lifeStage: adult; occurrenceID: C1D2B3F1-D9E7-5967-94FF-BBDF8822C86C; **Taxon:** genus: Wasmannia; specificEpithet: auropunctata; taxonRank: species; scientificNameAuthorship: (Roger, 1863); **Location:** continent: South America; country: Brazil; countryCode: BR; stateProvince: Rio de Janeiro; municipality: Rio de Janeiro; locality: Floresta da Tijuca, Parque Nacional da Tijuca, Trilha de Moutain bike (Açude da solidão).; verbatimCoordinates: 22 57 41.9S 43 17 23.3W; verbatimLatitude: 22 57 41.9S; verbatimLongitude: 43 17 23.3W; verbatimCoordinateSystem: degrees minutes seconds; verbatimSRS: WGS84; **Identification:** identificationID: Wasmanniaauropunctata; identifiedBy: M. M. Silva; **Event:** samplingProtocol: Pitfall; year: 2021; month: 7; day: 23-25; verbatimEventDate: Winter 2021; habitat: Atlantic Forest; **Record Level:** type: PhysicalObject; institutionCode: CEIOC; basisOfRecord: PreservedSpecimen

##### Distribution

Argentina, Belize, Bolivia, Brazil, Colombia, Costa Rica, Ecuador, El Salvador, French Guiana, Guatemala, Guyana, Honduras, Nicaragua, Panama, Paraguay, Peru, Suriname, Uruguay, Venezuela. Introduced to Canada, midwest United States, Germany, United Kingdom, the Netherlands and New Zealand. Exotic in portions of the Afrotropical, Australian, Nearctic, Neotropical, Oceanian, Oriental, Palearctic, Panamanian, Saharo-Arabian and Sino-Japanese Regions. Records from the State of Arizona (United States), South Africa, Keeling Islands, Indonesia, New Guinea and Cook Islands need verification. Records from continental Italy and Tuvalu are dubious.

##### Notes

This is the first record for Floresta da Tijuca sector. Previously recorded by Vargas et al. (2013) and Santos et al. (2019) from the Rio de Janeiro Botanical Garden and the Reserva Florestal da Vista Chinesa, both in the Serra da Carioca sector.

#### Wasmannia lutzi

Forel, 1908

050F9580-0BD7-57EA-9A10-CFAEB676E1D5

##### Materials

- **Type status:** Other material. **Occurrence:** recordNumber: WAS112; recordedBy: M. M. Silva, A. J. Mayhé Nunes, J. C. Santos, I. R. S. Cordeiro; individualCount: 4; sex: female; lifeStage: adult; occurrenceID: EB564B31-1F53-51EC-B300-84961122D0C0; **Taxon:** genus: Wasmannia; specificEpithet: lutzi; taxonRank: species; scientificNameAuthorship: Forel, 1908; **Location:** continent: South America; country: Brazil; countryCode: BR; stateProvince: Rio de Janeiro; municipality: Rio de Janeiro; locality: Floresta da Tijuca, Parque Nacional da Tijuca, Bom Retiro (trilha para o pico da Tijuca); verbatimCoordinates: 22 56 48.2S 43 17 35.4W; verbatimLatitude: 22 56 48.2S; verbatimLongitude: 43 17 35.4W; verbatimCoordinateSystem: degrees minutes seconds; verbatimSRS: WGS84; **Identification:** identificationID: Wasmannialutzi; identifiedBy: M. M. Silva; **Event:** samplingProtocol: Winckler; year: 2021; month: 7; day: 19-21; verbatimEventDate: Winter 2021; habitat: Atlantic Forest; **Record Level:** type: PhysicalObject; institutionCode: CEIOC; basisOfRecord: PreservedSpecimen
- **Type status:** Other material. **Occurrence:** recordNumber: WBS105; recordedBy: M. M. Silva, A. J. Mayhé Nunes, J. C. Santos, I. R. S. Cordeiro; individualCount: 7; sex: female; lifeStage: adult; occurrenceID: 58CA40CB-034D-5BE8-B5C9-5FFDEE099605; **Taxon:** genus: Wasmannia; specificEpithet: lutzi; taxonRank: species; scientificNameAuthorship: Forel, 1908; **Location:** continent: South America; country: Brazil; countryCode: BR; stateProvince: Rio de Janeiro; municipality: Rio de Janeiro; locality: Floresta da Tijuca, Parque Nacional da Tijuca, trilha (caminho dos picos/ camminho da solidão); verbatimCoordinates: 22 57 23.8S 43 16 57.7W; verbatimLatitude: 22 57 23.8S; verbatimLongitude: 43 16 57.7W; verbatimCoordinateSystem: degrees minutes seconds; verbatimSRS: WGS84; **Identification:** identificationID: Wasmannialutzi; identifiedBy: M. M. Silva; **Event:** samplingProtocol: Winckler; year: 2021; month: 7; day: 21-23; verbatimEventDate: Winter 2021; habitat: Atlantic Forest; **Record Level:** type: PhysicalObject; institutionCode: CEIOC; basisOfRecord: PreservedSpecimen

##### Distribution

Brazil, Paraguay.

##### Notes

This is the first record for the PNT. Previously recorded by Orsolon-Souza et al. (2011) and Silva and Brandão (2014) from other protected areas in the state of Rio de Janeiro.

#### Pseudomyrmicinae

Smith, M.R., 1952

B1740482-54C7-521B-BB4A-ADBDC906680B

#### Pseudomyrmex schuppi

(Forel, 1901)

B6D0C7BE-79AA-5914-955A-C700072EE2A5

##### Materials

- **Type status:** Other material. **Occurrence:** recordNumber: PBC105; recordedBy: M. M. Silva, A. J. Mayhé Nunes, J. C. Santos, I. R. S. Cordeiro; individualCount: 1; sex: female; lifeStage: adult; occurrenceID: 4396E676-7171-5E49-A377-6B3B77123D04; **Taxon:** genus: Pseudomyrmex; specificEpithet: schuppi; taxonRank: species; scientificNameAuthorship: (Forel, 1901); **Location:** continent: South America; country: Brazil; countryCode: BR; stateProvince: Rio de Janeiro; municipality: Rio de Janeiro; locality: Floresta da Tijuca, Parque Nacional da Tijuca, Bom Retiro (trilha para o pico da Tijuca); verbatimCoordinates: 22 57 2.0S 43 16 59.5W; verbatimLatitude: 22 57 2.0S; verbatimLongitude: 43 16 59.5W; verbatimCoordinateSystem: degrees minutes seconds; verbatimSRS: WGS84; **Identification:** identificationID: Pseudomyrmexschuppi; identifiedBy: M. M. Silva; **Event:** samplingProtocol: Pitfall; year: 2021; month: 1; day: 17-20; verbatimEventDate: Summer 2021; habitat: Atlantic Forest; **Record Level:** type: PhysicalObject; institutionCode: CEIOC; basisOfRecord: PreservedSpecimen

##### Distribution

Argentina, Brazil, Paraguay, Uruguay.

##### Notes

This is the first record for Floresta da Tijuca sector. Ward (1989) and Santos et al. (2019) recorded this species from the Corcovado Mountain, in the Serra da Carioca sector.

#### Ponerinae

Lepeletier de Saint-Fargeau, 1835

09496349-D5F1-5CEC-B235-BFB3F167CF09

#### Anochetus mayri

Emery, 1884

97B3DD10-3172-5FA4-9E9D-3948497BCD77

##### Materials

- **Type status:** Other material. **Occurrence:** recordNumber: WAC120; recordedBy: M. M. Silva, A. J. Mayhé Nunes, J. C. Santos, I. R. S. Cordeiro; individualCount: 4; sex: female; lifeStage: adult; occurrenceID: 2FF51B11-A98D-53B8-A532-DB1F6DD08229; **Taxon:** genus: Anochetus; specificEpithet: mayri; taxonRank: species; scientificNameAuthorship: Emery, 1884; **Location:** continent: South America; country: Brazil; countryCode: BR; stateProvince: Rio de Janeiro; municipality: Rio de Janeiro; locality: Floresta da Tijuca, Parque Nacional da Tijuca, Bom Retiro (trilha para o pico da Tijuca); verbatimCoordinates: 22 56 43.3S 43 17 28.9W; verbatimLatitude: 22 56 43.3S; verbatimLongitude: 43 17 28.9W; verbatimCoordinateSystem: degrees minutes seconds; verbatimSRS: WGS84; **Identification:** identificationID: Anochetusmayri; identifiedBy: M. M. Silva; **Event:** samplingProtocol: Winckler; year: 2022; month: 1; day: 17-19; verbatimEventDate: Summer 2022; habitat: Atlantic Forest; **Record Level:** type: PhysicalObject; institutionCode: CEIOC; basisOfRecord: PreservedSpecimen
- **Type status:** Other material. **Occurrence:** recordNumber: WBS102; recordedBy: M. M. Silva, A. J. Mayhé Nunes, J. C. Santos, I. R. S. Cordeiro; individualCount: 9; sex: female; lifeStage: adult; occurrenceID: A1F78F9B-C980-54DD-8D3D-B0D67E8CBE54; **Taxon:** genus: Anochetus; specificEpithet: mayri; taxonRank: species; scientificNameAuthorship: Emery, 1884; **Location:** continent: South America; country: Brazil; countryCode: BR; stateProvince: Rio de Janeiro; municipality: Rio de Janeiro; locality: Floresta da Tijuca, Parque Nacional da Tijuca, trilha (caminho dos picos/ camminho da solidão); verbatimCoordinates: 22 57 24.0S 43 16 59.9W; verbatimLatitude: 22 57 24.0S; verbatimLongitude: 43 16 59.9W; verbatimCoordinateSystem: degrees minutes seconds; verbatimSRS: WGS84; **Identification:** identificationID: Anochetusmayri; identifiedBy: M. M. Silva; **Event:** samplingProtocol: Winckler; year: 2021; month: 7; day: 21-23; verbatimEventDate: Winter 2021; habitat: Atlantic Forest; **Record Level:** type: PhysicalObject; institutionCode: CEIOC; basisOfRecord: PreservedSpecimen

##### Distribution

Argentina, Bahamas, Belize, Bolivia, Brazil, Colombia, Costa Rica, Cuba, Ecuador, El Salvador, French Guiana, Guatemala, Guyana, Hispaniola, Honduras, Jamaica, Lesser Antilles, Mexico, Nicaragua, Panama, Paraguay, Peru, Puerto Rico, Suriname, Trinidad and Tobago, Uruguay, Venezuela. Exotic in southeast United States. Introduced to the United Kingdom and Denmark.

##### Notes

This is the first record for Floresta da Tijuca sector. Previously recorded by Vargas et al. (2013) and Santos et al. (2019) from the Serra da Carioca sector.

#### Hypoponera distinguenda

(Emery, 1890)

0D2C9F1C-5305-50E6-AD3B-A56FB47E1A1A

##### Materials

- **Type status:** Other material. **Occurrence:** recordNumber: WAS113; recordedBy: M. M. Silva, A. J. Mayhé Nunes, J. C. Santos, I. R. S. Cordeiro; individualCount: 1; sex: female; lifeStage: adult; occurrenceID: DBBF66B6-0158-5AEA-A8BC-324694D90CDA; **Taxon:** genus: Hypoponera; specificEpithet: distinguenda; taxonRank: species; scientificNameAuthorship: (Emery, 1890); **Location:** continent: South America; country: Brazil; countryCode: BR; stateProvince: Rio de Janeiro; municipality: Rio de Janeiro; locality: Floresta da Tijuca, Parque Nacional da Tijuca, Bom Retiro (trilha para o pico da Tijuca); verbatimCoordinates: 22 56 48.5S 43 17 35.3W; verbatimLatitude: 22 56 48.5S; verbatimLongitude: 43 17 35.3W; verbatimCoordinateSystem: degrees minutes seconds; verbatimSRS: WGS84; **Identification:** identificationID: Hypoponeradistinguenda; identifiedBy: M. M. Silva; **Event:** samplingProtocol: Winckler; year: 2021; month: 7; day: 19-21; verbatimEventDate: Winter 2021; habitat: Atlantic Forest; **Record Level:** type: PhysicalObject; institutionCode: CEIOC; basisOfRecord: PreservedSpecimen
- **Type status:** Other material. **Occurrence:** recordNumber: WCC110; recordedBy: M. M. Silva, A. J. Mayhé Nunes, J. C. Santos, I. R. S. Cordeiro; individualCount: 2; sex: female; lifeStage: adult; occurrenceID: 7B2AAFA4-A612-5314-978C-5059EAB88591; **Taxon:** genus: Hypoponera; specificEpithet: distinguenda; taxonRank: species; scientificNameAuthorship: (Emery, 1890); **Location:** continent: South America; country: Brazil; countryCode: BR; stateProvince: Rio de Janeiro; municipality: Rio de Janeiro; locality: Floresta da Tijuca, Parque Nacional da Tijuca, Trilha de Moutain bike (Açude da solidão).; verbatimCoordinates: 22 56 43.3S 43 17 28.9W; verbatimLatitude: 22 56 43.3S; verbatimLongitude: 43 17 28.9W; verbatimCoordinateSystem: degrees minutes seconds; verbatimSRS: WGS84; **Identification:** identificationID: Hypoponeradistinguenda; identifiedBy: M. M. Silva; **Event:** samplingProtocol: Winckler; year: 2022; month: 1; day: 21-23; verbatimEventDate: Summer 2022; habitat: Atlantic Forest; **Record Level:** type: PhysicalObject; institutionCode: CEIOC; basisOfRecord: PreservedSpecimen

##### Distribution

Argentina, Bolivia, Brazil, Colombia, Costa Rica, Ecuador, Guatemala, Honduras, Mexico, Panama, Paraguay, Peru, Trinidad and Tobago, Venezuela.

##### Notes

This is the first record for PNT. This species was previously recorded by Kempf (1972) from the State of Rio de Janeiro without details.

#### Hypoponera foreli

(Mayr, 1887)

F8573DBF-2FBF-522D-B12E-BEA0F1434669

##### Materials

- **Type status:** Other material. **Occurrence:** recordNumber: WAC115; recordedBy: M. M. Silva, A. J. Mayhé Nunes, J. C. Santos, I. R. S. Cordeiro; individualCount: 14; sex: female; lifeStage: adult; occurrenceID: 9BD524D6-8DEA-5416-826C-6EF65DE3BBF6; **Taxon:** genus: Hypoponera; specificEpithet: foreli; taxonRank: species; scientificNameAuthorship: (Mayr, 1887); **Location:** continent: South America; country: Brazil; countryCode: BR; stateProvince: Rio de Janeiro; municipality: Rio de Janeiro; locality: Floresta da Tijuca, Parque Nacional da Tijuca, Bom Retiro (trilha para o pico da Tijuca); verbatimCoordinates: 22 56 44.0S 43 17 31.2W; verbatimLatitude: 22 56 44.0S; verbatimLongitude: 43 17 31.2W; verbatimCoordinateSystem: degrees minutes seconds; verbatimSRS: WGS84; **Identification:** identificationID: Hypoponeraforeli; identifiedBy: M. M. Silva; **Event:** samplingProtocol: Winckler; year: 2022; month: 1; day: 17-19; verbatimEventDate: Summer 2022; habitat: Atlantic Forest; **Record Level:** type: PhysicalObject; institutionCode: CEIOC; basisOfRecord: PreservedSpecimen
- **Type status:** Other material. **Occurrence:** recordNumber: WBS111; recordedBy: M. M. Silva, A. J. Mayhé Nunes, J. C. Santos, I. R. S. Cordeiro; individualCount: 6; sex: female; lifeStage: adult; occurrenceID: B6145AE8-05ED-58E7-A3F4-82BB0FA42CBA; **Taxon:** genus: Hypoponera; specificEpithet: foreli; taxonRank: species; scientificNameAuthorship: (Mayr, 1887); **Location:** continent: South America; country: Brazil; countryCode: BR; stateProvince: Rio de Janeiro; municipality: Rio de Janeiro; locality: Floresta da Tijuca, Parque Nacional da Tijuca, trilha (caminho dos picos/ camminho da solidão); verbatimCoordinates: 22 57 26.8S 43 16 55.5W; verbatimLatitude: 22 57 26.8S; verbatimLongitude: 43 16 55.5W; verbatimCoordinateSystem: degrees minutes seconds; verbatimSRS: WGS84; **Identification:** identificationID: Hypoponeraforeli; identifiedBy: M. M. Silva; **Event:** samplingProtocol: Winckler; year: 2021; month: 7; day: 21-23; verbatimEventDate: Winter 2021; habitat: Atlantic Forest; **Record Level:** type: PhysicalObject; institutionCode: CEIOC; basisOfRecord: PreservedSpecimen
- **Type status:** Other material. **Occurrence:** recordNumber: WCS108; recordedBy: M. M. Silva, A. J. Mayhé Nunes, J. C. Santos, I. R. S. Cordeiro; individualCount: 9; sex: female; lifeStage: adult; occurrenceID: B31B3F4C-3407-5028-ACE3-15B3C6895434; **Taxon:** genus: Hypoponera; specificEpithet: foreli; taxonRank: species; scientificNameAuthorship: (Mayr, 1887); **Location:** continent: South America; country: Brazil; countryCode: BR; stateProvince: Rio de Janeiro; municipality: Rio de Janeiro; locality: Floresta da Tijuca, Parque Nacional da Tijuca, Trilha de Moutain bike (Açude da solidão).; verbatimCoordinates: 22 57 41.6S 43 17 27.2W; verbatimLatitude: 22 57 41.6S; verbatimLongitude: 43 17 27.2W; verbatimCoordinateSystem: degrees minutes seconds; verbatimSRS: WGS84; **Identification:** identificationID: Hypoponeraforeli; identifiedBy: M. M. Silva; **Event:** samplingProtocol: Winckler; year: 2021; month: 7; day: 23-25; verbatimEventDate: Winter 2021; habitat: Atlantic Forest; **Record Level:** type: PhysicalObject; institutionCode: CEIOC; basisOfRecord: PreservedSpecimen

##### Distribution

Argentina, Bolivia, Brazil, Costa Rica, Ecuador, Paraguay, Peru, Venezuela.

##### Notes

This is the first record for Floresta da Tijuca sector. Previously recorded by Santos et al. (2019) from the Serra da Carioca sector.

#### Hypoponera leveillei

(Emery, 1890)

DD030BF6-36A1-5D0E-A8C5-2EBF15B43687

##### Materials

- **Type status:** Other material. **Occurrence:** recordNumber: WAS112; recordedBy: M. M. Silva, A. J. Mayhé Nunes, J. C. Santos, I. R. S. Cordeiro; individualCount: 2; sex: female; lifeStage: adult; occurrenceID: C8FAAEE9-FF61-53DD-8B5E-DEE772BB5E26; **Taxon:** genus: Hypoponera; specificEpithet: leveillei; taxonRank: species; scientificNameAuthorship: (Emery, 1890); **Location:** continent: South America; country: Brazil; countryCode: BR; stateProvince: Rio de Janeiro; municipality: Rio de Janeiro; locality: Floresta da Tijuca, Parque Nacional da Tijuca, Bom Retiro (trilha para o pico da Tijuca); verbatimCoordinates: 22 56 48.2S 43 17 35.4W; verbatimLatitude: 22 56 48.2S; verbatimLongitude: 43 17 35.4W; verbatimCoordinateSystem: degrees minutes seconds; verbatimSRS: WGS84; **Identification:** identificationID: Hypoponeraleveillei; identifiedBy: M. M. Silva; **Event:** samplingProtocol: Winckler; year: 2021; month: 7; day: 19-21; verbatimEventDate: Winter 2021; habitat: Atlantic Forest; **Record Level:** type: PhysicalObject; institutionCode: CEIOC; basisOfRecord: PreservedSpecimen
- **Type status:** Other material. **Occurrence:** recordNumber: WCC108; recordedBy: M. M. Silva, A. J. Mayhé Nunes, J. C. Santos, I. R. S. Cordeiro; individualCount: 1; sex: female; lifeStage: adult; occurrenceID: 75D998F4-5A2F-5757-8D0E-E8734FDBABF8; **Taxon:** genus: Hypoponera; specificEpithet: leveillei; taxonRank: species; scientificNameAuthorship: (Emery, 1890); **Location:** continent: South America; country: Brazil; countryCode: BR; stateProvince: Rio de Janeiro; municipality: Rio de Janeiro; locality: Floresta da Tijuca, Parque Nacional da Tijuca, Trilha de Moutain bike (Açude da solidão).; verbatimCoordinates: 22 57 41.7S 43 17 24.7W; verbatimLatitude: 22 57 41.7S; verbatimLongitude: 43 17 24.7W; verbatimCoordinateSystem: degrees minutes seconds; verbatimSRS: WGS84; **Identification:** identificationID: Hypoponeraleveillei; identifiedBy: M. M. Silva; **Event:** samplingProtocol: Winckler; year: 2022; month: 1; day: 21-23; verbatimEventDate: Summer 2022; habitat: Atlantic Forest; **Record Level:** type: PhysicalObject; institutionCode: CEIOC; basisOfRecord: PreservedSpecimen

##### Distribution

Bolivia, Brazil, Panama, Venezuela.

##### Notes

This is the first record for PNT. Previously recorded by Mackay and Mackay (2010) from the State of Rio de Janeiro without details.

#### Hypoponera parva

(Forel, 1909)

BECC282F-B0A1-5AF8-BF6E-F6354B6F6378

##### Materials

- **Type status:** Other material. **Occurrence:** recordNumber: WAS106; recordedBy: M. M. Silva, A. J. Mayhé Nunes, J. C. Santos, I. R. S. Cordeiro; individualCount: 12; sex: female; lifeStage: adult; occurrenceID: F25DF1A7-52B3-55EF-9C99-98325BBCCCE7; **Taxon:** genus: Hypoponera; specificEpithet: parva; taxonRank: species; scientificNameAuthorship: (Forel, 1909); **Location:** continent: South America; country: Brazil; countryCode: BR; stateProvince: Rio de Janeiro; municipality: Rio de Janeiro; locality: Floresta da Tijuca, Parque Nacional da Tijuca, Bom Retiro (trilha para o pico da Tijuca); verbatimCoordinates: 22 56 49.3S 43 17 29.1W; verbatimLatitude: 22 56 49.3S; verbatimLongitude: 43 17 29.1W; verbatimCoordinateSystem: degrees minutes seconds; verbatimSRS: WGS84; **Identification:** identificationID: Hypoponeraparva; identifiedBy: M. M. Silva; **Event:** samplingProtocol: Winckler; year: 2021; month: 7; day: 19-21; verbatimEventDate: Winter 2021; habitat: Atlantic Forest; **Record Level:** type: PhysicalObject; institutionCode: CEIOC; basisOfRecord: PreservedSpecimen
- **Type status:** Other material. **Occurrence:** recordNumber: WBS105; recordedBy: M. M. Silva, A. J. Mayhé Nunes, J. C. Santos, I. R. S. Cordeiro; individualCount: 6; sex: female; lifeStage: adult; occurrenceID: 8735219A-9F11-574E-B761-04D3A29EBDD2; **Taxon:** genus: Hypoponera; specificEpithet: parva; taxonRank: species; scientificNameAuthorship: (Forel, 1909); **Location:** continent: South America; country: Brazil; countryCode: BR; stateProvince: Rio de Janeiro; municipality: Rio de Janeiro; locality: Floresta da Tijuca, Parque Nacional da Tijuca, trilha (caminho dos picos/ camminho da solidão); verbatimCoordinates: 22 57 23.8S 43 26 57.7W; verbatimLatitude: 22 57 23.8S; verbatimLongitude: 43 26 57.7W; verbatimCoordinateSystem: degrees minutes seconds; verbatimSRS: WGS84; **Identification:** identificationID: Hypoponeraparva; identifiedBy: M. M. Silva; **Event:** samplingProtocol: Winckler; year: 2021; month: 7; day: 21-23; verbatimEventDate: Winter 2021; habitat: Atlantic Forest; **Record Level:** type: PhysicalObject; institutionCode: CEIOC; basisOfRecord: PreservedSpecimen
- **Type status:** Other material. **Occurrence:** recordNumber: WCS104; recordedBy: M. M. Silva, A. J. Mayhé Nunes, J. C. Santos, I. R. S. Cordeiro; individualCount: 4; sex: female; lifeStage: adult; occurrenceID: 82406CC3-A89B-5B0A-B5DD-7BCCF0B47E35; **Taxon:** genus: Hypoponera; specificEpithet: parva; taxonRank: species; scientificNameAuthorship: (Forel, 1909); **Location:** continent: South America; country: Brazil; countryCode: BR; stateProvince: Rio de Janeiro; municipality: Rio de Janeiro; locality: Floresta da Tijuca, Parque Nacional da Tijuca, Trilha de Moutain bike (Açude da solidão).; verbatimCoordinates: 22 57 41.8S 43 17 24.8W; verbatimLatitude: 22 57 41.8S; verbatimLongitude: 43 17 24.8W; verbatimCoordinateSystem: degrees minutes seconds; verbatimSRS: WGS84; **Identification:** identificationID: Hypoponeraparva; identifiedBy: M. M. Silva; **Event:** samplingProtocol: Winckler; year: 2021; month: 7; day: 23-25; verbatimEventDate: Winter 2021; habitat: Atlantic Forest; **Record Level:** type: PhysicalObject; institutionCode: CEIOC; basisOfRecord: PreservedSpecimen

##### Distribution

Argentina, Belize, Bolivia, Brazil, Colombia, Costa Rica, Cuba, Ecuador, Guatemala, Honduras, Jamaica, Mexico, Nicaragua, Panama, Paraguay, Peru, United States, Trinidad and Tobago, Venezuela.

##### Notes

This is the first record for the State of Rio de Janeiro, which extends the known distribution eastwards.

#### Hypoponera trigona

(Mayr, 1887)

35A39CE4-F9B9-5134-99BB-23F6BBB5F62D

##### Materials

- **Type status:** Other material. **Occurrence:** recordNumber: WAC104; recordedBy: M. M. Silva, A. J. Mayhé Nunes, J. C. Santos, I. R. S. Cordeiro; individualCount: 18; sex: female; lifeStage: adult; occurrenceID: 193B7721-DD2A-535D-9AB9-4E4B6A62E795; **Taxon:** genus: Hypoponera; specificEpithet: trigona; taxonRank: species; scientificNameAuthorship: (Mayr, 1887); **Location:** continent: South America; country: Brazil; countryCode: BR; stateProvince: Rio de Janeiro; municipality: Rio de Janeiro; locality: Floresta da Tijuca, Parque Nacional da Tijuca, Bom Retiro (trilha para o pico da Tijuca); verbatimCoordinates: 22 56 48.4S 43 17 32.7W; verbatimLatitude: 22 56 48.4S; verbatimLongitude: 43 17 32.7W; verbatimCoordinateSystem: degrees minutes seconds; verbatimSRS: WGS84; **Identification:** identificationID: Hypoponeratrigona; identifiedBy: M. M. Silva; **Event:** samplingProtocol: Winckler; year: 2021; month: 7; day: 19-21; verbatimEventDate: Winter 2021; habitat: Atlantic Forest; **Record Level:** type: PhysicalObject; institutionCode: CEIOC; basisOfRecord: PreservedSpecimen
- **Type status:** Other material. **Occurrence:** recordNumber: WBS109; recordedBy: M. M. Silva, A. J. Mayhé Nunes, J. C. Santos, I. R. S. Cordeiro; individualCount: 8; sex: female; lifeStage: adult; occurrenceID: F8480DA7-FF47-562E-95E0-AF84C11E7C16; **Taxon:** genus: Hypoponera; specificEpithet: trigona; taxonRank: species; scientificNameAuthorship: (Mayr, 1887); **Location:** continent: South America; country: Brazil; countryCode: BR; stateProvince: Rio de Janeiro; municipality: Rio de Janeiro; locality: Floresta da Tijuca, Parque Nacional da Tijuca, trilha (caminho dos picos/ camminho da solidão); verbatimCoordinates: 22 57 25.3S 43 16 56.2W; verbatimLatitude: 22 57 25.3S; verbatimLongitude: 43 16 56.2W; verbatimCoordinateSystem: degrees minutes seconds; verbatimSRS: WGS84; **Identification:** identificationID: Hypoponeratrigona; identifiedBy: M. M. Silva; **Event:** samplingProtocol: Winckler; year: 2021; month: 7; day: 21-23; verbatimEventDate: Winter 2021; habitat: Atlantic Forest; **Record Level:** type: PhysicalObject; institutionCode: CEIOC; basisOfRecord: PreservedSpecimen
- **Type status:** Other material. **Occurrence:** recordNumber: WCS112; recordedBy: M. M. Silva, A. J. Mayhé Nunes, J. C. Santos, I. R. S. Cordeiro; individualCount: 14; sex: female; lifeStage: adult; occurrenceID: 40B5BCC3-60C1-5A7E-828A-5AF81C7152DF; **Taxon:** genus: Hypoponera; specificEpithet: trigona; taxonRank: species; scientificNameAuthorship: (Mayr, 1887); **Location:** continent: South America; country: Brazil; countryCode: BR; stateProvince: Rio de Janeiro; municipality: Rio de Janeiro; locality: Floresta da Tijuca, Parque Nacional da Tijuca, Trilha de Moutain bike (Açude da solidão).; verbatimCoordinates: 22 57 40.4S 43 17 28.1W; verbatimLatitude: 22 57 40.4S; verbatimLongitude: 43 17 28.1W; verbatimCoordinateSystem: degrees minutes seconds; verbatimSRS: WGS84; **Identification:** identificationID: Hypoponeratrigona; identifiedBy: M. M. Silva; **Event:** samplingProtocol: Winckler; year: 2021; month: 7; day: 23-25; verbatimEventDate: Winter 2021; habitat: Atlantic Forest; **Record Level:** type: PhysicalObject; institutionCode: CEIOC; basisOfRecord: PreservedSpecimen

##### Distribution

Argentina, Bolivia, Brazil, Colombia, Costa Rica, Ecuador, Guatemala, Mexico, Panama, Peru, Paraguay. Exotic in the Easter Island. Records from Bahamas, Cuba, Lesser Antilles and the United States need verification. Records from Juan Fernandez Islands and Society Islands are dubious.

##### Notes

This species was recorded by Kempf (1962) from "Floresta da Tijuca" without details.

#### Hypoponera viri

(Santschi, 1923)

CE5BE820-BFC5-51A9-B450-0A9123776E0C

##### Materials

- **Type status:** Other material. **Occurrence:** recordNumber: WAS102; recordedBy: M. M. Silva, A. J. Mayhé Nunes, J. C. Santos, I. R. S. Cordeiro; individualCount: 11; sex: female; lifeStage: adult; occurrenceID: 767F8FCB-6985-5E6D-B168-425A99053566; **Taxon:** genus: Hypoponera; specificEpithet: viri; taxonRank: species; scientificNameAuthorship: (Santschi, 1923); **Location:** continent: South America; country: Brazil; countryCode: BR; stateProvince: Rio de Janeiro; municipality: Rio de Janeiro; locality: Floresta da Tijuca, Parque Nacional da Tijuca, Bom Retiro (trilha para o pico da Tijuca); verbatimCoordinates: 22 56 47.5S 43 17 30.6W; verbatimLatitude: 22 56 47.5S; verbatimLongitude: 43 17 30.6W; verbatimCoordinateSystem: degrees minutes seconds; verbatimSRS: WGS84; **Identification:** identificationID: Hypoponeraviri; identifiedBy: M. M. Silva; **Event:** samplingProtocol: Winckler; year: 2021; month: 7; day: 21-23; verbatimEventDate: Winter 2021; habitat: Atlantic Forest; **Record Level:** type: PhysicalObject; institutionCode: CEIOC; basisOfRecord: PreservedSpecimen
- **Type status:** Other material. **Occurrence:** recordNumber: WBS120; recordedBy: M. M. Silva, A. J. Mayhé Nunes, J. C. Santos, I. R. S. Cordeiro; individualCount: 6; sex: female; lifeStage: adult; occurrenceID: 3EE4491A-8290-5004-BCD3-3B33DE164F27; **Taxon:** genus: Hypoponera; specificEpithet: viri; taxonRank: species; scientificNameAuthorship: (Santschi, 1923); **Location:** continent: South America; country: Brazil; countryCode: BR; stateProvince: Rio de Janeiro; municipality: Rio de Janeiro; locality: Floresta da Tijuca, Parque Nacional da Tijuca, trilha (caminho dos picos/ camminho da solidão); verbatimCoordinates: 22 57 30.0S 43 16 58.3W; verbatimLatitude: 22 57 30.0S; verbatimLongitude: 43 16 58.3W; verbatimCoordinateSystem: degrees minutes seconds; verbatimSRS: WGS84; **Identification:** identificationID: Hypoponeraviri; identifiedBy: M. M. Silva; **Event:** samplingProtocol: Winckler; year: 2021; month: 1; day: 17-20; verbatimEventDate: Summer 2021; habitat: Atlantic Forest; **Record Level:** type: PhysicalObject; institutionCode: CEIOC; basisOfRecord: PreservedSpecimen
- **Type status:** Other material. **Occurrence:** recordNumber: WBS117; recordedBy: M. M. Silva, A. J. Mayhé Nunes, J. C. Santos, I. R. S. Cordeiro; individualCount: 6; sex: female; lifeStage: adult; occurrenceID: F71A1F37-ED2C-56C0-9215-0E61A9F9C68B; **Taxon:** genus: Hypoponera; specificEpithet: viri; taxonRank: species; scientificNameAuthorship: (Santschi, 1923); **Location:** continent: South America; country: Brazil; countryCode: BR; stateProvince: Rio de Janeiro; municipality: Rio de Janeiro; locality: Floresta da Tijuca, Parque Nacional da Tijuca, Trilha de Moutain bike (Açude da solidão).; verbatimCoordinates: 22 57 40.5S 43 17 29.9W; verbatimLatitude: 22 57 40.5S; verbatimLongitude: 43 17 29.9W; verbatimCoordinateSystem: degrees minutes seconds; verbatimSRS: WGS84; **Identification:** identificationID: Hypoponeraviri; identifiedBy: M. M. Silva; **Event:** samplingProtocol: Winckler; year: 2022; month: 1; day: 17-19; verbatimEventDate: Summer 2022; habitat: Atlantic Forest; **Record Level:** type: PhysicalObject; institutionCode: CEIOC; basisOfRecord: PreservedSpecimen

##### Distribution

Endemic from eastern Brazil.

##### Notes

This is the first record for the State of Rio de Janeiro, which extends the known distribution northwards.

#### Leptogenys luederwaldti

Forel, 1913

1D2964E2-3748-5B52-A8F2-B137D079F28C

##### Materials

- **Type status:** Other material. **Occurrence:** recordNumber: PAC108; recordedBy: M. M. Silva, A. J. Mayhé Nunes, J. C. Santos, I. R. S. Cordeiro; individualCount: 2; sex: female; lifeStage: adult; occurrenceID: 545FF66F-0FD6-5C80-B3D6-181773D01F28; **Taxon:** genus: Leptogenys; specificEpithet: luederwaldti; taxonRank: species; scientificNameAuthorship: Forel, 1913; **Location:** continent: South America; country: Brazil; countryCode: BR; stateProvince: Rio de Janeiro; municipality: Rio de Janeiro; locality: Floresta da Tijuca, Parque Nacional da Tijuca, Bom Retiro (trilha para o pico da Tijuca); verbatimCoordinates: 22 56 47.2S 43 17 30.3W; verbatimLatitude: 22 56 47.2S; verbatimLongitude: 43 17 30.3W; verbatimCoordinateSystem: degrees minutes seconds; verbatimSRS: WGS84; **Identification:** identificationID: Leptogenysluederwaldti; identifiedBy: M. M. Silva; **Event:** samplingProtocol: Pitfall; year: 2022; month: 1; day: 17-19; verbatimEventDate: Summer 2022; habitat: Atlantic Forest; **Record Level:** type: PhysicalObject; institutionCode: CEIOC; basisOfRecord: PreservedSpecimen
- **Type status:** Other material. **Occurrence:** recordNumber: PCC105; recordedBy: M. M. Silva, A. J. Mayhé Nunes, J. C. Santos, I. R. S. Cordeiro; individualCount: 4; sex: female; lifeStage: adult; occurrenceID: 084EFE36-A705-5993-8E08-772B6722AA64; **Taxon:** genus: Leptogenys; specificEpithet: luederwaldti; taxonRank: species; scientificNameAuthorship: Forel, 1913; **Location:** continent: South America; country: Brazil; countryCode: BR; stateProvince: Rio de Janeiro; municipality: Rio de Janeiro; locality: Floresta da Tijuca, Parque Nacional da Tijuca, Trilha de Moutain bike (Açude da solidão).; verbatimCoordinates: 22 57 42.1S 43 17 24.7W; verbatimLatitude: 22 57 42.1S; verbatimLongitude: 43 17 24.7W; verbatimCoordinateSystem: degrees minutes seconds; verbatimSRS: WGS84; **Identification:** identificationID: Leptogenysluederwaldti; identifiedBy: M. M. Silva; **Event:** samplingProtocol: Pitfall; year: 2022; month: 1; day: 23-25; verbatimEventDate: Summer 2022; habitat: Atlantic Forest; **Record Level:** type: PhysicalObject; institutionCode: CEIOC; basisOfRecord: PreservedSpecimen

##### Distribution

Endemic from eastern Brazil.

##### Notes

This is the first record for the PNT. This species was previously recorded by Kempf (1972) and Lattke (2011) from the Municipality of Petrópolis, located approximately 70 km north of Rio de Janeiro.

#### Neoponera verenae

(Forel, 1922)

CB11E418-2F53-5D05-B946-46870AE8CCB8

##### Materials

- **Type status:** Other material. **Occurrence:** recordNumber: PCC103; recordedBy: M. M. Silva, A. J. Mayhé Nunes, J. C. Santos, I. R. S. Cordeiro; individualCount: 1; sex: female; lifeStage: adult; occurrenceID: 26DCF324-DA07-5004-BD1B-DCA65B427DAF; **Taxon:** genus: Neoponera; specificEpithet: verenae; taxonRank: species; scientificNameAuthorship: (Forel, 1922); **Location:** continent: South America; country: Brazil; countryCode: BR; stateProvince: Rio de Janeiro; municipality: Rio de Janeiro; locality: Floresta da Tijuca, Parque Nacional da Tijuca, Bom Retiro (trilha para o pico da Tijuca); verbatimCoordinates: 22 57 42.6S 43 17 23.8W; verbatimLatitude: 22 57 42.6S; verbatimLongitude: 43 17 23.8W; verbatimCoordinateSystem: degrees minutes seconds; verbatimSRS: WGS84; **Identification:** identificationID: Neoponeraverenae; identifiedBy: M. M. Silva; **Event:** samplingProtocol: Pitfall; year: 2022; month: 1; day: 17-19; verbatimEventDate: Summer 2022; habitat: Atlantic Forest; **Record Level:** type: PhysicalObject; institutionCode: CEIOC; basisOfRecord: PreservedSpecimen
- **Type status:** Other material. **Occurrence:** recordNumber: PBS104; recordedBy: M. M. Silva, A. J. Mayhé Nunes, J. C. Santos, I. R. S. Cordeiro; individualCount: 6; sex: female; lifeStage: adult; occurrenceID: 57818E70-E847-5471-A32E-0F85B1726E98; **Taxon:** genus: Neoponera; specificEpithet: verenae; taxonRank: species; scientificNameAuthorship: (Forel, 1922); **Location:** continent: South America; country: Brazil; countryCode: BR; stateProvince: Rio de Janeiro; municipality: Rio de Janeiro; locality: Floresta da Tijuca, Parque Nacional da Tijuca, Trilha de Moutain bike (Açude da solidão).; verbatimCoordinates: 22 57 23.1S 43 16 57.9W; verbatimLatitude: 22 57 23.1S; verbatimLongitude: 43 16 57.9W; verbatimCoordinateSystem: degrees minutes seconds; verbatimSRS: WGS84; **Identification:** identificationID: Neoponeraverenae; identifiedBy: M. M. Silva; **Event:** samplingProtocol: Pitfall; year: 2022; month: 1; day: 23-25; verbatimEventDate: Summer 2022; habitat: Atlantic Forest; **Record Level:** type: PhysicalObject; institutionCode: CEIOC; basisOfRecord: PreservedSpecimen

##### Distribution

Argentina, Bolivia, Brazil, Colombia, Costa Rica, Ecuador, El Salvador, French Guiana, Guatemala, Guyana, Honduras, Mexico, Nicaragua, Panama, Paraguay, Peru, Venezuela.

##### Notes

This is the first record for the PNT. Previously recorded by Apolinário et al. (2019) from the State of Rio de Janeiro.

#### Odontomachus affinis

Guérin-Méneville, 1844

B9587235-9646-5B44-B279-70E18C244FE4

##### Materials

- **Type status:** Other material. **Occurrence:** recordNumber: PAC103; recordedBy: M. M. Silva, A. J. Mayhé Nunes, J. C. Santos, I. R. S. Cordeiro; individualCount: 2; sex: female; lifeStage: adult; occurrenceID: C8F33033-D07D-57CE-B116-7DE2FBCFA76A; **Taxon:** genus: Odontomachus; specificEpithet: affinis; taxonRank: species; scientificNameAuthorship: Guérin-Méneville, 1844; **Location:** continent: South America; country: Brazil; countryCode: BR; stateProvince: Rio de Janeiro; municipality: Rio de Janeiro; locality: Floresta da Tijuca, Parque Nacional da Tijuca, Bom Retiro (trilha para o pico da Tijuca); verbatimCoordinates: 22 57 41.8S 43 17 24.8W; verbatimLatitude: 22 57 41.8S; verbatimLongitude: 43 17 24.8W; verbatimCoordinateSystem: degrees minutes seconds; verbatimSRS: WGS84; **Identification:** identificationID: Odontomachusaffinis; identifiedBy: M. M. Silva; **Event:** samplingProtocol: Pitfall; year: 2022; month: 1; day: 21-23; verbatimEventDate: Summer 2022; habitat: Atlantic Forest; **Record Level:** type: PhysicalObject; institutionCode: CEIOC; basisOfRecord: PreservedSpecimen
- **Type status:** Other material. **Occurrence:** recordNumber: PBC115; recordedBy: M. M. Silva, A. J. Mayhé Nunes, J. C. Santos, I. R. S. Cordeiro; individualCount: 5; sex: female; lifeStage: adult; occurrenceID: 85FA713A-26DE-5C50-B9DE-53DDDDF6E78C; **Taxon:** genus: Odontomachus; specificEpithet: affinis; taxonRank: species; scientificNameAuthorship: Guérin-Méneville, 1844; **Location:** continent: South America; country: Brazil; countryCode: BR; stateProvince: Rio de Janeiro; municipality: Rio de Janeiro; locality: Floresta da Tijuca, Parque Nacional da Tijuca, trilha (caminho dos picos/ camminho da solidão); verbatimCoordinates: 22 56 48.4S 43 17 32.7W; verbatimLatitude: 22 56 48.4S; verbatimLongitude: 43 17 32.7W; verbatimCoordinateSystem: degrees minutes seconds; verbatimSRS: WGS84; **Identification:** identificationID: Odontomachusaffinis; identifiedBy: M. M. Silva; **Event:** samplingProtocol: Pitfall; year: 2021; month: 7; day: 19-21; verbatimEventDate: Winter 2021; habitat: Atlantic Forest; **Record Level:** type: PhysicalObject; institutionCode: CEIOC; basisOfRecord: PreservedSpecimen
- **Type status:** Other material. **Occurrence:** recordNumber: WCC017; recordedBy: M. M. Silva, A. J. Mayhé Nunes, J. C. Santos, I. R. S. Cordeiro; individualCount: 3; sex: female; lifeStage: adult; occurrenceID: 20A12A2B-0A12-5E5E-A0E6-EB286906CC12; **Taxon:** genus: Odontomachus; specificEpithet: affinis; taxonRank: species; scientificNameAuthorship: Guérin-Méneville, 1844; **Location:** continent: South America; country: Brazil; countryCode: BR; stateProvince: Rio de Janeiro; municipality: Rio de Janeiro; locality: Floresta da Tijuca, Parque Nacional da Tijuca, Trilha de Moutain bike (Açude da solidão).; verbatimCoordinates: 22 57 25.3S 43 16 56.2W; verbatimLatitude: 22 57 25.3S; verbatimLongitude: 43 16 56.2W; verbatimCoordinateSystem: degrees minutes seconds; verbatimSRS: WGS84; **Identification:** identificationID: Odontomachusaffinis; identifiedBy: M. M. Silva; **Event:** samplingProtocol: Winckler; year: 2021; month: 7; day: 21-23; verbatimEventDate: Winter 2021; habitat: Atlantic Forest; **Record Level:** type: PhysicalObject; institutionCode: CEIOC; basisOfRecord: PreservedSpecimen

##### Distribution

Brazil, Colombia, Guyana.

##### Notes

This is the first record for the PNT. Previously recorded by Kempf (1972) from the State of Rio de Janeiro without details.

#### Odontomachus brunneus

(Patton, 1894)

F97A78C8-4F6E-5CD9-808C-E0A965DC7B74

##### Materials

- **Type status:** Other material. **Occurrence:** recordNumber: WBS111; recordedBy: M. M. Silva, A. J. Mayhé Nunes, J. C. Santos, I. R. S. Cordeiro; individualCount: 1; sex: female; lifeStage: adult; occurrenceID: 87D958DE-D8E7-5FEE-87A7-D0634FCCF44B; **Taxon:** genus: Odontomachus; specificEpithet: brunneus; taxonRank: species; scientificNameAuthorship: (Patton, 1894); **Location:** continent: South America; country: Brazil; countryCode: BR; stateProvince: Rio de Janeiro; municipality: Rio de Janeiro; locality: Floresta da Tijuca, Parque Nacional da Tijuca, trilha (caminho dos picos/ camminho da solidão); verbatimCoordinates: 22 56 46.8S 43 17 33.6W; verbatimLatitude: 22 56 46.8S; verbatimLongitude: 43 17 33.6W; verbatimCoordinateSystem: degrees minutes seconds; verbatimSRS: WGS84; **Identification:** identificationID: Odontomachusbrunneus; identifiedBy: M. M. Silva; **Event:** samplingProtocol: Winckler; year: 2021; month: 7; day: 19-21; verbatimEventDate: Winter 2021; habitat: Atlantic Forest; **Record Level:** type: PhysicalObject; institutionCode: CEIOC; basisOfRecord: PreservedSpecimen
- **Type status:** Other material. **Occurrence:** recordNumber: WCS115; recordedBy: M. M. Silva, A. J. Mayhé Nunes, J. C. Santos, I. R. S. Cordeiro; individualCount: 1; sex: female; lifeStage: adult; occurrenceID: 1898AB55-379C-50BE-8225-6B937B47534C; **Taxon:** genus: Odontomachus; specificEpithet: brunneus; taxonRank: species; scientificNameAuthorship: (Patton, 1894); **Location:** continent: South America; country: Brazil; countryCode: BR; stateProvince: Rio de Janeiro; municipality: Rio de Janeiro; locality: Floresta da Tijuca, Parque Nacional da Tijuca, Trilha de Moutain bike (Açude da solidão).; verbatimCoordinates: 22 56 47.8S 43 17 30.4W; verbatimLatitude: 22 56 47.8S; verbatimLongitude: 43 17 30.4W; verbatimCoordinateSystem: degrees minutes seconds; verbatimSRS: WGS84; **Identification:** identificationID: Odontomachusbrunneus; identifiedBy: M. M. Silva; **Event:** samplingProtocol: Winckler; year: 2021; month: 7; day: 21-23; verbatimEventDate: Winter 2021; habitat: Atlantic Forest; **Record Level:** type: PhysicalObject; institutionCode: CEIOC; basisOfRecord: PreservedSpecimen

##### Distribution

Mexico, United States. Introduced to the Netherlands. Most existing records throughout the Neotropical Region need verification.

##### Notes

This is the first record from the PNT. Previously recorded by Silva and Brandão (2014) from other protected areas in the State of Rio de Janeiro.

#### Odontomachus chelifer

(Latreille, 1802)

6B5D8D9E-AFAB-5BAB-8877-DA2685D183A1

##### Materials

- **Type status:** Other material. **Occurrence:** recordNumber: PAC111; recordedBy: M. M. Silva, A. J. Mayhé Nunes, J. C. Santos, I. R. S. Cordeiro; individualCount: 2; sex: female; lifeStage: adult; occurrenceID: CA07126C-95A0-5516-B232-CE6E6D8E1221; **Taxon:** genus: Odontomachus; specificEpithet: chelifer; taxonRank: species; scientificNameAuthorship: (Latreille, 1802); **Location:** continent: South America; country: Brazil; countryCode: BR; stateProvince: Rio de Janeiro; municipality: Rio de Janeiro; locality: Floresta da Tijuca, Parque Nacional da Tijuca, Bom Retiro (trilha para o pico da Tijuca); verbatimCoordinates: 22 57 42.0S 43 17 23.9W; verbatimLatitude: 22 57 42.0S; verbatimLongitude: 43 17 23.9W; verbatimCoordinateSystem: degrees minutes seconds; verbatimSRS: WGS84; **Identification:** identificationID: Odontomachuschelifer; identifiedBy: M. M. Silva; **Event:** samplingProtocol: Pitfall; year: 2021; month: 7; day: 19-21; verbatimEventDate: Winter 2021; habitat: Atlantic Forest; **Record Level:** type: PhysicalObject; institutionCode: CEIOC; basisOfRecord: PreservedSpecimen
- **Type status:** Other material. **Occurrence:** recordNumber: PBS117; recordedBy: M. M. Silva, A. J. Mayhé Nunes, J. C. Santos, I. R. S. Cordeiro; individualCount: 3; sex: female; lifeStage: adult; occurrenceID: AE736F2E-8C7F-55D9-896B-0DAC507C75DD; **Taxon:** genus: Odontomachus; specificEpithet: chelifer; taxonRank: species; scientificNameAuthorship: (Latreille, 1802); **Location:** continent: South America; country: Brazil; countryCode: BR; stateProvince: Rio de Janeiro; municipality: Rio de Janeiro; locality: Floresta da Tijuca, Parque Nacional da Tijuca, trilha (caminho dos picos/ camminho da solidão); verbatimCoordinates: 22 56 48.2S 43 17 29.8W; verbatimLatitude: 22 56 48.2S; verbatimLongitude: 43 17 29.8W; verbatimCoordinateSystem: degrees minutes seconds; verbatimSRS: WGS84; **Identification:** identificationID: Odontomachuschelifer; identifiedBy: M. M. Silva; **Event:** samplingProtocol: Pitfall; year: 2021; month: 7; day: 21-23; verbatimEventDate: Winter 2021; habitat: Atlantic Forest; **Record Level:** type: PhysicalObject; institutionCode: CEIOC; basisOfRecord: PreservedSpecimen
- **Type status:** Other material. **Occurrence:** recordNumber: PCS101; recordedBy: M. M. Silva, A. J. Mayhé Nunes, J. C. Santos, I. R. S. Cordeiro; individualCount: 2; sex: female; lifeStage: adult; occurrenceID: 70F43E70-E844-5BAA-AB6F-3938F90AEA9A; **Taxon:** genus: Odontomachus; specificEpithet: chelifer; taxonRank: species; scientificNameAuthorship: (Latreille, 1802); **Location:** continent: South America; country: Brazil; countryCode: BR; stateProvince: Rio de Janeiro; municipality: Rio de Janeiro; locality: Floresta da Tijuca, Parque Nacional da Tijuca, Trilha de Moutain bike (Açude da solidão).; verbatimCoordinates: 22 56 47.7S 43 17 33.1W; verbatimLatitude: 22 56 47.7S; verbatimLongitude: 43 17 33.1W; verbatimCoordinateSystem: degrees minutes seconds; verbatimSRS: WGS84; **Identification:** identificationID: Odontomachuschelifer; identifiedBy: M. M. Silva; **Event:** samplingProtocol: Pitfall; year: 2021; month: 7; day: 23-25; verbatimEventDate: Winter 2021; habitat: Atlantic Forest; **Record Level:** type: PhysicalObject; institutionCode: CEIOC; basisOfRecord: PreservedSpecimen

##### Distribution

Argentina, Bolivia, Brazil, Colombia, Costa Rica, Ecuador, French Guiana, Guatemala, Guyana, Honduras, Mexico, Nicaragua, Panama, Paraguay, Peru, Suriname, Trinidad and Tobago, Venezuela.

##### Notes

This is the first record from the Floresta da Tijuca sector. This species was recorded by Vargas et al. (2013) and Santos et al. (2019) from the Serra da Carioca sector.

#### Odontomachus meinerti

Forel, 1905

96EC39C0-FBDD-5E3E-BE64-12805E52B651

##### Materials

- **Type status:** Other material. **Occurrence:** recordNumber: PAC114; recordedBy: M. M. Silva, A. J. Mayhé Nunes, J. C. Santos, I. R. S. Cordeiro; individualCount: 1; sex: female; lifeStage: adult; occurrenceID: 67046DA2-8D2E-5251-9BE6-6BD2C1CE3E7F; **Taxon:** genus: Odontomachus; specificEpithet: meinerti; taxonRank: species; scientificNameAuthorship: Forel, 1905; **Location:** continent: South America; country: Brazil; countryCode: BR; stateProvince: Rio de Janeiro; municipality: Rio de Janeiro; locality: Floresta da Tijuca, Parque Nacional da Tijuca, Bom Retiro (trilha para o pico da Tijuca); verbatimCoordinates: 22 56 46.2S 43 17 32.7W; verbatimLatitude: 22 56 46.2S; verbatimLongitude: 43 17 32.7W; verbatimCoordinateSystem: degrees minutes seconds; verbatimSRS: WGS84; **Identification:** identificationID: Odontomachusmeinerti; identifiedBy: M. M. Silva; **Event:** samplingProtocol: Pitfall; year: 2022; month: 1; day: 17-19; verbatimEventDate: Summer 2022; habitat: Atlantic Forest; **Record Level:** type: PhysicalObject; institutionCode: CEIOC; basisOfRecord: PreservedSpecimen
- **Type status:** Other material. **Occurrence:** recordNumber: WBS119; recordedBy: M. M. Silva, A. J. Mayhé Nunes, J. C. Santos, I. R. S. Cordeiro; individualCount: 5; sex: female; lifeStage: adult; occurrenceID: D2330F84-4B69-5835-B558-4079DB4DEB6F; **Taxon:** genus: Odontomachus; specificEpithet: meinerti; taxonRank: species; scientificNameAuthorship: Forel, 1905; **Location:** continent: South America; country: Brazil; countryCode: BR; stateProvince: Rio de Janeiro; municipality: Rio de Janeiro; locality: Floresta da Tijuca, Parque Nacional da Tijuca, trilha (caminho dos picos/ camminho da solidão); verbatimCoordinates: 22 56 48.2S 43 17 29.8W; verbatimLatitude: 22 56 48.2S; verbatimLongitude: 43 17 29.8W; verbatimCoordinateSystem: degrees minutes seconds; verbatimSRS: WGS84; **Identification:** identificationID: Odontomachusmeinerti; identifiedBy: M. M. Silva; **Event:** samplingProtocol: Pitfall; year: 2021; month: 7; day: 21-23; verbatimEventDate: Winter 2021; habitat: Atlantic Forest; **Record Level:** type: PhysicalObject; institutionCode: CEIOC; basisOfRecord: PreservedSpecimen
- **Type status:** Other material. **Occurrence:** recordNumber: WCS104; recordedBy: M. M. Silva, A. J. Mayhé Nunes, J. C. Santos, I. R. S. Cordeiro; individualCount: 3; sex: female; lifeStage: adult; occurrenceID: 108BC928-4BED-510C-9898-843BD16CA1BD; **Taxon:** genus: Odontomachus; specificEpithet: meinerti; taxonRank: species; scientificNameAuthorship: Forel, 1905; **Location:** continent: South America; country: Brazil; countryCode: BR; stateProvince: Rio de Janeiro; municipality: Rio de Janeiro; locality: Floresta da Tijuca, Parque Nacional da Tijuca, Trilha de Moutain bike (Açude da solidão).; verbatimCoordinates: 22 57 23.2S 43 16 57.7W; verbatimLatitude: 22 57 23.2S; verbatimLongitude: 43 16 57.7W; verbatimCoordinateSystem: degrees minutes seconds; verbatimSRS: WGS84; **Identification:** identificationID: Odontomachusmeinerti; identifiedBy: M. M. Silva; **Event:** samplingProtocol: Pitfall; year: 2021; month: 7; day: 23-25; verbatimEventDate: Winter 2021; habitat: Atlantic Forest; **Record Level:** type: PhysicalObject; institutionCode: CEIOC; basisOfRecord: PreservedSpecimen

##### Distribution

Argentina, Bolivia, Brazil, Colombia, Costa Rica, Ecuador, French Guiana, Guatemala, Guyana, Honduras, Mexico, Nicaragua, Panama, Paraguay, Peru, Trinidad and Tobago, Venezuela.

##### Notes

This is the first record from the Floresta da Tijuca sector. Previously recorded by Vargas et al. (2013) from the Serra da Carioca sector.

#### Pachycondyla harpax

(Fabricius, 1804)

D02B2294-2747-5A31-94CB-3AC1E0FB29E7

##### Materials

- **Type status:** Other material. **Occurrence:** recordNumber: PAS202; recordedBy: M. M. Silva, A. J. Mayhé Nunes, J. C. Santos, I. R. S. Cordeiro; individualCount: 2; sex: female; lifeStage: adult; occurrenceID: D0556847-A556-5E03-9FD4-AB546ABBDB0B; **Taxon:** genus: Pachycondyla; specificEpithet: harpax; taxonRank: species; scientificNameAuthorship: (Fabricius, 1804); **Location:** continent: South America; country: Brazil; countryCode: BR; stateProvince: Rio de Janeiro; municipality: Rio de Janeiro; locality: Floresta da Tijuca, Parque Nacional da Tijuca, Bom Retiro (trilha para o pico da Tijuca); verbatimCoordinates: 22 57 27.0S 43 16 55.4W; verbatimLatitude: 22 57 27.0S; verbatimLongitude: 43 16 55.4W; verbatimCoordinateSystem: degrees minutes seconds; verbatimSRS: WGS84; **Identification:** identificationID: Pachycondylaharpax; identifiedBy: M. M. Silva; **Event:** samplingProtocol: Pitfall; year: 2022; month: 1; day: 17-21; verbatimEventDate: Summer 2022; habitat: Atlantic Forest; **Record Level:** type: PhysicalObject; institutionCode: CEIOC; basisOfRecord: PreservedSpecimen
- **Type status:** Other material. **Occurrence:** recordNumber: WBS112; recordedBy: M. M. Silva, A. J. Mayhé Nunes, J. C. Santos, I. R. S. Cordeiro; individualCount: 2; sex: female; lifeStage: adult; occurrenceID: E175E677-7C91-58B0-9D61-55DBC7D9CA75; **Taxon:** genus: Pachycondyla; specificEpithet: harpax; taxonRank: species; scientificNameAuthorship: (Fabricius, 1804); **Location:** continent: South America; country: Brazil; countryCode: BR; stateProvince: Rio de Janeiro; municipality: Rio de Janeiro; locality: Floresta da Tijuca, Parque Nacional da Tijuca, trilha (caminho dos picos/ camminho da solidão); verbatimCoordinates: 22 57 40.0S 43 17 28.5W; verbatimLatitude: 22 57 40.0S; verbatimLongitude: 43 17 28.5W; verbatimCoordinateSystem: degrees minutes seconds; verbatimSRS: WGS84; **Identification:** identificationID: Pachycondylaharpax; identifiedBy: M. M. Silva; **Event:** samplingProtocol: Pitfall; year: 2021; month: 7; day: 19-21; verbatimEventDate: Winter 2021; habitat: Atlantic Forest; **Record Level:** type: PhysicalObject; institutionCode: CEIOC; basisOfRecord: PreservedSpecimen
- **Type status:** Other material. **Occurrence:** recordNumber: PCS102; recordedBy: M. M. Silva, A. J. Mayhé Nunes, J. C. Santos, I. R. S. Cordeiro; individualCount: 15; sex: female; lifeStage: adult; occurrenceID: 2EE6E05B-180D-5760-BB18-92A5E9B89A3B; **Taxon:** genus: Pachycondyla; specificEpithet: harpax; taxonRank: species; scientificNameAuthorship: (Fabricius, 1804); **Location:** continent: South America; country: Brazil; countryCode: BR; stateProvince: Rio de Janeiro; municipality: Rio de Janeiro; locality: Floresta da Tijuca, Parque Nacional da Tijuca, Trilha de Moutain bike (Açude da solidão).; verbatimCoordinates: 22 56 45.6S 43 17 32.5W; verbatimLatitude: 22 56 45.6S; verbatimLongitude: 43 17 32.5W; verbatimCoordinateSystem: degrees minutes seconds; verbatimSRS: WGS84; **Identification:** identificationID: Pachycondylaharpax; identifiedBy: M. M. Silva; **Event:** samplingProtocol: Pitfall; year: 2021; month: 7; day: 21-23; verbatimEventDate: Winter 2021; habitat: Atlantic Forest; **Record Level:** type: PhysicalObject; institutionCode: CEIOC; basisOfRecord: PreservedSpecimen

##### Distribution

Argentina, Belize, Bolivia, Brazil, Colombia, Costa Rica, Ecuador, El Salvador, French Guiana, Guatemala, Guyana, Honduras, Mexico, Nicaragua, Panama, Paraguay, Peru, Suriname, Trinidad and Tobago, United States, Venezuela. Exotic in Jamaica, Lesser Antilles and the State of Georgia (United States).

##### Notes

This is the first record from the Floresta da Tijuca sector. Previously recorded by Vargas et al. (2013) and Santos et al. (2019) from the Serra da Carioca sector.

#### Pachycondyla striata

Smith, F., 1858

17F319AE-6B77-5F35-94EB-FF83B3BEFD05

##### Materials

- **Type status:** Other material. **Occurrence:** recordNumber: PAC101; recordedBy: M. M. Silva, A. J. Mayhé Nunes, J. C. Santos, I. R. S. Cordeiro; individualCount: 19; sex: female; lifeStage: adult; occurrenceID: 52D1C397-506B-5E86-BDA3-46A44C87E094; **Taxon:** genus: Pachycondyla; specificEpithet: striata; taxonRank: species; scientificNameAuthorship: Smith, F., 1858; **Location:** continent: South America; country: Brazil; countryCode: BR; stateProvince: Rio de Janeiro; municipality: Rio de Janeiro; locality: Floresta da Tijuca, Parque Nacional da Tijuca, Bom Retiro (trilha para o pico da Tijuca); verbatimCoordinates: 22 57 2.0S 43 16 59.5W; verbatimLatitude: 22 57 2.0S; verbatimLongitude: 43 16 59.5W; verbatimCoordinateSystem: degrees minutes seconds; verbatimSRS: WGS84; **Identification:** identificationID: Pachycondylastriata; identifiedBy: M. M. Silva; **Event:** samplingProtocol: Pitfall; year: 2021; month: 7; day: 21-23; verbatimEventDate: Winter 2021; habitat: Atlantic Forest; **Record Level:** type: PhysicalObject; institutionCode: CEIOC; basisOfRecord: PreservedSpecimen
- **Type status:** Other material. **Occurrence:** recordNumber: PBS102; recordedBy: M. M. Silva, A. J. Mayhé Nunes, J. C. Santos, I. R. S. Cordeiro; individualCount: 13; sex: female; lifeStage: adult; occurrenceID: FA31CA90-3DF0-59A4-A5DF-D89F4B6766D3; **Taxon:** genus: Pachycondyla; specificEpithet: striata; taxonRank: species; scientificNameAuthorship: Smith, F., 1858; **Location:** continent: South America; country: Brazil; countryCode: BR; stateProvince: Rio de Janeiro; municipality: Rio de Janeiro; locality: Floresta da Tijuca, Parque Nacional da Tijuca, trilha (caminho dos picos/ camminho da solidão); verbatimCoordinates: 22 56 43.3S 43 17 28.9W; verbatimLatitude: 22 56 43.3S; verbatimLongitude: 43 17 28.9W; verbatimCoordinateSystem: degrees minutes seconds; verbatimSRS: WGS84; **Identification:** identificationID: Pachycondylastriata; identifiedBy: M. M. Silva; **Event:** samplingProtocol: Pitfall; year: 2021; month: 1; day: 17-20; verbatimEventDate: Summer 2021; habitat: Atlantic Forest; **Record Level:** type: PhysicalObject; institutionCode: CEIOC; basisOfRecord: PreservedSpecimen
- **Type status:** Other material. **Occurrence:** recordNumber: PCS110; recordedBy: M. M. Silva, A. J. Mayhé Nunes, J. C. Santos, I. R. S. Cordeiro; individualCount: 10; sex: female; lifeStage: adult; occurrenceID: 94188DEB-7276-5EBD-9F6A-47C1C71C0D06; **Taxon:** genus: Pachycondyla; specificEpithet: striata; taxonRank: species; scientificNameAuthorship: Smith, F., 1858; **Location:** continent: South America; country: Brazil; countryCode: BR; stateProvince: Rio de Janeiro; municipality: Rio de Janeiro; locality: Floresta da Tijuca, Parque Nacional da Tijuca, Trilha de Moutain bike (Açude da solidão).; verbatimCoordinates: 22 57 24.0S 43 16 59.9W; verbatimLatitude: 22 57 24.0S; verbatimLongitude: 43 16 59.9W; verbatimCoordinateSystem: degrees minutes seconds; verbatimSRS: WGS84; **Identification:** identificationID: Pachycondylastriata; identifiedBy: M. M. Silva; **Event:** samplingProtocol: Pitfall; year: 2022; month: 1; day: 17-19; verbatimEventDate: Summer 2022; habitat: Atlantic Forest; **Record Level:** type: PhysicalObject; institutionCode: CEIOC; basisOfRecord: PreservedSpecimen

##### Distribution

Argentina, Bolivia, Brazil, Colombia, Ecuador, French Guiana, Panama, Paraguay, Peru, Uruguay, Venezuela.

##### Notes

This is the first record from the PNT. Previously recorded by Kempf (1960), Freitas et al. (2006), Salinas (2010) and Mackay and Mackay (2010) from the State of Rio de Janeiro without details.

#### Proceratiinae

Emery, 1895

54B2872E-A3F4-503B-8602-97BF0CDF1AA2

#### Discothyrea neotropica

Bruch, 1919

131E6E3B-7E04-59F1-8FA4-B58026A2E62B

##### Materials

- **Type status:** Other material. **Occurrence:** recordNumber: WAS101; recordedBy: M. M. Silva, A. J. Mayhé Nunes, J. C. Santos, I. R. S. Cordeiro; individualCount: 4; sex: female; lifeStage: adult; occurrenceID: A0DA1401-B5CB-587D-BEDA-B67A5CE898E9; **Taxon:** genus: Discothyrea; specificEpithet: neotropica; taxonRank: species; scientificNameAuthorship: Bruch, 1919; **Location:** continent: South America; country: Brazil; countryCode: BR; stateProvince: Rio de Janeiro; municipality: Rio de Janeiro; locality: Floresta da Tijuca, Parque Nacional da Tijuca, Bom Retiro (trilha para o pico da Tijuca); verbatimCoordinates: 22 56 47.5S 43 17 30.6W; verbatimLatitude: 22 56 47.5S; verbatimLongitude: 43 17 30.6W; verbatimCoordinateSystem: degrees minutes seconds; verbatimSRS: WGS84; **Identification:** identificationID: Discothyreaneotropica; identifiedBy: M. M. Silva; **Event:** samplingProtocol: Winckler; year: 2021; month: 7; day: 19-21; verbatimEventDate: Winter 2021; habitat: Atlantic Forest; **Record Level:** type: PhysicalObject; institutionCode: CEIOC; basisOfRecord: PreservedSpecimen
- **Type status:** Other material. **Occurrence:** recordNumber: WBS113; recordedBy: M. M. Silva, A. J. Mayhé Nunes, J. C. Santos, I. R. S. Cordeiro; individualCount: 1; sex: female; lifeStage: adult; occurrenceID: 83A6D77F-F9E7-5F08-88F9-F5BD64127EF0; **Taxon:** genus: Discothyrea; specificEpithet: neotropica; taxonRank: species; scientificNameAuthorship: Bruch, 1919; **Location:** continent: South America; country: Brazil; countryCode: BR; stateProvince: Rio de Janeiro; municipality: Rio de Janeiro; locality: Floresta da Tijuca, Parque Nacional da Tijuca, trilha (caminho dos picos/ camminho da solidão); verbatimCoordinates: 22 57 27.8S 43 16 57.3W; verbatimLatitude: 22 57 27.8S; verbatimLongitude: 43 16 57.3W; verbatimCoordinateSystem: degrees minutes seconds; verbatimSRS: WGS84; **Identification:** identificationID: Discothyreaneotropica; identifiedBy: M. M. Silva; **Event:** samplingProtocol: Winckler; year: 2021; month: 7; day: 21-23; verbatimEventDate: Winter 2021; habitat: Atlantic Forest; **Record Level:** type: PhysicalObject; institutionCode: CEIOC; basisOfRecord: PreservedSpecimen

##### Distribution

Argentina, Brazil, Paraguay. Records from Venezuela need verification.

##### Notes

New species record for Floresta da Tijuca. This species was reported by Kempf (1972) from Rio de Janeiro, but its locality was not described.

## Analysis

**Figure 2.**
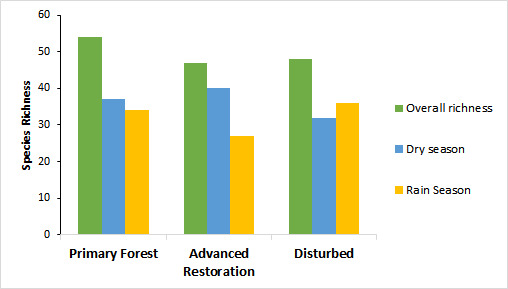
Ant species richness in the Floresta da Tijuca sector, Parque Nacional da Tijuca. The primary forest area exhibited the highest overall species richness, with the area in advanced restoration showing the highest richness during the dry season and the disturbed area being the richest during the rainy season.

We sorted 3,095 specimens of ants, identified as 80 species belonging to eight subfamilies (Table 1). The area with the highest species richness was the PF zone with 54 species, followed by DTBD and ARS zones with 48 and 47 species, respectively. During the dry season, ARS was the area with the greatest richness, totalling 40 species. In the rainy season, however, the highest richness was found in the DTBD zone (Fig. 2).

The most frequent species across all three sampled areas was *Holcoponeramoelleri*, with relative frequencies of 17.5% in PF, 21% in ARS and 28.7% in DTBD. Other species with high relative frequency in the PF area include *Pachycondylastriata* (23.7%), *Hypoponeratrigona* (20%) and *Hypoponeraforeli* (15%). In the ARS area, the most frequent species were *Strumigenysdenticulata* (18.7%), *Megalomyrmexgoeldii* (16.2%) and *P.striata* (16.2%), while *Pachycondylaharpax* (18.7%), *Wasmanniaauropunctata* (18.7%) and *S.denticulata* (16.2%) were more frequent in the DTBD zone.

Our results also increase the number of ant species recorded from the PNT from 149 to 200, while the known richness of the Floresta da Tijuca sector is expanded from 31 to 50. Furthermore, 10 species are recorded for the first time from the State of Rio de Janeiro and one from the Atlantic Forest biome (see notes in the species list above for details). The known range of *Hypoponeraviri* is extended northwards, while those of *Holcoponeramina* and *Neocerapachysneotropicus* Weber, 1939 are extended southwards and *Brachymyrmexbruchi* Forel, 1912 and *Hypoponeraparva* (Forel, 1909) are extended eastwards.

## Discussion

This work contributes to understanding the myrmecofauna of the PNT more comprehensively. We focused our efforts on the Floresta da Tijuca sector, while several published studies Borgmeier (1927), Kempf (1960), Kempf (1972), Brandão (1991), Klingenberg and Brandão (2005), Araújo et al. (2007), Veiga-Ferreira et al. (2010), Orsolon-Souza et al. (2011), Vargas et al. (2013), Silva and Brandão (2014), Santos et al. (2019)(Borgmeier 1927, Kempf 1960, Kempf 1972, Brandão 1991, Klingenberg and Brandão 2005, Araújo et al. 2007, Veiga-Ferreira et al. 2010, Orsolon-Souza et al. 2011, Vargas et al. 2013, Silva and Brandão 2020, Santos et al. 2019 and Silva et al. 2022) aimed at different parts of the Serra da Carioca sector, where urban attractions, such as the Parque Lage, Rio de Janeiro Botanical Garden and Christ the Redeemer, are located. The species richness in the Serra da Carioca sector is similar to what was observed in this study, with an average of approximately 80 species. However, despite sharing some species with the Floresta da Tijuca sector, the composition of species recorded from Serra da Carioca is slightly different. One factor that can contribute to this difference is the vegetation structure and stability of communities, as areas in the Serra da Carioca sector can suffer more direct impacts from urbanisation (i.e. pollution, fires and deforestation). Throughout the history of the PNT, these areas have undergone modifications that have certainly impacted the local fauna (i.e. locally extinct species, introduced opportunistic species, removed native forest) (Mulinge 2023). Forest cover, regardless of the stage of preservation, is a factor that has a great influence on ant abundance and diversity (Martins et al. 2022), due to its role in establishing interactions and ecological services that are fundamental to the balance of associated ecosystems (Freitas et al. 2006 and Martins et al. 2022).

The majority of species in the Floresta da Tijuca sector belong to the generalist predator functional group, according to Koch et al. (2019). This suggests that they have high ecological adaptability, enabling them to survive in the three different vegetation conditions that were studied. This is particularly true for species such as *Labiduspraedator* and *Wasmanniaauropunctata*, which are good indicators of environments influenced by human activity or undergoing restoration. These species can tolerate ecological disturbances and adapt to peri-urban environments where they can commonly be found (Orivel et al. 2009, Rosumek 2017, Tibcherani et al. 2018 and Santos et al. 2019).Orivel et al. 2009, Rosumek 2017, [Bibr B11070750][Bibr B10215668]

The many new records and distribution expansions that we discovered during this study, in different levels (for the sector, the PNT, Rio de Janeiro or even the Atlantic Forest), highlight the importance of establishing and effectively managing protected areas in cities and how inventories in such areas can decrease some of the main biodiversity knowledge shortfalls that hinder effective conservation actions (Agosti et al. 2000, Hortal et al. 2015, Borowiec 2016, Janicki et al. 2016, Guénard 2017 and Silva et al. 2022). Our work also increases the ant species richness recorded from the PNT by more than 25% and demonstrates how important this protected area is in conserving a diverse myrmecofauna and probably also other groups of terrestrial invertebrates.

### Conclusion

This work contributes to our understanding of the ant population in the study area and the PNT as a whole. Many of the species we identified had never been documented in the Floresta da Tijuca sector or the PNT. Additionally, a few species are herein reported from the State of Rio de Janeiro for the first time and one from the Atlantic Forest. However, most terrestrial invertebrates in this protected area have not been thoroughly studied. It is crucial to make new efforts to study the invertebrate fauna in the region, focusing especially on discovering primary biodiversity data and applying it in conservation planning.

## Supplementary Material

XML Treatment for Formicidae

XML Treatment for Amblyoponinae

XML Treatment for Fulakora elongata

XML Treatment for Dolichoderinae

XML Treatment for Linepithema angulatum

XML Treatment for Linepithema micans

XML Treatment for Dorylinae

XML Treatment for Labidus coecus

XML Treatment for Labidus praedator

XML Treatment for Neocerapachys neotropicus

XML Treatment for Ectatomminae

XML Treatment for Ectatomma edentatum

XML Treatment for Ectatomma muticum

XML Treatment for Ectatomma permagnum

XML Treatment for Gnamptogenys continua

XML Treatment for Gnamptogenys horni

XML Treatment for Heteroponera dentinodis

XML Treatment for Heteroponera dolo

XML Treatment for Heteroponera mayri

XML Treatment for Holcoponera mina

XML Treatment for Holcoponera moelleri

XML Treatment for Typhlomyrmex lavra

XML Treatment for Formicinae

XML Treatment for Acropyga decedens

XML Treatment for Brachymyrmex admotus

XML Treatment for Brachymyrmex australis

XML Treatment for Brachymyrmex bruchi

XML Treatment for Brachymyrmex degener

XML Treatment for Camponotus atriceps

XML Treatment for Myrmelachista bettinae

XML Treatment for Nylanderia docilis

XML Treatment for Nylanderia fulva

XML Treatment for Nylanderia goeldii

XML Treatment for Nylanderia guatemalensis

XML Treatment for Nylanderia steinheili

XML Treatment for Myrmicinae

XML Treatment for Acanthognathus brevicornis

XML Treatment for Acromyrmex brunneus

XML Treatment for Acromyrmex molestans

XML Treatment for Acromyrmex subterraneus

XML Treatment for Apterostigma ierense

XML Treatment for Apterostigma pilosum

XML Treatment for Basiceros disciger

XML Treatment for Cardiocondyla minutior

XML Treatment for Carebara brevipilosa

XML Treatment for Crematogaster montana

XML Treatment for Crematogaster nigropilosa

XML Treatment for Cyphomyrmex minutus

XML Treatment for Cyphomyrmex rimosus

XML Treatment for Hylomyrma reitteri

XML Treatment for Lachnomyrmex plaumanni

XML Treatment for Megalomyrmex goeldii

XML Treatment for Mycetomoellerius oetkeri

XML Treatment for Octostruma iheringi

XML Treatment for Octostruma rugifera

XML Treatment for Oxyepoecus myops

XML Treatment for Pheidole tijucana

XML Treatment for Rogeria lacertosa

XML Treatment for Sericomyrmex bondari

XML Treatment for Sericomyrmex saussurei

XML Treatment for Strumigenys cosmostela

XML Treatment for Strumigenys crassicornis

XML Treatment for Strumigenys denticulata

XML Treatment for Strumigenys elongata

XML Treatment for Strumigenys hindenburgi

XML Treatment for Strumigenys subedentata

XML Treatment for Paratrachymyrmex cornetzi

XML Treatment for Wasmannia affinis

XML Treatment for Wasmannia auropunctata

XML Treatment for Wasmannia lutzi

XML Treatment for Pseudomyrmicinae

XML Treatment for Pseudomyrmex schuppi

XML Treatment for Ponerinae

XML Treatment for Anochetus mayri

XML Treatment for Hypoponera distinguenda

XML Treatment for Hypoponera foreli

XML Treatment for Hypoponera leveillei

XML Treatment for Hypoponera parva

XML Treatment for Hypoponera trigona

XML Treatment for Hypoponera viri

XML Treatment for Leptogenys luederwaldti

XML Treatment for Neoponera verenae

XML Treatment for Odontomachus affinis

XML Treatment for Odontomachus brunneus

XML Treatment for Odontomachus chelifer

XML Treatment for Odontomachus meinerti

XML Treatment for Pachycondyla harpax

XML Treatment for Pachycondyla striata

XML Treatment for Proceratiinae

XML Treatment for Discothyrea neotropica

## Figures and Tables

**Table 1.** Frequency (number of positive samples per area, regardless of the collecting method) of ant species collected in the Floresta da Tijuca sector, Parque Nacional da Tijuca.

**Subfamily, species**	**Sampling areas**			
	**Primary Forest**	**Advanced Restoration**	**Disturbed**	**Total**
**Amblyoponinae**				
*Fulakoraelongata* (Santschi, 1912)	-	-	1	1
**Dolichoderinae**				
*Linepithemaangulatum* (Emery, 1894)	-	1	-	1
*Linepithemamicans* (Forel, 1908)	2	1	-	3
**Dorylinae**				
*Labiduscoecus* (Latreille, 1802)	2	-	-	2
*Labiduspraedator* (Smith, F., 1858)	-	-	6	6
*Neocerapachysneotropicus* Weber, 1939	-	-	1	1
**Ectatomminae**				
*Ectatommaedentatum* Roger, 1863	-	4	-	4
*Ectatommamuticum* Mayr, 1870	-	2	-	2
*Ectatommapermagnum* Forel, 1908	-	1	-	1
*Gnamptogenyscontinua* (Mayr, 1887)	2	-	1	3
*Gnamptogenyshorni* (Santschi, 1929)	2	-	1	3
*Heteroponeradentinodis* (Mayr, 1887)	1	-	-	1
*Heteroponeradolo* (Roger, 1860)	7	-	1	8
*Heteroponeramayri* Kempf, 1962	2	1	-	3
*Holcoponeramina* Brown, 1956	2	-	-	2
*Holcoponeramoelleri* (Forel, 1912)	14	17	23	54
*Typhlomyrmexlavra* (Lattke, 2002)	1	2	1	4
**Formicinae**				
*Acropygadecedens* (Mayr, 1887)	1	-	-	1
*Brachymyrmexadmotus* Mayr, 1887	6	3	-	9
*Brachymyrmexaustralis* Forel, 1901	4	5	1	10
*Brachymyrmexbruchi* Forel, 1912	1	-	-	1
*Brachymyrmexdegener* Emery, 1906	1	-	-	1
*Camponotusatriceps* (Smith, F., 1858)	4	1	2	7
*Myrmelachistabettinae* Forel, 1903	-	1	-	1
*Nylanderiadocilis* (Forel, 1908)	-	2	8	10
*Nylanderiafulva* (Mayr, 1862)	-	1	-	1
*Nylanderiagoeldii* (Forel, 1912)	1	2	-	3
*Nylanderiaguatemalensis* (Forel, 1885)	1	2	-	3
*Nylanderiasteinheili* (Forel, 1893)	1	5	1	7
**Myrmicinae**				
*Acanthognathusbrevicornis* Smith, 1944	4	-	9	13
*Acromyrmexbrunneus* (Forel, 1912)	-	-	4	4
*Acromyrmexmolestans* Santschi, 1925	-	-	1	1
*Acromyrmexsubterraneus* (Forel, 1893)	1	1	4	6
*Apterostigmaierense* Weber, 1937	-	-	1	1
*Apterostigmapilosum* Mayr, 1865	4	-	2	6
*Basicerosdisciger* (Mayr, 1887)	2	-	-	2
*Cardiocondylaminutior* Forel, 1899	1	-	-	1
*Carebarabrevipilosa* Fernández, 2004	-	2	-	2
*Crematogastermontana* Borgmeier, 1939	1	-	-	1
*Crematogasternigropilosa* Mayr, 1870	-	-	1	1
*Cyphomyrmexminutus* Mayr, 1862	1	2	4	7
*Cyphomyrmexrimosus* (Spinola, 1851)	3	4	10	17
*Hylomyrmareitteri* (Mayr, 1887)	11	5	9	25
*Lachnomyrmexplaumanni* Borgmeier, 1957	1	-	-	1
*Megalomyrmexgoeldii* Forel, 1912	6	13	12	31
*Mycetomoelleriusoetkeri* (Forel, 1908)	-	5	-	5
*Octostrumaiheringi* (Emery, 1888)	-	-	1	1
*Octostrumarugifera* (Mayr, 1887)	4	5	12	21
*Oxyepoecusmyops* Albuquerque & Brandão, 2009	1	-	-	1
*Pheidoletijucana* Borgmeier, 1927	2	-	-	2
*Rogerialacertosa* Kempf, 1963	2	-	-	2
*Sericomyrmexbondari* Weber, 1938	-	2	1	3
*Sericomyrmexsaussurei* Emery, 1894	1	8	6	15
*Strumigenyscosmostela* Kempf, 1975	-	-	1	1
*Strumigenyscrassicornis* Mayr, 1887	2	-	-	2
*Strumigenysdenticulata* Mayr, 1887	7	15	13	35
*Strumigenyselongata* Roger, 1863	-	1	1	2
*Strumigenyshindenburgi* Forel, 1915	1	1	1	3
*Strumigenyssubedentata* Mayr, 1887	-	-	1	1
*Paratrachymyrmexcornetzi* (Forel, 1912)	-	2	-	2
*Wasmanniaaffinis* Santschi, 1929	3	10	1	14
*Wasmanniaauropunctata* (Roger, 1863)	-	-	15	15
*Wasmannialutzi* Forel, 1908	3	4	-	7
**Pseudomyrmicinae**				
*Pseudomyrmexschuppi* (Forel, 1901)	-	1	-	1
**Ponerinae**				
*Anochetusmayri* Emery, 1884	3	7	-	10
*Hypoponeradistinguenda* (Emery, 1890)	1	0	2	3
*Hypoponeraforeli* (Mayr, 1887)	12	3	9	24
*Hypoponeraleveillei* (Emery, 1890)	2	-	1	3
*Hypoponeraparva* (Forel, 1909)	12	6	4	22
*Hypoponeratrigona* (Mayr, 1887)	16	3	12	31
*Hypoponeraviri* (Santschi, 1923)	10	4	6	20
*Leptogenysluederwaldti* Forel, 1913	2	-	4	6
*Neoponeraverenae* (Forel, 1922)	-	6	1	7
*Odontomachusaffinis* Guérin-Méneville, 1844	2	1	2	5
*Odontomachusbrunneus* (Patton, 1894)	0	1	1	2
*Odontomachuschelifer* (Latreille, 1802)	2	3	2	7
*Odontomachusmeinerti* Forel, 1905	1	5	2	8
*Pachycondylaharpax* (Fabricius, 1804)	2	2	15	19
*Pachycondylastriata* Smith, F., 1858	19	13	10	42
**Proceratiinae**				
*Discothyreaneotropica* Bruch, 1919	4	1	-	5
